# Large-scale Probabilistic Functional Modes from resting state fMRI

**DOI:** 10.1016/j.neuroimage.2015.01.013

**Published:** 2015-04-01

**Authors:** Samuel J. Harrison, Mark W. Woolrich, Emma C. Robinson, Matthew F. Glasser, Christian F. Beckmann, Mark Jenkinson, Stephen M. Smith

**Affiliations:** a Oxford Centre for Functional Magnetic Resonance Imaging of the Brain (FMRIB), Oxford, UK; b Oxford Centre for Human Brain Activity (OHBA), Oxford, UK; c Life Sciences Interface Doctoral Training Centre (LSI-DTC), Oxford, UK; d Department of Anatomy and Neurobiology, Washington University, Medical School, St. Louis, MO, USA; e Donders Institute for Brain, Cognition and Behaviour, Radboud University Nijmegen, The Netherlands

**Keywords:** Resting state fMRI, Functional parcellation, Bayesian modelling, Subject variability, ICA

## Abstract

It is well established that it is possible to observe spontaneous, highly structured, fluctuations in human brain activity from functional magnetic resonance imaging (fMRI) when the subject is ‘at rest’. However, characterising this activity in an interpretable manner is still a very open problem.

In this paper, we introduce a method for identifying modes of coherent activity from resting state fMRI (rfMRI) data. Our model characterises a mode as the outer product of a spatial map and a time course, constrained by the nature of both the between-subject variation and the effect of the haemodynamic response function. This is presented as a probabilistic generative model within a variational framework that allows Bayesian inference, even on voxelwise rfMRI data. Furthermore, using this approach it becomes possible to infer distinct extended modes that are correlated with each other in space and time, a property which we believe is neuroscientifically desirable.

We assess the performance of our model on both simulated data and high quality rfMRI data from the Human Connectome Project, and contrast its properties with those of both spatial and temporal independent component analysis (ICA). We show that our method is able to stably infer sets of modes with complex spatio-temporal interactions and spatial differences between subjects.

## Highlights

• We introduce a probabilistic model for modes in resting state fMRI.
• Our hierarchical model captures subject variability and haemodynamic effects.
• We illustrate its performance on simulated data and rfMRI data from 200 subjects.
• We demonstrate the ability of our method to infer spatio-temporally interacting modes.

## Introduction

Using resting state fMRI it is possible to generate enormously rich data sets that capture some of the complexity of the brain's intrinsic dynamics and connectivity. However, generating representations that meaningfully simplify the data, while still capturing these dynamics, is an immensely challenging problem.

Initial analyses of rfMRI data focused on finding regions of highly correlated activity (Biswal et al., 1995), with spatial independent component analysis (sICA) coming to prominence as a robust method for extracting regions consistent with knowledge from task analyses (Kiviniemi et al., 2003; Smith et al., 2009).

Recently, there has been much interest in techniques which analyse functional connectivity across the brain, including the potentially time-varying or non-stationary nature of these connections (E.A. Allen et al., 2014; Baker et al., 2014; Cribben et al., 2012; Seghier and Friston, 2013). However, for all but the simplest analysis techniques it is necessary to work in a lower dimensional space than the hundreds of thousands of voxels in a typical rfMRI data set. This is typically achieved either by extracting parcels from an anatomical atlas, or using high-dimensional sICA (Kiviniemi et al., 2009; Smith et al., 2013a). However, it is well known that “[i]nconsistent or imprecise node definitions can have a major impact on subsequent analyses” (Fornito et al., 2013), which again throws the question of how best to generate meaningful representations of resting state activity into sharp relief.

Therefore, an aim has become to find an interpretable and robust way of representing rfMRI data, at the same time as capturing as much of the complex temporal dynamics as possible.

### Definitions

For this paper, we will use the following definitions. We will take a *network* to be a set of interacting elements—synonymous with the mathematical formalism of a graph as a set of nodes and edges. Functional connections, that is to say the edges between nodes, may vary in their presence and strength over time.

We define a *parcel* to be a set of voxels acting with a single representative time course. These are often derived from a ‘hard’ parcellation of grey matter into multiple non-overlapping regions (Rubinov and Sporns, 2010; Yeo et al., 2011; Craddock et al., 2012). However, given the trend for using components from a high-dimensional sICA for connectivity analyses (E.A. Allen et al., 2014; Kiviniemi et al., 2009; Smith et al., 2013a), we relax this definition slightly. In the spatial domain, a parcel is taken to represent a set of positive weights, potentially varying in magnitude, with limited overlap between different parcels. The definition we have given therefore allows, for example, blurred boundaries or parcels that contain bilaterally paired regions.

We define a *mode* as any spatial distribution over the brain that shares a common time course. This is similar to a parcel, but the definition is wider as this imposes no restrictions on the spatial properties. For example, multiple modes can be highly overlapping, and individual modes can include anti-correlated regions (meaning that some regions within the mode have a negative spatial weight and others have a positive one). A mode—as an extended spatial distribution having common temporal dynamics—can be defined either in terms of a spatial voxelwise map, or as a weighted set of spatial parcels.

In general, it is possible to take the time courses from either parcels or modes and use these as the nodes to examine in a subsequent network analysis, but we will focus on modes here.

### Current methods

Many techniques have been proposed to identify modes or parcels. Perhaps the simplest is to extract time courses from labelled regions in a pre-defined anatomical atlas, though the validity of this has been called into question as the correspondence between anatomical landmarks and functional regions is unclear (Fornito et al., 2013). The obvious alternative is to use a pre-defined atlas containing regions based on previous functional studies, an approach which is likely to have a higher validity.

However, the arguable weakness of atlas-based approaches is their reliance on the registration process to enforce consistency across subjects. There is an enormous amount of interesting structure present in rfMRI data, and it seems reasonable to assume that this could be harnessed to inform the specification of functional regions. In fact, one of the key assertions we make in this paper is that it is possible to attempt to use the characteristics of the rfMRI data to correct for subject mis-alignments.

There have therefore been a large number of strategies proposed that attempt to infer functional regions from the data—so called ‘data-driven’ approaches. Temporally consistent co-activation is the implicit assumption that defines both parcels and modes, but by itself this does not lead to a unique decomposition. Therefore, it is necessary to add additional constraints to make the inference problem identifiable.

The most widely used data-driven approach is to look for modes that are independent using ICA. Due to the large numbers of voxels and relatively few time points of early studies, spatial ICA gave the most robust decompositions and therefore became the dominant approach. However, almost as soon as it was introduced, concerns were raised. Given that “[distinct] large scale neuronal dynamics can share a substantial anatomical infrastructure” (Friston, 1998; Smith et al., 2012), it is unclear how well sICA will decompose extended modes that spatially overlap. These concerns were allayed to some extent by Beckmann et al., who showed that in the presence of noise ICA components can still contain strong residual dependencies, and highly correlated maps can be recovered by a simple thresholding step (Beckmann et al., 2005). What is perhaps less clear is what, if any, biases are introduced when the data has a high SNR or when large groups are analysed, both cases where the inferred maps are expected to contain very little noise.

An alternative approach is to look for temporally independent modes, which has recently become possible as studies of large cohorts acquired at low TR have generated enough time points for temporal ICA (tICA) to operate robustly (Smith et al., 2012), albeit still most likely requiring the concatenation of several fMRI data sets to achieve reasonable reproducibility. This allows spatially overlapping modes to be identified, at the expense of placing restrictions on the global temporal dynamics—as well as this being a concern in and of itself, this restriction will also limit any subsequent network analyses of the mode time courses. As Smith et al. discuss, temporally independent functional modes (TFMs) are forced to have orthogonal time courses, meaning that further analysis of the temporal interactions between different modes is not straightforward (Smith et al., 2012).

As well as the choice of spatial or temporal independence, various extensions have been proposed to extract meaningful subject-specific information from group ICA decompositions (Damoiseaux et al., 2006; Filippini et al., 2009; Varoquaux et al., 2010; Erhardt et al., 2011).

While each ICA strategy has its own advantages, the fundamental issue with all ICA-based approaches is that “it is not clear that, from a neuroscientific point of view, independence is the right concept to isolate brain networks, as no functional system is fully segregated” (Varoquaux et al., 2010). What is perhaps surprising is how demonstrably well ICA approaches work, given that their central assumptions are often violated (Hyvärinen, 2013); for example, forms of ICA have been developed that explicitly incorporate information derived from the residual statistical dependencies between components (Hyvärinen and Hoyer, 2000; Hyvärinen et al., 2001). Therefore, while ICA approaches have been particularly useful for characterising fMRI data, one would hope that a less restrictive set of assumptions could engender decompositions with even higher validities.

Other data-driven approaches suggested have had varying degrees of success. Many are based on machine learning techniques, where the key assumptions underpinning the algorithms are only loosely related to the expected properties of rfMRI data. These include clustering approaches (Yeo et al., 2011; Craddock et al., 2012), regularised variants of principal component analysis (PCA) (G.I. Allen et al., 2014), non-negative matrix factorisation (Lee et al., 2011), image gradient detection in correlation maps (Cohen et al., 2008) and hidden Markov models (Eavani et al., 2013) to name but a few.

Finally, there are a few approaches which try to explicitly model rfMRI data. The multi-subject dictionary learning (MSDL) approach of Varoquaux et al. (2011) forms a model that explicitly looks for modes/parcels, and there are some conceptual similarities with our approach. Their algorithm contains a hierarchical model for spatial subject variability, a constraint favouring simultaneously smooth and sparse spatial distributions as well as the ability to capture the temporal correlations between modes.

Due to the similarities between our approaches, we will give a brief description of the most recent version of their model, of which more details can be found in the work of Abraham et al. (2013). Their spatial model at the group level is detailed, simultaneously enforcing non-negativity, sparsity and spatial contiguity. The subject maps are modelled by including a set of additive, Gaussian-distributed deviations from the group maps. Their time series model specifies that there should be a consistent between-mode correlation structure but does not restrict the form of the time series; therefore, it does not model any haemodynamic processes. Finally, these constraints are combined with a noise model, and the resulting cost-function governing their decomposition is solved with a computationally efficient stochastic gradient descent approach. The most recent results they report are on an rfMRI data set consisting of 48 subjects.

In this paper, we develop an analysis technique that explicitly models some of the key properties of resting state modes within a Bayesian framework. The Bayesian approach allows very flexible models to be constructed in a principled manner; crucially, we solve the system using a variational approach, thereby making the algorithm efficient enough to work on full fMRI data sets.

The paper is structured as follows. First, we describe our model and the approaches needed to maintain tractability when doing Bayesian inference. We then present our results, including both verification on simulated data and details of the modes inferred on fMRI data from the Human Connectome Project (Van Essen et al., 2013) (HCP). Finally, we discuss the implications of our results and key areas for future investigation.

## Model

We aim to identify extended functional modes which are not restricted to being orthogonal, or non-overlapping, to each other in space or in time. In general terms, we define a hierarchical model that allows us to flexibly capture the spatial variation of modes across subjects, while still keeping track of key properties at the group level. We simultaneously enforce that the temporal characteristics of modes must relate to the haemodynamics that drive the BOLD signal. Finally, by doing inference in a Bayesian setting, the extent to which our modes explain the data is automatically traded off against how well they align with our prior beliefs.

As the modes we infer are defined by the generative model outlined below, we will refer to them as probabilistic functional modes (PFMs).

The model is built on the same matrix factorisation approach that underpins PCA, ICA, non-negative matrix factorisation and several other well established methods for analysis of rfMRI. The approach is to decompose an fMRI run into the product of two matrices, which in the case of rfMRI are interpreted as a set of mode spatial maps and time courses respectively.

The fMRI data are acquired with *V* voxels and *T* time points, giving the data matrix ***D***_*V* × *T*_ for a single run. If we infer a set of *M* PFMs then we look for spatial maps, ***P***_*V* × *M*_, and time courses ***A***_*M* × *T*_. In general we infer a small number of PFMs relative to *V* and *T*, which gives a parsimonious set of modes. However, this means that the factorisation will not be exact, so we express the data as the contribution from the PFMs and a noise term, *ε*, to give

(1) D=PA+ε.

In order to infer both the group-level properties and any interesting subject variability, we explicitly account for the full set of all runs, D. This contains data from each subject within the set of subjects *S*, and in general each subject, *s*, will have a set of multiple runs *R_s_*.

(2) $$D={\left\{{\left\{D^{\left(\mathrm{sr}\right)}\right\}}_{r\in R_{s}}\right\}}_{s\in S}$$

We then assume that the noise and time courses will be randomly varying in each run, but that each subject has a set of mode spatial maps that are consistent across all their runs. This extends Eq. (1) to give

(3) $$D^{\left(\mathrm{sr}\right)}=P^{\left(s\right)}A^{\left(\mathrm{sr}\right)}+\varepsilon^{\left(\mathrm{sr}\right)}\text{.}$$

To formulate this as a probabilistic model we place priors on both the PFM spatial maps and time courses, as well as modelling the contribution from the noise. The forms of these distributions are explained in the following sections.

### Spatial prior

For each voxel *v* in the spatial map of PFM *m* we want to assess whether, given the noise level and time course, the data supplies enough evidence to suggest that there is genuinely an effect present in that voxel. There is a direct conceptual link between this approach and the traditional approach of significance testing within the general linear model framework for task fMRI data.

If there is insufficient evidence a posteriori for an effect we should just set the weight at that voxel to zero. However, if there is evidence for an effect, then we are interested in both its size and how it varies over subjects.

**Fig. 1:**
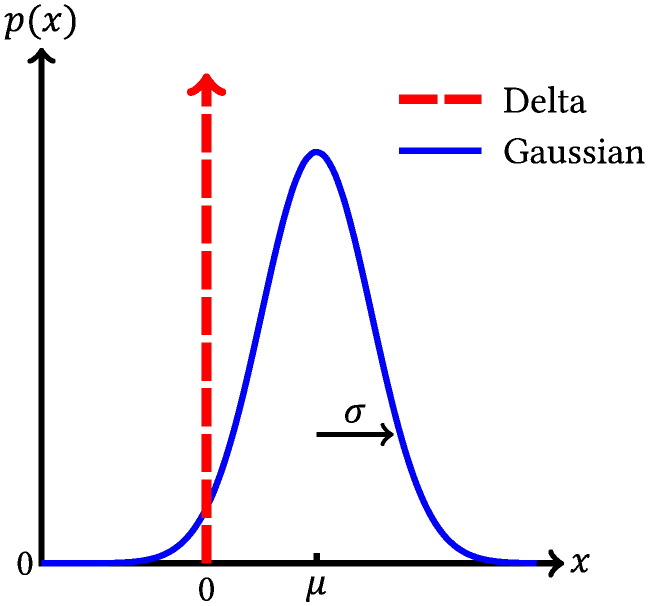
Diagrammatic representation of the probability density function for the delta-Gaussian mixture model. The relative contribution of each component is governed by the parameter π, while the Gaussian component is parameterised by its mean, *μ*, and standard deviation, *σ*.

To express this model probabilistically, we formulate a delta-Gaussian mixture model, a natural extension of the spike-slab distribution (Titsias and Lzaro-Gredilla, 2011). This contains a delta component at zero to capture the effects that are not present or too weak to be observed, and a Gaussian to model the observable effects and their variability over subjects. This is parameterised by the probability of an effect being present, π, as well as the mean, *μ*, and standard deviation, *σ*, of the Gaussian. There is also a binary indicator variable, *q*, to capture which component each weight is drawn from. Crucially, we have placed no explicit prior on the relationship between the spatial distributions of different modes, so there is no explicit penalty on voxels being present in multiple modes. Similarly, the fact that the non-zero weights are drawn from a Gaussian means that they can be either positive or negative, thereby allowing modes containing anti-correlated regions—if the data supports that inference. This has the form given in Eq. (4) and is shown graphically in Fig. 1. Note that the inference on this model automatically combines the evidence from each of the runs available for a given subject, weighted to take into account the signal to noise ratio of the time courses.

(4) pPvms|qvms=1=NPvms|μvm,σvm2pPvms|qvms=0=δPvmspqvms=πvmqvms1−πvm1−qvms

The three parameters at each voxel, π, *μ* and *σ*, parameterise the distribution of the observed spatial maps over subjects. They succinctly capture our beliefs about the answers to three very pertinent questions: does a voxel contribute to a given PFM? If so, how big is the contribution and how much does it vary from subject to subject?

As is standard, we place a beta-hyperprior on π and an inverse gamma hyperprior on *σ*. Finally, we place a spike-slab hyperprior on each mode's voxelwise means, with precision *γ* and sparsity *λ*, as in Eq. (5).

(5) pμvm|ρvm=1=Nμvm|0,γm−1pμvm|ρvm=0=δμvmpρvm=λρvm1−λ1−ρvm

The spike-slab hyperprior allows us to regularise the group spatial maps, if required, by altering the *λ* parameter. Intuitively, *λ* represents the proportion of voxels we expect to be non-zero a priori in each mode's group level spatial map. In reality, as we expect modes to be spatially distributed and overlapping, we set *λ* > *M*^− 1^.

Note that when we present group maps we show the marginal posterior mean of the whole spatial distribution, $E\left[\rho_{vm}\mu_{vm}|D\right]$, rather than just the *μ* parameters.

### Temporal prior

When looking at rfMRI data, the spectral characteristics of the neurally-driven BOLD signal are dominated by the haemodynamic response function (HRF). In other words, we know that any non-artefactual mode time courses we observe will be dominated by low frequencies, almost regardless of the frequency content of the underlying neuronal processes.

Therefore, we formulate a temporal prior that captures the auto-correlation induced by the HRF. For a neuronal signal, *x*(*t*), and a linear HRF, *h*(*t*), the observed signal *y*(*t*) is a simple convolution of *x*(*t*) and *h*(*t*). If we assume that the neuronal process is white on the time scale of the fMRI acquisitions then it is straightforward to show that the auto-correlation induced in the observed signal is just the auto-correlation of the HRF, namely

(6) $$E\left[y\left(t_{1}\right)y\left(t_{2}\right)\right]=\sum_{\tau }h\left(\tau \right)h\left(\tau -\left(t_{1}-t_{2}\right)\right)\text{.}$$

We assume a canonical double-gamma HRF and use this correlation structure to construct a full covariance matrix, ***K_A_***, for all the time points in a given run. We also place a standard inverse gamma hyperprior, *α*, on the overall precision. Therefore, the prior on the time course for PFM *m*, which we denote ***A***_*m*_^(*sr*)^ ∈ *ℝ*^1 × *T*^, becomes

(7) $$p\left(A_{m}^{\left(\mathrm{sr}\right)}|\alpha \right)=N\left(A_{m}^{\left(\mathrm{sr}\right)}|0,\alpha^{-1}K_{A}\right)\text{.}$$

Of course, it is well known that the HRF is both highly variable and much more complex (Aguirre et al., 1998; Handwerker et al., 2004; Kriegeskorte et al., 2010) than the canonical linear HRF assumed here. However, this is a probabilistic prior, rather than a hard constraint, so there is scope for the inferred time courses to match the temporal structure in the data. Similarly, this form of prior on the covariance structure is predicated on a white neuronal process. Some current theories, based on evidence from electrophysiology (Siegel et al., 2012) and biophysical models (Deco et al., 2011), predict that the neural basis of rfMRI activity is correlations between the envelopes of higher frequency signals, where these amplitude correlations occur at ‘ultraslow’ timescales (biased towards frequencies less than 0.1 Hz). On the other hand, current evidence suggests that neuronal process are white over the range of frequencies estimable at typical fMRI sampling rates (Niazy et al., 2011) and that structure, consistent with the rfMRI literature, is present at surprisingly high frequencies (Baker et al., 2014).

Given the wide range of temporal models present in the literature, we make the pragmatic decision to choose a very simple linear model to capture the gross properties of the HRF and rely on the Bayesian inference procedure to be flexible enough to capture some of the more complex properties. This also has the advantage of being computationally efficient.

### Noise model

The final part of the model to specify is the noise. This is simply white Gaussian noise with a mean for each voxel, *v*. The overall noise precision for each run, *ψ*, takes a standard gamma hyperprior, while the mean has a Gaussian hyperprior.

(8) $$\begin{array}{c} p\left(\varepsilon_{t}^{\left(\mathrm{sr}\right)}\right)=N\left(\varepsilon_{t}^{\left(\mathrm{sr}\right)}|\nu^{\left(\mathrm{sr}\right)},{\left(\psi^{\left(\mathrm{sr}\right)}\right)}^{-1}I\right)\\ =p\left(D_{t}^{\left(\mathrm{sr}\right)}-P^{\left(s\right)}A_{t}^{\left(\mathrm{sr}\right)}\right)\text{.} \end{array}$$

### Variational inference

The aim is then to carry out Bayesian inference on this model. However, calculating the full posterior analytically is intractable and a sampling procedure for a model with this many parameters would be prohibitively slow.

Therefore, we use a variational approach. In this framework the posterior is chosen to take a simplified, parameterised form, with the parameters chosen to best approximate the true posterior. We denote the simplified posterior distributions *q*(*x*), and in this case the approximation we make is to factorise the posterior over the variables, **Θ**, to give

(9) $$p\left(\Theta |D\right)\approx \prod_{\theta \in \Theta }q\left(\theta \right)\text{.}$$

While this is an approximation, it is very efficient and allows us to do Bayesian inference over large sets of rfMRI runs simultaneously.

Of course, while this approximation allows us to proceed, it introduces a deviation from the true posterior and the implications of this are very hard to quantify. This reinforces the need to evaluate the results critically. Our tests with simulations and real data, described later, lead us to believe that the effects of this approximation are not too pernicious.

As we have taken all of our distributions from the conjugate exponential family the variational update rules are somewhat routine (Winn et al., 2005); therefore, we will not provide them in the body of this paper but in the Supplementary material. This also contains the graphical model and the values of the parameters we use for the prior distributions for both simulations and real data.

### Model identifiability

The identifiability of our decomposition is guaranteed by virtue of a non-Gaussian spatial prior, as is the case with both sICA and MSDL, amongst many others. However, this does not imply an equivalence between decompositions as all three models have fundamentally different formulations of this spatial prior. Furthermore, compared to sICA, both our approach and MSDL explicitly model subject variability,

Our HRF-based prior on the time courses strongly predisposes the decomposition to identify modes that are neural in origin, rather than those which represent structured artefacts. However, the time course prior is rotationally invariant across modes. Therefore, it can only help identify the subspace the BOLD signal resides in, but crucially does not aid in the unmixing of the modes. This limitation arises because of the assumption of a linear HRF operating on independent neural time courses. Intuitively, if the set of inferred time courses are all consistent with the frequency spectrum implied by our HRF, then any linear combination of said time courses would satisfy our temporal constraints equally well.

Finally, our method works on the full data. ICA, for example, normally operates in a reduced PCA subspace, of the same rank as the final decomposition, and is therefore completely reliant on the PCA step to separate the BOLD signal and the noise. It seems somewhat unlikely that the PCA step will perfectly separate the two, and this is deleterious as the ICA components will be contaminated by any noise that enters the PCA subspace, while they will be ‘blind’ to any genuine signal that is excluded by the PCA step. While there is a computational trade-off, a more holistic approach, modelling both the BOLD signal and the noise simultaneously, seems to be attractive.

## Results

### Simulated data

In order to test the efficacy of our method we simulated an fMRI data set containing an embedded ground truth set of modes. This allows us to compare the accuracy of our model against other commonly used approaches, under the assumptions of the simulations.

#### Data generation

The data was simulated to represent a scaled-down version of the HCP acquisition protocols, where each subject was scanned four times for 15 minutes at a TR of 0.72 s, yielding 4800 time points per subject. However, as we are looking to repeatedly simulate data of this form, only 12,500 voxels and 30 subjects—representing 144,000 time points—are simulated. This is compared to the actual set of 90,000 HCP ‘grayordinates’ and over 500 subjects released to date. This represents a data reduction by approximately a factor of 7 in both the spatial and temporal domains compared to the HCP data set we use in subsequent analyses. While somewhat arbitrary, this yields test data sets that are computationally manageable, while having the same ratio of spatial to temporal data points as in the HCP and where the various test–retest reliabilities are consistent with what we observe on the real data.

**Fig. 2:**
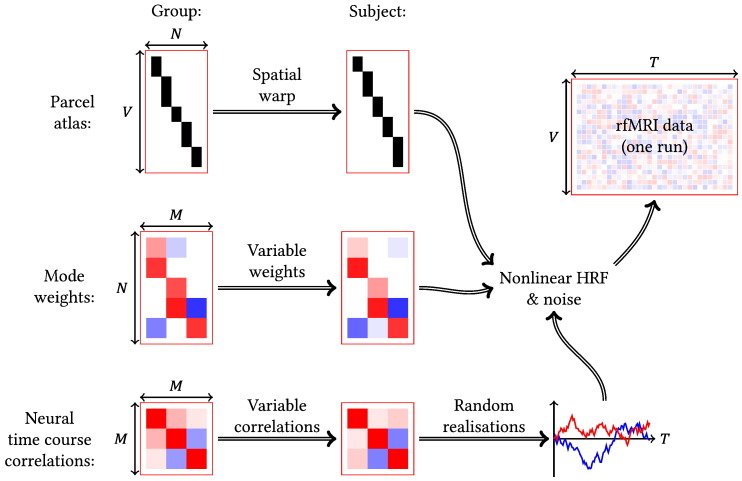
Procedure for generating our simulated rfMRI data. *V*: voxels; *T*: time points; *N*: parcels; *M*: modes.

The mode activity itself was simulated as follows and this process is outlined schematically in Fig. 2. What follows is a brief description of the salient properties of our simulations. A much more detailed account, along with all the parameter values used and some examples of typical simulated data, can be found in the Supplementary material.

Firstly, a spatial ‘atlas’, of 200 binary, non-overlapping, continuous, random parcels was generated, mimicking a one-dimensional hard parcellation model. Random, smooth spatial warps were applied to generate subject-specific versions of the group atlas, with the maximum possible spatial shift limited to 1.5 times the average parcel width—in this case just under 100 voxels. These warps are meant to be a simplified representation of both instances where the registration does not bring regions into alignment, and instances of genuine topological reorganisation.

The spatial distribution of 25 modes was specified as a sparse set of weights, describing how strongly each parcel was associated with each mode. The weights could be positive or negative, thereby allowing modelling of within-mode anti-correlations. There was also a preference for mode weights to be similar in adjacent parcels. This generates modes with greater continuous spatial extent than individual parcels, increasing the robustness to misalignments at the parcel level. The mode sparsities were beta-distributed, with mean 0.08 and variance 7.5 × 10^− 4^. Each mode is therefore associated with about 16 parcels on average or, in other words, each parcel was present in about 2 modes. Finally, subject-specific mode weights were generated by adding a sparse set of deviations around the group weights. A simple matrix multiplication of the parcel atlas by the mode weights gives the mode maps in voxel space.

In the temporal domain, mode time courses were simulated before the action of the HRF, at a temporal resolution of 0.1 s. These time courses were not white, but had an increased power spectral density at less than 0.1 Hz; as mentioned previously, this allows us to investigate how our model performs when the implicit assumption of a white neuronal process is violated. There was a group-level pattern of temporal correlations between modes, though there were variations at both the subject and run level. Finally, the time courses were thresholded based on amplitude, such that the smallest 80% of time points were set to zero, to introduce temporal non-Gaussianities.

**Fig. 3:**
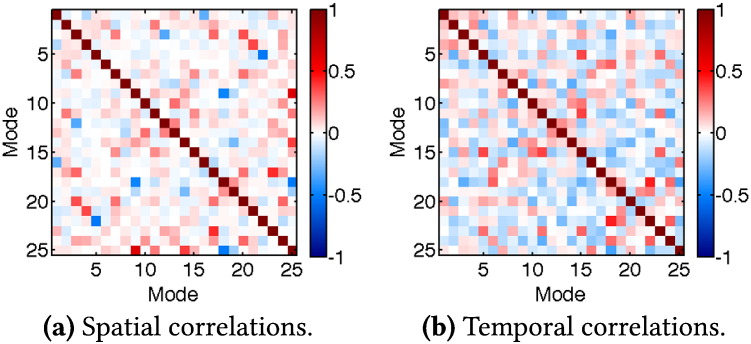
Examples of the ground truth spatial and temporal between-mode correlation coefficients for one subject in the simulated data tests.

To illustrate the strength of the induced correlations between modes, we plot a set of both spatial and temporal correlation coefficients from a single simulated subject in Fig. 3. Note that the presence of non-zero correlations between the ground truth modes, both spatially and temporally, goes against prior constraints in PCA/ICA models, but we believe that this makes neuroscientific sense.

In order to generate the BOLD signal, the neuronal activity in each voxel was calculated using the mode time courses, weights and the spatial atlas. These voxel time courses were then convolved with a linear HRF—this was randomly drawn from a basis set and varied over both subjects and space (Woolrich et al., 2004). A nonlinear saturation function, loosely based on a second-order Volterra kernel approximation (Friston et al., 1998), was then applied. Finally, spatio-temporally white noise was added to achieve an overall signal to noise ratio of − 10 dB.^1^

#### Methods and scoring

We compared the PFMs inferred by our method to the modes recovered by PCA, sICA, tICA and stICA. The stICA approach has two main steps: sICA is used to find the 200 parcels in the spatial atlas, before tICA is used to identify the modes in this reduced space. The results from these two steps are then combined to recover the mode maps in voxel space and, like the other methods, stICA is only scored based on the recovery of the modes. Note that stICA was the approach used by Smith et al. when characterising TFMs with tICA (Smith et al., 2012). The ICA techniques used the FastICA toolbox (Hyvärinen, 1999) and were compared before and after a final dual regression step (Beckmann et al., 2009), a technique for estimating subject-specific versions of group-level modes.

The results we present here are from tests where all methods were tasked with identifying the set of 25 ground truth modes. Of course, it is specious to assume that the ‘true’ number of modes is known, so the Supplementary material contains results for cases where methods were asked to recover either 15 or 40 modes. This allows us to assess how robust the methods are to misspecification of the number of modes, which is likely to be the case on real data.

Methods were scored on how accurately they recovered the subject-specific spatial maps and time courses, as well as how accurately they could recover the between-mode spatial and temporal correlation structures. Furthermore, the methods were run twice on each simulated data set. This allowed us to assess the relationship between test–retest reliability and accuracy relative to the ground truth.

We use a correlation coefficient to score the accuracy of the recovery of the spatial maps and time courses for the different methods. For this paper we use the sample Pearson correlation coefficient, with the slight modification that we do not remove means. We have taken this approach because the matrix factorisation, while being ambiguous with regard to the relative scalings of the maps and time courses, is not invariant to shifts in the means. Modes were matched to the ground truth using the Hungarian algorithm (Kuhn, 1955) based on the sum of the map and time course scores.

Once modes have been paired with their nearest ground truth counterparts, we calculate the decomposition accuracies. In the spatial domain this is the correlation between the inferred and true versions of the subject-specific maps, and the correlations between true and estimated time courses in the temporal domain. We present the mean, over subjects, of these correlations for each mode in the figures.

In order to evaluate how well methods recovered the inter-mode correlation structures, we recorded the RMS error between each element of the inferred and ground truth correlation matrices; again, this is calculated separately for spatial and temporal correlation coefficients and takes into account the pairing of the inferred modes to the ground truth. In this case, the mean used to calculate the RMS error is again taken over subject-specific correlation matrices.

Test–retest scores were calculated using the same method as above, except that rather than comparing the ground truth and inferred mode maps, the two sets of maps resulting from different random initialisations of the various algorithms were matched and scored. Note that test–retest reliability is distinct from, though closely related to, split-half reproducibility, a metric commonly used to evaluate the reliability of components from real data (Groppe et al., 2009). As we use these simulations to evaluate the relationship between test–retest reliability and ground truth accuracy, we will persist with this as our performance metric on real rfMRI data.

#### Results

**Fig. 4:**
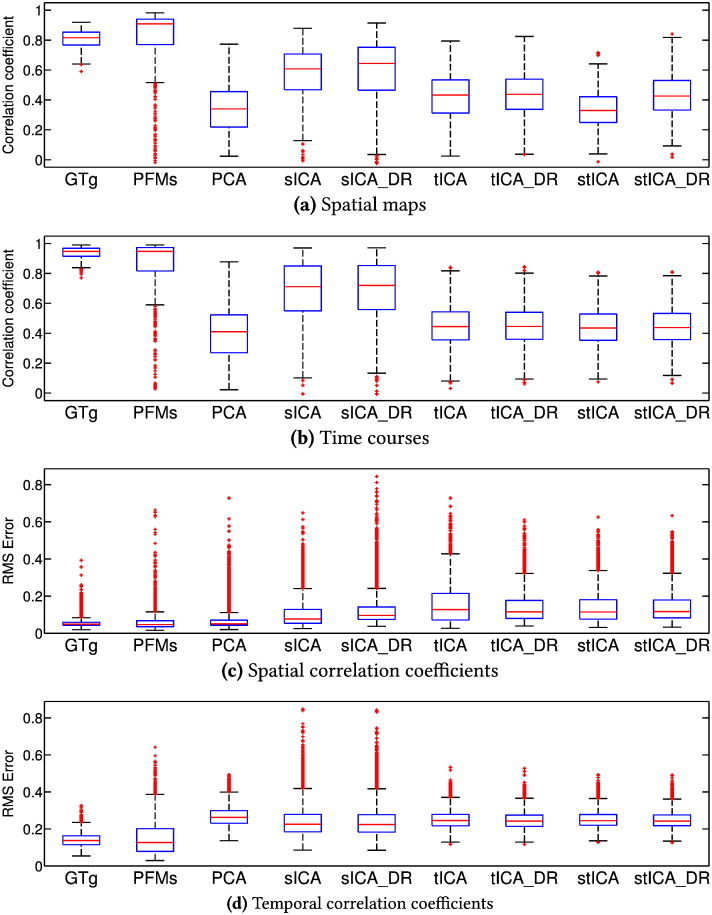
Accuracy in recovery of the ground truth on simulated data. Multiple data sets were simulated, and the accuracy with which the above data characteristics are inferred, for each of the 25 modes, is shown for each method. Dual regression is indicated by the suffix DR. GTg illustrates the scores that are achieved if the subject maps are just set to the mean of the ground truth subject maps; as such, it both illustrates the amount of subject variability in the data and is also a useful benchmark for those methods which do not model individual subjects.

**Fig. 5:**
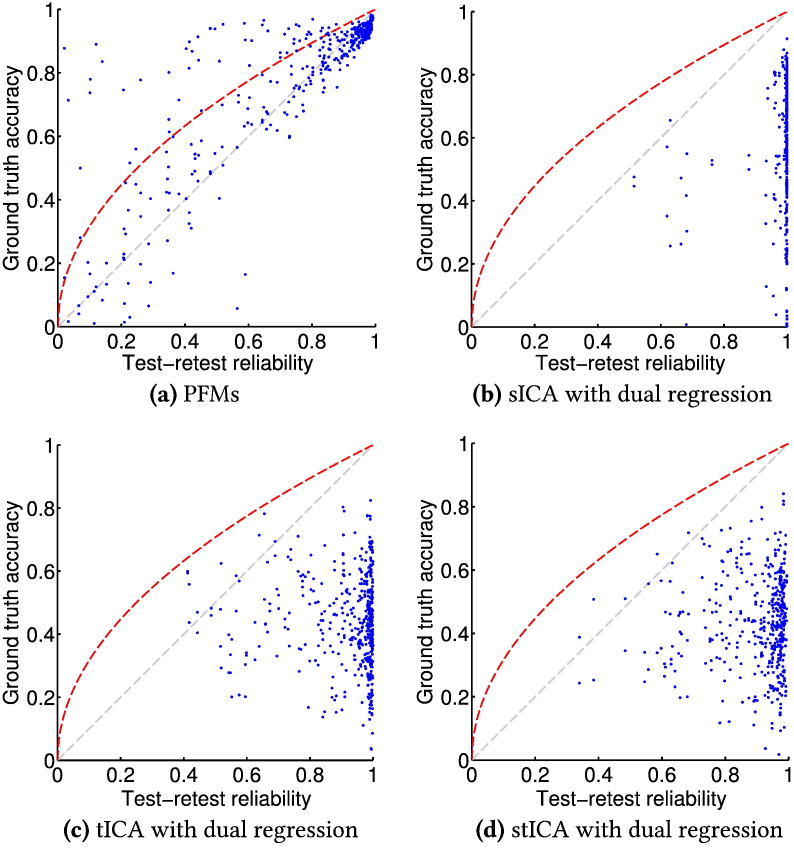
Accuracy in recovery of ground truth subject-specific spatial maps plotted against test–retest reliability for four methods tested on simulated data. Each method was run twice on the same data set; both the accuracy scores, as plotted in Fig. 4a, and the test–retest reliability, scored using the same correlation metric, were calculated for each mode. The grey line indicates equality between the two scores, whereas the red line indicates the range of scores possible if the inferred maps are just the ground truth maps with independent additive noise.

Fig. 4 contains the scores that capture how accurately the various methods recovered the ground truth modes. These results are compared with the test–retest reliability for four of the methods we tested in Fig. 5.

Out of the traditional ICA approaches, sICA is by some distance the most accurate. This is perhaps somewhat surprising given that these simulations are based around modes, rather than parcels, and that there are far more time points than voxels. However, the spatial map distributions are strongly non-Gaussian and the sICA decompositions are very robust, leading us to believe that, for these simulations, the limiting factor for sICA is the restriction that the solution has to lie on the manifold of uncorrelated maps.

Both tICA and stICA perform relatively poorly. It has long been known that with small numbers of time points tICA is much less robust than sICA (Friston, 1998; McKeown et al., 1998); however, each of our simulated data sets has over five times as many time points as the data set used in the paper which introduced tICA as a method for identifying modes from rfMRI data (Smith et al., 2012), so we would not expect this to be the limiting factor. Again, restricting the search space to temporally uncorrelated modes is bound to be detrimental. However, this problem is compounded by the blurring action of the HRF severely reducing the strength of the observable temporal non-Gaussianities. Finally, in this case the two-step stICA approach does not seem to improve performance compared to running tICA directly. However, it seems likely that some of the key benefits of this approach on real data, for example the ability to remove artefactual components at the sICA stage or to renormalise parcel time courses that are acquired in regions with different sensitivities, are not being fully captured by our simulations.

For all the ICA approaches, dual regression allows the inference of subject specific maps and time courses, offering only modest improvements in performance in the spatial domain.

Our method performs the most accurately out of the various methods on test here—it is able to infer both temporally and spatially correlated features reliably from this data. However, what is particularly encouraging is the lack of systematic bias in the inferred PFMs, as illustrated in Fig. 5a. Ground truth accuracy is strongly correlated with test–retest reliability, a property one expects where estimation is accurate, and is particularly useful as the latter is usually the only performance metric available on real data. In contrast, for all the ICA methods repeatability is essentially independent of accuracy; the ICA algorithms show reliable convergence to relatively inaccurate solutions. Though it is an obvious point, it is one that is perhaps worth repeating: test–retest reliability is only a necessary, rather than sufficient, condition for successful inference.

The Supplementary material contains the equivalent set of results when the algorithms are asked to infer a different number of modes to the set in the ground truth. What is striking is how consistent the relative accuracy of the methods is. This suggests that when there is a mismatch in dimensionality, as is likely the case on real data, the results can still be trusted. Similarly, if we look at the recovery of the BOLD subspace we can see that the PFMs recover this much more consistently as dimensionality varies. For the methods that rely on PCA to find the subspace, as dimensionality increases more variance is explained. The issue is that if this extra variance represents noise the accuracy will actually reduce. However, for the PFMs there is an explicit prior on haemodynamic time courses which seems to suppress the amount of noise introduced into the subspace by the extra components.

In summary, the results from our simulations lead us to believe that the PFMs our method infers have interesting and valid spatial and temporal interactions. Furthermore, we believe that, for our method, test–retest reliability will be a useful indicator of accuracy on real rfMRI data.

### HCP data

For the subsequent analyses we use rfMRI acquired as part of the HCP—full details of the various acquisitions can be found in publications from the project (Van Essen et al., 2012; Smith et al., 2013b).

Each subject has four 15-minute rfMRI runs, acquired using multi-band acceleration with a TR of 0.72 s to give 1200 time points per run. The runs are sampled into the standard set of just over 90,000 grayordinates and then FIX cleaned (Salimi-Khorshidi et al., 2014; Griffanti et al., 2014) to automatically remove as many structured artefacts as possible. These cleaned runs are then aligned using MSM (Robinson et al., 2013, 2014), a flexible framework for surface-based registration based on information from multiple modalities. In this case, registration is driven by both structural and functional features. Cortical folding patterns and myelin maps were used as the structural features. The functional features were subject-specific low dimensional sICA components, extracted by running ICA on the group followed by dual regression.

We used all 209 of the subject data sets publicly available at the time these analyses were run.

Note that the set of grayordinates includes both cortical and subcortical structures, and all algorithms were run on the full set of vertices. However, the following figures only show the results on the cortical surfaces.

#### Test–retest reliability

**Fig. 6:**
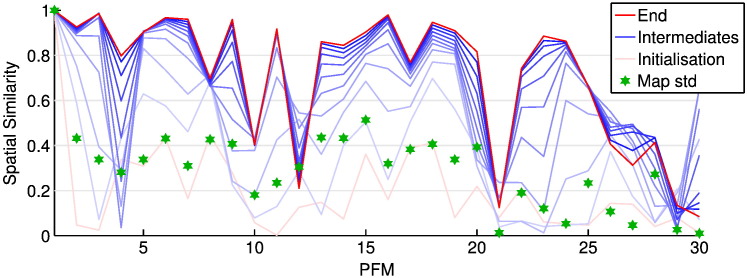
Plot showing the convergence of PFM maps over two separate runs of our algorithm. The similarity is measured by the correlation coefficients between the best matched group-level maps from each analysis. The algorithm was run for 1000 iterations; a blue line is plotted after every 100 iterations with darker lines indicating more iterations. We also plot an indication of the strength of the spatial weights in the various maps. For each analysis we calculate the standard deviation of the map weights for each PFM, before normalising these by the size of the global component (PFM 1). Then, after the PFMs have been matched, we plot the maximum standard deviation for each pair. A value of close to zero suggests a pairing between maps that have been eliminated from the model by the implicit Bayesian model regularisation.

In order to evaluate how stable our algorithm is on real data we ran two analyses, both looking to extract 30 PFMs from the full set of available HCP data. The convergence of the PFM maps, from different random initialisations, is shown in Fig. 6. The PFM numbers we assign here are used consistently in all subsequent sections. The maps themselves can be found in the Supplementary material.

The choice of 30 PFMs was somewhat arbitrary, though it did seem to give sets of modes that related, to a reasonable extent, to some spatial maps reported in the literature. If the dimensionality was increased, to the region of 100 PFMs, we observed that a set of PFMs with a similarly large spatial extent was inferred, often closely matching to the results presented here. The remaining PFMs were either eliminated from the model or seemed to capture subject-specific artefacts. This is an area that clearly warrants further investigation and we would hope to be able to reliably identify larger numbers of modes with more data sets and more iterations of the analysis.

As Fig. 6 shows, the algorithm clearly converges to a stable set of PFMs, though the Bayesian model regularisation automatically eliminates some of the thirty from the model. Twelve PFMs are inferred with a correlation coefficient of above 0.9, with a further five scoring above 0.8.

However, there are examples of plausible PFMs that are only present in one of the analyses. For example, PFMs 10 and 12 have low reliabilities despite having very plausible spatial distributions. By way of contrast, PFMs 26 to 30 are neither strongly reproduced nor consistent with previously reported results. This is exactly as we would expect given our simulated data results, where we observed that low reproducibility does not necessarily imply low accuracy. It is highly unlikely that a random initialisation will lead to the most stable decomposition, as several similar Bayesian methods have noted (Groves et al., 2011), but it is important to show that the algorithm can converge from disparate start points in parameter space, as demonstrated here.

#### Interactions between ‘cognitive’ modes

**Fig. 7:**
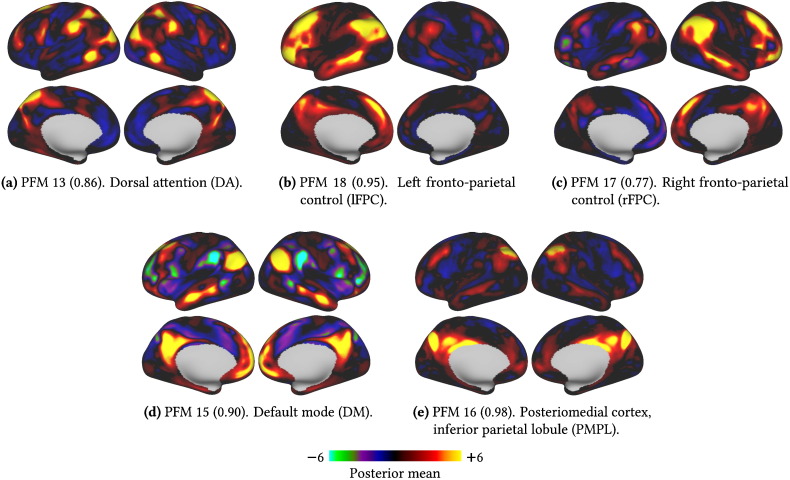
Five example group spatial maps for PFMs identified by our method. For each PFM we report the test–retest reliability score as well as the common name reported in the literature, if appropriate. Cortical surface views were generated using Connectome Workbench (www.humanconnectome.org/connectome/connectome-workbench.html). The relative scaling of the spatial maps and time courses is dependent, albeit weakly, on the specific prior parameters (given in the Supplementary material). Therefore, we have not applied any normalisations to the maps presented here, but have simply displayed the posterior mean with a representative colour encoding.

To illustrate the advantages of having a method that can flexibly infer both spatial and temporal interactions we will focus on five PFMs that relate to areas associated with higher cognitive functions. The spatial maps for these PFMs are shown in Fig. 7.

These are based on one of the analyses used to illustrate test–retest reliability, where we extracted 30 PFMs. The full set of extracted spatial maps is shown in the Supplementary material.

The dorsal (visual) attention system (DA, Fig. 7a) is associated with top down control of externally directed attention (Corbetta and Shulman, 2002). The default mode (DM, Fig. 7d) is thought to be involved in self-directed thought and background levels of attention (Raichle et al., 2001; Buckner et al., 2008). The third well-established mode is the fronto-parietal control system (FPC), thought to flexibly couple with either the DA or DM in order to mediate between internal and external attention (Vincent et al., 2008; Spreng et al., 2010; Cole et al., 2013). Our method splits this into left and right lateralised forms, which we will term the lFPC (Fig. 7b) and the rFPC (Fig. 7c).

Finally, we have also highlighted a mode which, like the DM, is strongly present in the posteromedial cortex (PMC) and inferior parietal lobule^2^ (PMPL, Fig. 7e). This is present in three distinct regions of the PMC and these are, from anterior to posterior, the retrosplenial cortex, the posterior cingulate cortex (PCC) and the anterior bank of the parieto-occipital sulcus (V6A (Sereno et al., 2013)/POS2 (Glasser and Van Essen, 2011)).

It is no great surprise that it is possible to identify several different modes that contain regions within the PMC, as this is an area of cortex that has repeatedly been associated with high functional heterogeneity. For example, patterns of resting state correlations have been shown to be strongly dependent on which area of the PMC is used as the seed (Margulies et al., 2009; Cauda et al., 2010). Similarly, PMC regions are often highlighted by network analyses (van den Heuvel and Sporns, 2013), for example as functional (Buckner et al., 2009; Sepulcre et al., 2012; Zuo et al., 2012), or structural ‘hubs’ (van den Heuvel et al., 2010). Even within modes, the PMC has often been identified as a crucial nexus, with network analyses frequently suggesting that, within the DM, all roads lead to the PCC (Buckner et al., 2008; Fransson and Marrelec, 2008).

Given the wealth of fMRI literature, it would be tempting to start assigning roles to those modes, such as the PMPL, that do not closely correspond with previously characterised functional systems. However, entertaining as it would be to speculate on the cognitive function of modes, this is not something we can do with any great confidence given that “a solid first-order understanding of cortical parcellation remains elusive” (Van Essen and Glasser, 2014). All the results presented here are from analyses of high resolution, surface-based data acquired at low TR—without this high quality surface data it is extremely unlikely that it would be possible to disentangle these modes at all. That being the case, the exact correspondence between these results and those based upon relatively low resolution, volume-based data, used by many of the studies we have cited, is unclear.

**Fig. 8:**
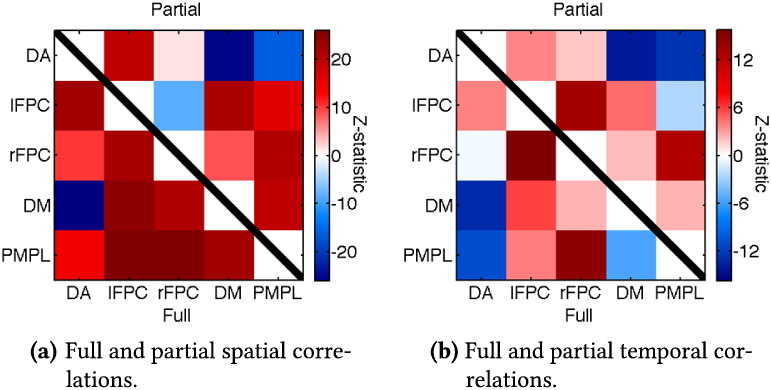
Spatial and temporal interactions between the five PFMs shown in Fig. 7. Firstly, the correlation matrices for each subject's spatial map and time courses are computed and Fisher transformed. A t-test is performed on each element of these correlation matrices—now pooling across subjects—before conversion to the z-statistics we report here. Partial correlations are displayed as the 5 × 5 subset of the full 30 × 30 partial correlation matrices; in other words, they are calculated after orthogonalisation with respect to the twenty five PFMs not highlighted here.

What we can do, however, is use these PFMs to illustrate how our method deals with modes that are both spatially and temporally correlated. The full and partial spatial correlations between the PFMs we have highlighted are shown in Fig. 8a, with the equivalent temporal correlations displayed in Fig. 8b. The fact that there are many non-zero spatial and temporal correlations between modes is evidence in itself that neither sICA nor tICA would be able to accurately infer this exact set of modes' spatial maps and time courses.

The temporal partial correlations between the well established PFMs are consistent with their previous characterisations. The DM strongly anti-correlates with the DA, while the lFPC and rFPC are strongly correlated with each other. Both the DA and DM are partially correlated with the two elements of the FPC, though more weakly. If we now look at the full correlations then the spatial and temporal correlations show a similar pattern of positive and negative correlations between the PFMs.

However, if we look at the interactions of the PMPL we see that there are large, positive spatial correlations with the other four PFMs whereas there is a mixture of positive and negative temporal correlations. The partial correlations show that the PMPL, like the DM, is anti-correlated with the DA; however, it seems to only weakly interact with the DM. What is especially intriguing is that the PMPL does not symmetrically correlate with the lFPC and rFPC. Rather, it seems to weakly anti-correlate with the lFPC but strongly correlate with the rFPC.

The interesting point about this set of interactions is that the spatial correlations are not trivially predictive of the temporal correlations, and vice versa, once again suggesting our approach can identify sets of PFMs with complex spatio-temporal structure.

#### Subject variability

**Fig. 9:**
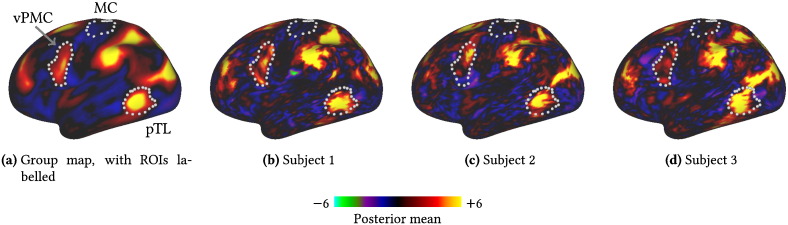
Example subject variants of the dorsal attention PFM spatial map (the group map is also shown also in Fig. 7a). Only the left lateral cortical surface is shown. The borders shown delineate the ROIs used in subsequent analyses. Cortical surface views were generated using Connectome Workbench (www.humanconnectome.org/connectome/connectome-workbench.html).

One important component of our model is the ability to explicitly model differences between subjects. In Fig. 9 we look at three subject-specific variants of the DA.

While the subject variants are unsurprisingly much noisier than the group map, they are all recognisably the same PFM. What is perhaps surprising is the amount of variation in the locations of key spatial features.

For example, the spatial weights in the ventral premotor cortex (vPMC) only appear relatively weakly in the group map. If we look at the three subject variants we can see that this appears strongly in the first two subjects—though in the second subject at a distinctly different position to the peak of the group activation—whereas in the third subject there seem to be no strong spatial weights in that region at all.

Similarly, there is a large amount of variability in the locations of the large spatial weights that encircle visual area MT. In subject 1 the region of large weights in the posterior temporal lobe (pTL)^3^ is completely spatially separated from the other areas, whereas in subject 3 there is a large swathe of strong weights included in the mode linking this region to the rest of the strongly weighted regions within the parietal lobe.

**Fig. 10:**
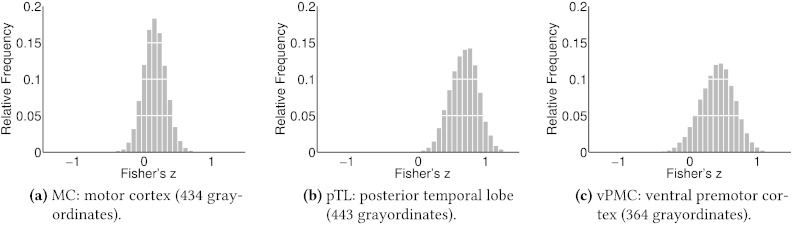
Histograms of the Fisher transformed correlation coefficients between the DA map weights within the three ROIs shown in Fig. 9a. For each ROI, each subject's PFM map weights were extracted and the full subjects-by-subjects correlation matrix, between these sets of weights, was calculated. The elements of the correlation matrices are then Fisher transformed and collected into the histograms shown here. In this case, due to the highly different group mean activities within ROIs, correlation coefficients are taken after the ROIs have been demeaned.

To try to quantify these observations, we carried out an ROI analysis on the subject variants of the DA. The variability across subjects of the spatial activations in the three ROIs shown in Fig. 9 is plotted in Fig. 10. We chose ROIs in the left hemisphere, comprising the region of high activity in the pTL, the weak activity at the group level in the vPMC and a control region, not involved in the DA, situated in the motor cortex (MC).

The ROI in the MC shows a close to zero mean correlation, with a degree of variability which we expect represents a baseline—simply reflecting the relatively small number of grayordinates and the inherently noisy estimates of the individual subject maps. Both the pTL and vPMC show non-zero mean correlations, as expected. However, the pTL exhibits a much larger degree of variability than the MC, and the vPMC is more variable again.

We hypothesise that the high variability, relative to the control, in these regions is mainly attributable to the spatial mismatch between subjects. In other words, in the well aligned subjects the ROIs appear very similar, whereas when the alignment is less accurate the activity within the ROIs barely overlaps at all, leading to very dissimilar ROIs between some subjects. Given this variability, it seems highly unlikely that the region in the vPMC, for example, could be identified reliably without recourse to its strong connectivity to the rest of the DA. This raises several questions about how subject variability influences the appropriate dimensionality of functional parcellations—a point we will return to in the discussion.

**Fig. 11:**
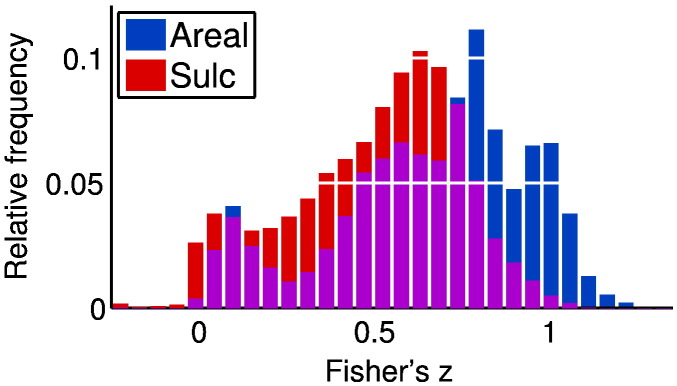
Histograms of the Fisher transformed correlation coefficients between the subject-specific PFM maps under two different registration schemes. MSM_Areal Features_ (Areal) registered subjects using structural and functional information, whereas MSM_Sulc_ (Sulc) used purely structural features. For both sets of results we remove the scores relating to both the global PFM and any artefactual PFMs. Then, for each PFM retained we calculate the correlation coefficients between all pairs of subject specific maps. The histogram pools these scores over all PFMs and subject pairs.

Finally, given that the MSM registration is already informed by functional features, these spatial shifts are somewhat surprising. One possibility is that there is simply a fundamental difference between the measures of similarity we are examining and the metric implied by the particular choice of functional features for registration. To clarify the impact of the functional information on the registration, we re-ran our algorithm on two different random subsets of 50 subjects: one where MSM had been informed by sulcal depth, myelination and subject-specific low dimensional sICA components (MSM_Areal Features_) and the other where MSM had been driven by sulcal depth alone (MSM_Sulc_). We then compared how similar the inferred subject maps were under each of the registration schemes. The results are plotted in Fig. 11.

What is striking is the large shift in the positive mode of the distribution. When subjects are registered with functional information, their subject-specific maps look much more alike, exactly as expected. However, the high variability between subjects, even with the improvements from MSM registration, remains a pressing issue.

#### Comparison with sICA and tICA

As well as comparing the reliability of different decompositions through test–retest reliability, it is also instructive to look at where the similarities and differences lie between approaches. For example, given that the HCP represents a step change in data quality, quantity and preprocessing, it could be that any new results from our decomposition relate to the data, rather than the method.

**Fig. 12:**
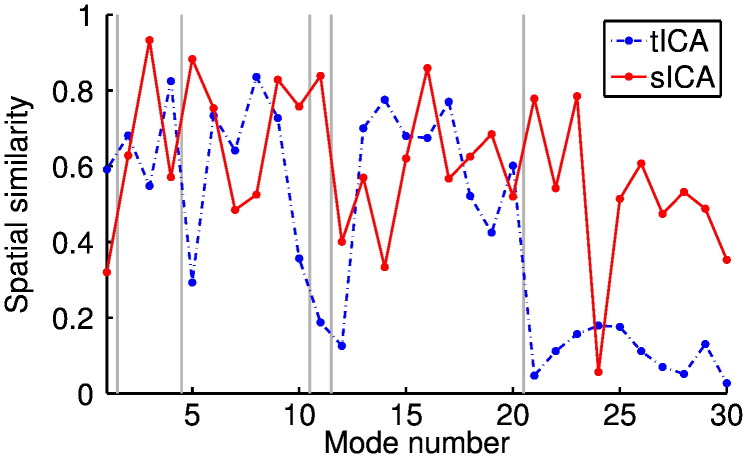
Similarity between the spatial maps identified by our method and both tICA and sICA. Each method was tasked with inferring 30 modes from 209 subjects worth of HCP data. The modes inferred by sICA and tICA were matched to the PFMs from our method and the spatial correlation coefficients between the maps are plotted here. The PFM numbers relate to the numberings in the Supplementary material and are organised into several loose functional categories: a global component (PFM 1); motor (2–4); visual (5–10); auditory (11) and cognitive (12–20). PFMs 21–30 were either harder to classify or eliminated from the model.

To this end, we ran 30 dimensional sICA and tICA on the HCP data and looked at the spatial similarities between the ICA modes and those inferred by our method in the previous section. The results are plotted in Fig. 12 and the ICA maps are shown in the Supplementary material alongside the PFMs they are paired with here. The Supplementary material also contains a similar plot, illustrating the similarity between the sICA and tICA spatial maps.

Most of the PFMs have a fairly close correspondence with a mode identified by one of the ICA methods but crucially neither sICA nor tICA can account for all PFMs; this suggests that our method is identifying a genuinely different decomposition to either ICA approach. That is not to say that we would expect different algorithms to infer wholly unrelated sets of modes. Given that they are all run on the same data and are built on matrix factorisation models, it is natural to expect a fairly large degree of similarity between decompositions—indeed, the opposite would be a major cause for concern in light of the wealth of literature characterising resting state modes.

## Discussion

In this paper, we have introduced a new method for identifying modes from rfMRI. It can disentangle sets of modes with complex spatio-temporal interactions, as well as inferring information about the nature of variations across subjects. Our results suggest that our method is at least as robust as current popular methods, despite fitting a more complex model to the data. Furthermore, the non-trivial temporal correlation structures we identify, in agreement with existing results from both the task and resting state literature, give us confidence that we can identify modes with the kind of dynamics that will be amenable to non-stationary functional connectivity network analyses.

### Nodes: modes or parcels?

Our model is explicitly based around the concept of modes, in that we deliberately place few hard restrictions on the spatial structure of the components we infer. This naturally leads to identification of components that, due to their large spatial extent and prominent anti-correlations, often superficially resemble previous results from tICA analyses. These PFMs are clearly interesting in their own right; however, given the recent interest in non-stationary functional network analyses, high-dimensional parcellations, based around spatially compact and positively correlated regions, have come to the fore.

Ultimately, the choice of whether to analyse modes or parcels depends on, to varying extents, the research question, data properties and personal preferences. Here, we briefly discuss the implications of our results for this choice.

#### Network analyses

From a network analysis perspective, both parcels and modes are fundamentally looking for the same thing—ways to simplify the representation of voxelwise rfMRI connectivity with as little information loss as possible. This naturally leads to the definition of regions as groups of voxels that share a common time course, a concept which can be formalised mathematically as a matrix factorisation approach (Eq. (1)).

While there is a common mathematical grounding, parcels have come to the fore for several reasons. Firstly, network analyses of parcel time courses are arguably more interpretable; the interactions between distinct brain regions are a conceptually simpler construct than those between gross cortical ensembles that potentially overlap and contain anti-correlations. Secondly, if we could identify a high dimensional parcellation reliably, then mode structures should naturally fall out of any subsequent network analysis—the concern is that the spatial scale of modes leads to loss of useful information. Indeed, the characterisation of temporal functional modes using tICA was explicitly based around this notion: a high dimensional set of parcels was identified before these were combined into a temporally independent set of modes. Note that this is the stICA approach we investigate in our simulations.

It is not unreasonable to assume that the higher the dimensionality of the parcellation the richer the description of the data. However, the crux of the matter is that the parcellation has to be both meaningful and reliable. Therefore, in light of subject variability and noisy data, it is imperative that the suitability of any parcellation comes under rigorous scrutiny; if this is improperly defined all subsequent network analyses will be rendered meaningless.

#### Implications of subject variability

The results we have presented from our modelling of subject variability lead us to believe that spatial mismatches are one of the biggest, if not the biggest, sources of currently observable differences between subjects. These mismatches presumably reflect some combination of misalignments, either arising from cases where the registration is not able to make functional regions perfectly coincident, or, alternatively, cases where registration could not possibly work as the areal topology is genuinely different between subjects.

For example, given the variability in spatial location of the region in the vPMC that correlates with the rest of the DA, we do not believe that a group-level parcellation at this spatial scale^4^ would be robust enough to enforce functional correspondence across subjects.

The only reason our model can reliably find this region, despite its variable spatial location, is that it consistently correlates with the much larger spatial regions of the DA in the parietal and temporal lobes. Crucially, the mode as a whole is large enough to be robust to variations in functional localisation across subjects. However, this does not hold if the distinct regions that form the mode—in other words, the parcels we would hope to uncover from a higher dimensional analysis—are considered separately from each other.

Again, this is a particular surprise given we are already using functional registration—in this case using MSM, though several methods have been proposed (Conroy et al., 2013; Li and Fan, 2014)—and as we show in Fig. 11 it is working well. Our model for subject variability is not explicitly designed to capture spatial shifts, though the fact that it can correct for them to some extent is particularly encouraging. Our view is that the relationship between models like ours and functional registration algorithms should be a symbiotic one, where better characterisation of functional data can be used to increase the accuracy of the registrations, and improved registration allows functional information to be spatially localised with increasing specificity.

#### Spatial templates

In their discussion of the work of Saygin et al. (2012), which used connectivity rather than spatial information to predict functional responses, Jbabdi and Behrens noted that “[b]y mapping onto a purely spatial template, we lose a great deal of detail that is present in individual responses, and we are left to interpret only the spatial peaks that are consistent across subjects” (Jbabdi and Behrens, 2012). Hopefully, what we have shown here is that provided spatially consistent peaks do exist, it is then possible to recover some of the information about individual responses. An interpretation of the way our model for subject variability is able to infer this information is that it, very naïvely, uses the strong connectivities between the different regions that form a PFM to correct for spatial variability.

However, while our model should ameliorate these problems to some extent, this is by no means addressing the pivotal issues head-on. That a voxel or region should be intrinsically defined by its connections seems an eminently sensible approach; how this transition from a spatial to a connectivity-based template is achieved in practice is an extensive area for future research.

Finally, we believe that it is this need for spatially consistent peaks to exist that severely restricts the minimum spatial extents of modes. Our finding from the test–retest reliability scores, that around 20 PFMs are reliably identified, does seem to agree with previous results. For example, in their analysis, Yeo et al. examined the stability of non-overlapping parcels identified with a clustering approach (Yeo et al., 2011). They found that there were peaks in the stability of the clustering when 7, 10, 12 or 17 parcels were identified—a result that is perhaps challenging to reconcile with the current tendency to use high-dimensional parcellations. The improvements in alignment offered by MSM and our relaxation of some of the spatial assumptions they made, especially allowing overlap, leads us to believe that our method would naturally find more PFMs. However, we only find a few more PFMs; our interpretation of both these findings is that modes have to cover a surprisingly large spatial area in order to be robust to subject variability.

### Computational considerations

Crucially, our algorithm operates on the full voxelwise data. Having to work in a reduced dimensionality subspace, for example a small set of PCA components, is a fundamental limit for several algorithms—including the ICA approaches we have tested here. While post-hoc techniques, like dual regression, can go some way towards alleviating this, there seem to be advantages to working with the full data.

However, for projects like the HCP with very large cohorts, simultaneously working with hundreds of runs in this way poses a not insignificant computational challenge. For example, simply loading the data from the 209 subjects we have analysed in this paper requires nearly 700 GB of memory. Similarly, our algorithm took almost a week to run on this data, typically utilising twelve cores of the machine. By way of comparison, the incremental group PCA, used as a first step for the ICA approaches, still takes several days to run on the same data, albeit with much smaller memory requirements (Smith et al., 2014).

These types of computational issues will only become more pressing as these large projects become the norm—the HCP alone will ultimately contain six times as many subjects as analysed here. In a sense, the approach to these computational issues is the fundamental difference between our approach and MSDL. Our spatial prior still allows for subject modelling, but by choosing not to impose a spatial smoothness constraint the computational complexity of our algorithm is reduced, thereby allowing the analysis of hundreds of subjects simultaneously. As discussed earlier, similar simplifications were made for the HRF modelling too. We are not trying to claim that ours is the optimal solution, as the MSDL cost functions clearly have their own advantages. However, the trade-off between model complexity and data throughput is likely to come under increasing scrutiny as the amount of available data swells.

### Bayesian modelling

One of the major driving forces behind some of our choices when developing our probabilistic model was ensuring that the model remained amenable to Bayesian inference; for example, the variational approximation introduces deviations from the ‘true’ posterior, but is extremely computationally efficient. Given these trade-offs, it would be reasonable to ask whether the benefits of a fully Bayesian model really do outweigh the costs, especially as in the results presented here we have tended to focus on very simple summary statistics of our posterior distribution—often simply the posterior mean of the parameters in question.

We firmly believe that full Bayesian modelling is a useful approach, for several reasons. Firstly, by not only learning parameters, but also their associated uncertainty, the overall inference improves.

Secondly, the posterior distribution is an enormously rich description of the data. While this is challenging to visualise, we expect that this will contain genuinely interesting information. Furthermore, this is information that could be incorporated into any subsequent network analysis.

Finally, and perhaps most importantly, the Bayesian approach gives a principled framework in which to build and compare models. We have made several distinct modelling choices—looking for modes rather than parcels or considering a canonical linear HRF, for example. However, these are choices; it would be straightforward to alter the model to reflect a different set of assumptions. What we have demonstrated here is that it is possible to build ambitious models, tailored to fMRI data, that are computationally feasible even for huge numbers of subjects.

## Algorithm

We refer to our approach/software as PROFUMO (PRObabilistic FUnctional MOdes). Our hope is that a future version of PROFUMO can be made publicly available as part of FSL.

## Footnotes

1 In other words, the ratio of the BOLD signal variance to the noise variance is 0.1.

2 It therefore bears a passing resemblance to a parcel identified by Power et al. (2011), though as their parcels could not spatially overlap this is certainly ambiguous.

3 At the group level at least, these weights seem to be focused around a bridging gyrus linking the posterior middle temporal gyrus and posterior inferior temporal gyrus, with excursions into both of these temporal gyri.

4 This ROI contains around 450 voxels, which would lead to a spatial atlas of around 200 regions if hemispheres were treated independently.

## References

[bb0270] Abraham A., Dohmatob E., Thirion B., Samaras D., Varoquaux G., Mori K., Sakuma I., Sato Y., Barillot C., Navab N. (2013). Extracting brain regions from rest fMRI with total-variation constrained dictionary learning. Medical Image Computing and Computer-Assisted Intervention—MICCAI 2013.

[bb0285] Aguirre G., Zarahn E., D'Esposito M. (1998). The variability of human, BOLD hemodynamic responses. NeuroImage.

[bb0145] Allen E.A., Damaraju E., Plis S.M., Erhardt E.B., Eichele T., Calhoun V.D. (2014). Tracking whole-brain connectivity dynamics in the resting state. Cereb. Cortex.

[bb0245] Allen G.I., Grosenick L., Taylor J. (2014). A generalized least-square matrix decomposition. J. Am. Stat. Assoc..

[bb0150] Baker A.P., Brookes M.J., Rezek I.A., Smith S.M., Behrens T.E.J., Probert Smith P.J., Woolrich M.W. (2014). Fast transient networks in spontaneous human brain activity. eLife.

[bb0205] Beckmann C.F., DeLuca M., Devlin J.T., Smith S.M. (2005). Investigations into resting-state connectivity using independent component analysis. Phil. Trans. R. Soc. B.

[bb0335] Beckmann C.F., Mackay C., Filippini N., Smith S.M. (2009). Group comparison of resting-state FMRI data using multi-subject ICA and dual regression. Organization for Human Brain Mapping.

[bb0130] Biswal B., Zerrin Yetkin F., Haughton V.M., Hyde J.S. (1995). Functional connectivity in the motor cortex of resting human brain using echo-planar MRI. Magn. Reson. Med..

[bb0025] Buckner R.L., Andrews-Hanna J.R., Schacter D.L. (2008). The brain's default network: anatomy, function, and relevance to disease. Ann. N. Y. Acad. Sci..

[bb0075] Buckner R.L. (2009). Cortical hubs revealed by intrinsic functional connectivity: mapping, assessment of stability, and relation to Alzheimer's disease. J. Neurosci..

[bb0065] Cauda F. (2010). Functional connectivity of the posteromedial cortex. PLoS ONE.

[bb0255] Cohen A.L., Fair D.A., Dosenbach N.U.F., Miezin F.M., Dierker D., Van Essen D.C., Schlaggar B.L., Petersen S.E. (2008). Defining functional areas in individual human brains using resting functional connectivity MRI. NeuroImage.

[bb0040] Cole M.W. (2013). Multi-task connectivity reveals flexible hubs for adaptive task control. Nat. Neurosci..

[bb0105] Conroy B.R. (2013). Inter-subject alignment of human cortical anatomy using functional connectivity. NeuroImage.

[bb0015] Corbetta M., Shulman G.L. (2002). Control of goal-directed and stimulus-driven attention in the brain. Nat. Rev. Neurosci..

[bb0190] Craddock R.C., James G., Holtzheimer P.E., Hu X.P., Mayberg H.S. (2012). A whole brain fMRI atlas generated via spatially constrained spectral clustering. Hum. Brain Mapp..

[bb0155] Cribben I., Haraldsdottir R., Atlas L.Y., Wager T.D., Lindquist M.A. (2012). Dynamic connectivity regression: determining state-related changes in brain connectivity. NeuroImage.

[bb0210] Damoiseaux J.S., Rombouts S.A.R.B., Barkhof F., Scheltens P., Stam C.J., Smith S.M., Beckmann C.F. (2006). Consistent resting-state networks across healthy subjects. Proc. Natl. Acad. Sci..

[bb0305] Deco G., Jirsa V.K., McIntosh A.R. (2011). Emerging concepts for the dynamical organization of resting-state activity in the brain. Nat. Rev. Neurosci..

[bb0260] Eavani H., Satterthwaite T.D., Gur R.E., Gur R.C., Davatzikos C. (2013). Unsupervised learning of functional network dynamics in resting state fMRI. Information Processing in Medical Imaging.

[bb0225] Erhardt E.B., Rachakonda S., Bedrick E.J., Allen E.A., Adal T., Calhoun V.D. (2011). Comparison of multi-subject ICA methods for analysis of fMRI data. Hum. Brain Mapp..

[bb0215] Filippini N., MacIntosh B.J., Hough M.G., Goodwin G.M., Frisoni G.B., Smith S.M., Matthews P.M., Beckmann C.F., Mackay C.E. (2009). Distinct patterns of brain activity in young carriers of the APOE-_ε_4 allele. Proc. Natl. Acad. Sci..

[bb0175] Fornito A., Zalesky A., Breakspear M. (2013). Graph analysis of the human connectome: promise, progress, and pitfalls. NeuroImage.

[bb0095] Fransson P., Marrelec G. (2008). The precuneus/posterior cingulate cortex plays a pivotal role in the default mode network: evidence from a partial correlation network analysis. NeuroImage.

[bb0195] Friston K.J. (1998). Modes or models: a critique on independent component analysis for fMRI. Trends Cogn. Sci..

[bb0325] Friston K.J., Josephs O., Rees G., Turner R. (1998). Nonlinear event-related responses in fMRI. Magn. Reson. Med..

[bb0055] Glasser M.F., Van Essen D.C. (2011). Mapping human cortical areas in vivo based on myelin content as revealed by T1- and T2-weighted MRI. J. Neurosci..

[bb0370] Griffanti L. (2014). ICA-based artefact removal and accelerated fMRI acquisition for improved resting state network imaging. NeuroImage.

[bb0345] Groppe D.M., Makeig S., Kutas M. (2009). Identifying reliable independent components via split-half comparisons. NeuroImage.

[bb0010] Groves A.R. (2011). Linked independent component analysis for multimodal data fusion. NeuroImage.

[bb0290] Handwerker D.A., Ollinger J.M., D'Esposito M. (2004). Variation of BOLD hemodynamic responses across subjects and brain regions and their effects on statistical analyses. NeuroImage.

[bb0330] Hyvärinen A. (1999). Fast and robust fixed-point algorithms for independent component analysis. IEEE Trans. Neural Netw..

[bb0230] Hyvärinen A. (2013). Independent component analysis: recent advances. Philos. Trans. R. Soc. A Math. Phys. Eng. Sci..

[bb0235] Hyvärinen A., Hoyer P.O. (2000). Emergence of phase- and shift-invariant features by decomposition of natural images into independent feature subspaces. Neural Comput..

[bb0240] Hyvärinen A., Hoyer P.O., Inki M. (2001). Topographic independent component analysis. Neural Comput..

[bb0120] Jbabdi S., Behrens T.E.J. (2012). Specialization: the connections have it. Nat. Neurosci..

[bb0135] Kiviniemi V., Kantola J.-H., Jauhiainen J., Hyvärinen A., Tervonen O. (2003). Independent component analysis of nondeterministic fMRI signal sources. NeuroImage.

[bb0165] Kiviniemi V., Starck T., Remes J., Long X., Nikkinen J., Haapea M., Veijola J., Moilanen I., Isohanni M., Zang Y.-F., Tervonen O. (2009). Functional segmentation of the brain cortex using high model order group PICA. Hum. Brain Mapp..

[bb0295] Kriegeskorte N., Cusack R., Bandettini P. (2010). How does an fMRI voxel sample the neuronal activity pattern: compact-kernel or complex spatiotemporal filter?. NeuroImage.

[bb0340] Kuhn H.W. (1955). The Hungarian method for the assignment problem. Nav. Res. Logist. Q..

[bb0250] Lee J.-H., Hashimoto R., Wible C.G., Yoo S.-S. (2011). Investigation of spectrally coherent resting-state networks using non-negative matrix factorization for functional MRI data. Int. J. Imaging Syst. Technol..

[bb0110] Li H., Fan Y. (2014). Spatial alignment of human cortex by matching hierarchical patterns of functional connectivity. Biomedical Imaging (ISBI), 2014 IEEE 11th International Symposium on.

[bb0060] Margulies D.S. (2009). Precuneus shares intrinsic functional architecture in humans and monkeys. Proc. Natl. Acad. Sci..

[bb0350] McKeown M.J., Makeig S., Brown G.G., Jung T.-P., Kindermann S.S., Kindermann R.S., Bell A.J., Sejnowski T.J. (1998). Analysis of fMRI data by blind separation into independent spatial components. Hum. Brain Mapp..

[bb0310] Niazy R.K., Xie J., Miller K.L., Beckmann C.F., Smith S.M., Van Someren E.J.W., Van Der Werf Y.D., Roelfsema P.R., Mansvelder H.D., Lopes da Silva F.H. (2011). Spectral characteristics of resting state networks. Progress in Brain Research: Slow Brain Oscillations of Sleep, Resting State and Vigilance.

[bb0045] Power J. (2011). Functional network organization of the human brain. Neuron.

[bb0020] Raichle M.E., MacLeod A.M., Snyder A.Z., Powers W.J., Gusnard D.A., Shulman G.L. (2001). A default mode of brain function. Proc. Natl. Acad. Sci..

[bb0005] Robinson E.C., Gee J.C. (2013). Multimodal surface matching: fast and generalisable cortical registration using discrete optimisation. Information Processing in Medical Imaging.

[bb0375] Robinson E.C. (2014). MSM: a new flexible framework for multimodal surface matching. NeuroImage.

[bb0180] Rubinov M., Sporns O. (2010). Complex network measures of brain connectivity: uses and interpretations. NeuroImage.

[bb0365] Salimi-Khorshidi G. (2014). Automatic denoising of functional MRI data: combining independent component analysis and hierarchical fusion of classifiers. NeuroImage.

[bb0115] Saygin Z.M. (2012). Anatomical connectivity patterns predict face selectivity in the fusiform gyrus. Nat. Neurosci..

[bb0160] Seghier M.L., Friston K.J. (2013). Network discovery with large DCMs. NeuroImage.

[bb0080] Sepulcre J. (2012). Stepwise connectivity of the modal cortex reveals the multimodal organization of the human brain. J. Neurosci..

[bb0050] Sereno M.I. (2013). Mapping the human cortical surface by combining quantitative T1 with retinotopy. Cereb. Cortex.

[bb0300] Siegel M., Donner T.H., Engel A.K. (2012). Spectral fingerprints of large-scale neuronal interactions. Nat. Rev. Neurosci..

[bb0140] Smith S.M., Fox P.T., Miller K.L., Glahn D.C., Fox P.M., Mackay C.E., Filippini N., Watkins K.E., Toro R., Laird A.R., Beckmann C.F. (2009). Correspondence of the brain's functional architecture during activation and rest. Proc. Natl. Acad. Sci..

[bb0200] Smith S.M., Miller K.L., Moeller S., Xu J., Auerbach E.J., Woolrich M.W., Beckmann C.F., Jenkinson M., Andersson J., Glasser M.F., Van Essen D.C., Feinberg D.A., Yacoub E.S., Ugurbil K. (2012). Temporally-independent functional modes of spontaneous brain activity. Proc. Natl. Acad. Sci..

[bb0170] Smith S.M., Vidaurre D., Beckmann C.F., Glasser M.F., Jenkinson M., Miller K.L., Nichols T.E., Robinson E.C., Salimi-Khorshidi G., Woolrich M.W., Barch D.M. (2013). Functional connectomics from resting-state fMRI. Trends Cogn. Sci..

[bb0360] Smith S.M. (2013). Resting-state fMRI in the human connectome project. NeuroImage.

[bb0125] Smith S.M. (2014). Group-PCA for very large fMRI datasets. NeuroImage.

[bb0035] Spreng R.N. (2010). Default network activity, coupled with the frontoparietal control network, supports goal-directed cognition. NeuroImage.

[bb0280] Titsias M., Lzaro-Gredilla M., Shawe-Taylor J., Zemel R., Bartlett P., Pereira F., Weinberger K. (2011). Spike and slab variational inference for multi-task and multiple kernel learning.

[bb0070] van den Heuvel M.P., Sporns O. (2013). Network hubs in the human brain. Trends Cogn. Sci..

[bb0090] van den Heuvel M.P. (2010). Aberrant frontal and temporal complex network structure in schizophrenia: a graph theoretical analysis. J. Neurosci..

[bb0100] Van Essen D.C., Glasser M.F. (2014). In vivo architectonics: a cortico-centric perspective. NeuroImage.

[bb0355] Van Essen D. (2012). The Human Connectome Project: a data acquisition perspective. NeuroImage.

[bb0275] Van Essen D.C., Smith S.M., Barch D.M., Behrens T.E., Yacoub E., Ugurbil K. (2013). The WU-Minn Human Connectome Project: an overview. NeuroImage.

[bb0220] Varoquaux G., Sadaghiani S., Pinel P., Kleinschmidt A., Poline J., Thirion B. (2010). A group model for stable multi-subject ICA on fMRI datasets. NeuroImage.

[bb0265] Varoquaux G., Gramfort A., Pedregosa F., Michel V., Thirion B., Szkely G., Hahn H.K. (2011). Multi-subject dictionary learning to segment an atlas of brain spontaneous activity. Information Processing in Medical Imaging.

[bb0030] Vincent J.L. (2008). Evidence for a frontoparietal control system revealed by intrinsic functional connectivity. J. Neurophysiol..

[bb0315] Winn J., Bishop C.M., Jaakkola T. (2005). Variational message passing.

[bb0320] Woolrich M.W., Behrens T.E., Smith S.M. (2004). Constrained linear basis sets for HRF modelling using variational Bayes. NeuroImage.

[bb0185] Yeo T.B.T., Krienen F.M., Sepulcre J., Sabuncu M.R., Lashkari D., Hollinshead M., Roffman J.L., Smoller J.W., Zllei L., Polimeni J.R., Fischl B., Liu H., Buckner R.L. (2011). The organization of the human cerebral cortex estimated by intrinsic functional connectivity. J. Neurophysiol..

[bb0085] Zuo X.-N. (2012). Network centrality in the human functional connectome. Cereb. Cortex.

