# Supplementary material for: Large-scale Probabilistic Functional Modes from resting state fMRI
Source: Neuroimage. 2015 Apr 1;109:217–31. doi: 10.1016/j.neuroimage.2015.01.013 (PMC4349633; doi:10.1016/j.neuroimage.2015.01.013)
Supplement: Supplementary file 1 — Supplementary material. [file mmc1.pdf]

Large-scale Probabilistic Functional Modes from resting state fMRI

Supplementary Material

Samuel J. Harrison, Mark W. Woolrich, Emma C. Robinson, Matthew F. Glasser, Christian F. Beckmann, Mark Jenkinson and Stephen M. Smith

Contents

1 Variational Bayesian Update Rules **2**
1.1 Theory . . . 2
1.2 Notation . . . 2
1.3 Model . . . 3
1.4 Spatial Maps . . . 3
1.4.1 Subject Maps . . . 3
1.4.2 Group Mixture Weights . . . 4
1.4.3 Group Precisions . . . 4
1.4.4 Group Means . . . 5
1.4.5 Group Mean Precisions . . . 5
1.5 Time Courses . . . 6
1.5.1 Precision . . . 6
1.6 Noise . . . 6
1.6.1 Precision . . . 6
1.6.2 Mean . . . 7

2 Algorithm **7**
2.1 Initialisation . . . 7
2.2 Parameters . . . 8

3 Simulated Data **8**
3.1 Data generation . . . 8
3.1.1 Spatial atlas . . . 8
3.1.2 Mode weights . . . 9
3.1.3 Neural time courses . . . 9
3.1.4 BOLD signal . . . 10
3.1.5 Noise . . . 10
3.1.6 Examples . . . 10
3.2 Dual regression . . . 10
3.3 Test-retest reliability versus ground truth accuracy . . . 11
3.4 Simulation results with mismatched mode numbers . . . 11
3.5 Performance of PCA . . . 11

4 HCP Results **25**

5 Theoretical comparison of PFMs and ICA **57**
5.1 Definitions . . . 57
5.1.1 Independence . . . 57
5.1.2 Correlation and covariance . . . 57
5.1.3 Results from finite samples . . . 58
5.2 ICA model . . . 58
5.2.1 Correlations . . . 59
5.2.2 Whitening . . . 59
5.2.3 ICA and fMRI . . . 60
5.3 PFM model . . . 60

1 Variational Bayesian Update Rules

In this section, we provide the update rules for our model. We briefly describe the theory and notation before providing the update rules themselves.

1.1 Theory

Variational Bayesian (VB) inference proceeds by optimising an approximation to the full posterior distribution. The motivation for this can be seen from the equation below

$$\begin{aligned}\ln p(\mathcal{D}) &= \ln \int p(\mathcal{D}, \boldsymbol{\Theta})\ \mathrm{d}\boldsymbol{\Theta} \\ &= \ln \int q(\boldsymbol{\Theta}) \frac{p(\mathcal{D}, \boldsymbol{\Theta})}{q(\boldsymbol{\Theta})}\ \mathrm{d}\boldsymbol{\Theta} \\ &\geq \int q(\boldsymbol{\Theta}) \ln \left(\frac{p(\mathcal{D}, \boldsymbol{\Theta})}{q(\boldsymbol{\Theta})} \right) \mathrm{d}\boldsymbol{\Theta}\end{aligned}$$

The final form is the free energy, which forms a rigorous lower bound on the true marginal likelihood for any approximating distribution $q(\boldsymbol{\Theta})$. The difference between the true marginal likelihood and the free energy is simply the KL divergence between $q(\boldsymbol{\Theta})$ and the true posterior $p(\boldsymbol{\Theta}|\mathcal{D})$. Therefore, by maximising the free energy we minimise the difference between $q(\boldsymbol{\Theta})$ and the true posterior.

The aim is to choose a form for $q(\boldsymbol{\Theta})$ that allows a close approximation of the true posterior, while being simple enough allow the evaluation and optimisation of the free energy. Here we use the mean field approximation i.e. we factorise $q(\boldsymbol{\Theta})$ over convenient groups of variables

$$q(\boldsymbol{\Theta}) = \prod_{\theta \in \boldsymbol{\Theta}} q(\theta)$$

Finally, by appropriate use of conjugate exponential priors, the update rules that govern the optimisation of $q(\boldsymbol{\Theta})$ have a simple form.

1.2 Notation

Prior distributions are denoted $p(\boldsymbol{A})$ while the approximate posteriors will be designated $q(\boldsymbol{A})$. For convenience we will use the following notation to denote the expectation of a variable with respect to its approximate posterior:

$$\langle \boldsymbol{A} \rangle = \int \boldsymbol{A}\, q(\boldsymbol{A})\ \mathrm{d}\boldsymbol{A}$$

We will use subscripts to index elements in matrices, so $\boldsymbol{A}_{mt}$ is the activity of mode m at time t. If there is only one subscript for a matrix then this represents the extraction of a particular row or column from the matrix. For example, $\boldsymbol{A}_m$ extracts

the full time course from mode m. Whether a row or column is to be extracted should be obvious from the context. We will use a tilde to denote extraction of all elements except the current index. Therefore, $\boldsymbol{A}_{\widetilde{m}}$ represents the matrix formed from the time courses of all modes except mode m.

1.3 Model

The model is sketched in outline in the main paper. For convenience we provide a graphical model in figure S1.

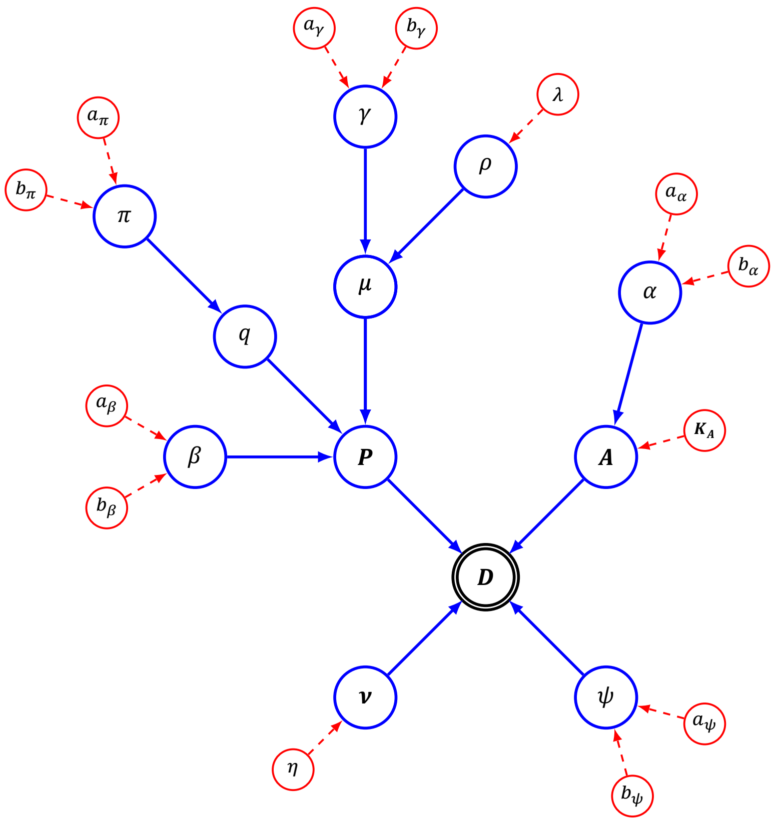

Supplementary figure S1: Graphical representation of the full model structure. Variables are shown in the blue circles and the dependencies are depicted as the arrows joining them. Prior parameters are shown in red.

1.4 Spatial Maps

1.4.1 Subject Maps

Note we have renamed the prior variance of the Gaussian mixture component compared to the main body of the paper. This is purely notational.

$$
\begin{aligned}
p\left(\boldsymbol{P}_{vm}^{(s)} | q_{vm}^{(s)} = 1\right) &= \mathcal{N}\left(\boldsymbol{P}_{vm}^{(s)} | \mu_{vm}, \beta_{vm}^{-1}\right) \\
p\left(\boldsymbol{P}_{vm}^{(s)} | q_{vm}^{(s)} = 0\right) &= \delta\left(\boldsymbol{P}_{vm}^{(s)}\right) \\
p\left(q_{vm}^{(s)}\right) &= \left(\pi_{vm}\right)^{q_{vm}^{(s)}} \left(1 - \pi_{vm}\right)^{1-q_{vm}^{(s)}}
\end{aligned}
$$

$$
\begin{aligned}
q\big(\boldsymbol{P}_{vm}^{(s)} | q_{vm}^{(s)} = 1\big) &= \mathcal{N}\big(\boldsymbol{P}_{vm}^{(s)} | m_{\boldsymbol{P}_{vm}^{(s)}}, \sigma^2_{\boldsymbol{P}_{vm}^{(s)}}\big) \\
q\big(\boldsymbol{P}_{vm}^{(s)} | q_{vm}^{(s)} = 0\big) &= \delta\big(\boldsymbol{P}_{vm}^{(s)}\big) \\
q\big(q_{vm}^{(s)}\big) &= (\phi_{q_{vm}^{(s)}})^{q_{vm}^{(s)}} (1 - \phi_{q_{vm}^{(s)}})^{1-q_{vm}^{(s)}}
\end{aligned}
$$

$$
\sigma_{\boldsymbol{P}_{vm}^{(s)}} = \left(\langle \beta_{vm} \rangle + \sum_{r=1}^{R(s)} \langle \psi^{(sr)} \rangle \langle \boldsymbol{A}_m^{(sr)} (\boldsymbol{A}_m^{(sr)})^{\mathrm{T}} \rangle \right)^{-\frac{1}{2}}
$$

$$
\begin{aligned}
m_{\boldsymbol{P}_{vm}^{(s)}} = \sigma^2_{\boldsymbol{P}_{vm}^{(s)}} \Bigg(& \sum_{r=1}^{R(s)} \langle \psi^{(sr)} \rangle \big(\boldsymbol{D}_v^{(sr)} - \langle \boldsymbol{\nu}_v^{(sr)} \rangle \big) \langle \boldsymbol{A}_m^{(sr)} \rangle^{\mathrm{T}} \\
& - \sum_{r=1}^{R(s)} \langle \psi^{(sr)} \rangle \langle \boldsymbol{P}_{\widetilde{m}}^{(s)} \rangle \langle \boldsymbol{A}_{\widetilde{m}}^{(sr)} (\boldsymbol{A}^{(sr)})^{\mathrm{T}} \rangle + \langle \beta_{vm} \rangle \langle \mu_{vm} \rangle \Bigg)
\end{aligned}
$$

$$
\begin{aligned}
\ln(\phi^{(0)}_{q_{vm}^{(s)}}) &= \big\langle \ln(1 - \pi_{vm}) \big\rangle \\
\ln(\phi^{(1)}_{q_{vm}^{(s)}}) &= \big\langle \ln(\pi_{vm}) \big\rangle + \frac{1}{2} \big\langle \ln(\beta_{vm}) \big\rangle - \frac{1}{2} \langle \beta_{vm} \rangle \langle \mu^2_{vm} \rangle \\
& \quad - \frac{1}{2} \ln(\sigma^{-2}_{\boldsymbol{P}_{vm}^{(s)}}) + \frac{1}{2} \sigma^{-2}_{\boldsymbol{P}_{vm}^{(s)}} m^2_{\boldsymbol{P}_{vm}^{(s)}} \\
\phi_{q_{vm}^{(s)}} &= \frac{\phi^{(1)}_{q_{vm}^{(s)}}}{\phi^{(0)}_{q_{vm}^{(s)}} + \phi^{(1)}_{q_{vm}^{(s)}}}
\end{aligned}
$$

1.4.2 Group Mixture Weights

$$
p(\pi_{vm}) = \beta(a_{\pi_{vm}}, b_{\pi_{vm}})
$$

$$
p(\pi_{vm}) = \beta(c_{\pi_{vm}}, d_{\pi_{vm}})
$$

$$
c_{\pi_{vm}} = a_{\pi_{vm}} + \sum_{s=1}^{S} \langle q_{vm}^{(s)} \rangle
$$

$$
d_{\pi_{vm}} = b_{\pi_{vm}} + S - \sum_{s=1}^{S} \langle q_{vm}^{(s)} \rangle
$$

1.4.3 Group Precisions

$$
p(\beta_{vm}) = \Gamma(a_{\beta_{vm}}, b_{\beta_{vm}})
$$

$$
q(\beta_{vm}) = \Gamma(c_{\beta_{vm}}, d_{\beta_{vm}})
$$

$$c_{\beta_{vm}} = a_{\beta_{vm}} + \frac{1}{2}\sum_{s=1}^{S}\langle q_{vm}^{(s)}\rangle$$

$$d_{\beta_{vm}} = b_{\beta_{vm}} + \frac{1}{2}\sum_{s=1}^{S}\langle q_{vm}^{(s)}\rangle\Big(\langle(\boldsymbol{P}_{vm}^{(s)})^2|q_{vm}^{(s)} = 1\rangle + \langle(\boldsymbol{\mu}_{vm})^2\rangle - 2\langle\boldsymbol{P}_{vm}^{(s)}|q_{vm}^{(s)} = 1\rangle\langle\boldsymbol{\mu}_{vm}\rangle\Big)$$

1.4.4 Group Means

$$\begin{aligned} p(\mu_{vm}|\rho_{vm} = 1) &= \mathcal{N}(\mu_{vm}|0, \gamma_m^{-1}) \\ p(\mu_{vm}|\rho_{vm} = 0) &= \delta(\mu_{vm}) \\ p(\rho_{vm}) &= (\lambda)^{\rho_{vm}}(1-\lambda)^{1-\rho_{vm}} \end{aligned}$$

$$\begin{aligned} q(\mu_{vm}|\rho_{vm} = 1) &= \mathcal{N}(\mu_{vm}|m_{\mu_{vm}}, \sigma_{\mu_{vm}}^2) \\ q(\mu_{vm}|\rho_{vm} = 0) &= \delta(\mu_{vm}) \\ q(\rho_{vm}) &= (\phi_{\rho_{vm}})^{\rho_{vm}}(1-\phi_{\rho_{vm}})^{1-\rho_{vm}} \end{aligned}$$

$$\sigma_{\mu_{vm}} = \Big(\langle\gamma_m\rangle + \langle\beta_{vm}\rangle\sum_{s=1}^{S}\langle q_{vm}^{(s)}\rangle\Big)^{-\frac{1}{2}}$$

$$m_{\mu_{vm}} = \sigma_{\mu_{vm}}^2\langle\beta_{vm}\rangle\sum_{s=1}^{S}\langle\boldsymbol{P}_{vm}^{(s)}|q_{vm}^{(s)} = 1\rangle\langle q_{vm}^{(s)}\rangle$$

$$\begin{aligned} \ln(\phi_{\rho_{vm}}^{(0)}) &= \ln(1-\lambda) \\ \ln(\phi_{\rho_{vm}}^{(1)}) &= \ln(\lambda) + \frac{1}{2}\langle\ln(\gamma_m)\rangle - \frac{1}{2}\ln(\sigma_{\mu_{vm}}^{-2}) + \frac{1}{2}\sigma_{\mu_{vm}}^{-2}m_{\mu_{vm}}^2 \\ \phi_{\rho_{vm}} &= \frac{\phi_{\rho_{vm}}^{(1)}}{\phi_{\rho_{vm}}^{(0)} + \phi_{\rho_{vm}}^{(1)}} \end{aligned}$$

1.4.5 Group Mean Precisions

$$p(\gamma_m) = \Gamma(a_{\gamma_m}, b_{\gamma_m})$$

$$q(\gamma_m) = \Gamma(c_{\gamma_m}, d_{\gamma_m})$$

$$c_{\gamma_m} = a_{\gamma_m} + \frac{1}{2}\sum_{v=1}^{V}\langle\rho_{vm}\rangle$$

$$d_{\gamma_m} = b_{\gamma_m} + \frac{1}{2}\sum_{v=1}^{V}\langle(\mu_{vm})^2|\rho_{vm} = 1\rangle\langle\rho_{vm}\rangle$$

1.5 Time Courses

$$p\big(\text{vec}(\boldsymbol{A}^{(sr)})\big) = \mathcal{N}(\boldsymbol{0}, \alpha^{-1}\boldsymbol{I}_M \otimes \boldsymbol{K}_{\boldsymbol{A}})$$

$$q\big(\text{vec}(\boldsymbol{A}^{(sr)})\big) = \mathcal{N}(\text{vec}(\boldsymbol{M}_{\boldsymbol{A}^{(sr)}}), \boldsymbol{\Sigma}_{\boldsymbol{A}^{(sr)}})$$

$$\boldsymbol{\Sigma}_{\boldsymbol{A}^{(sr)}} = \Big(\alpha \boldsymbol{I}_M \otimes \boldsymbol{K}_{\boldsymbol{A}}^{-1} + \langle\psi^{(sr)}\rangle\langle(\boldsymbol{P}^{(s)})^{\mathrm{T}}\boldsymbol{P}^{(s)}\rangle \otimes \boldsymbol{I}_T\Big)^{-1}$$

$$\text{vec}(\boldsymbol{M}_{\boldsymbol{A}^{(sr)}}) = \langle\psi^{(sr)}\rangle\boldsymbol{\Sigma}_{\boldsymbol{A}^{(sr)}}\,\text{vec}\Big(\langle\boldsymbol{P}^{(s)}\rangle^{\mathrm{T}}\Big(\boldsymbol{D}^{(sr)} - \langle\boldsymbol{\nu}^{(sr)}\rangle\boldsymbol{1}_{1\times T}\Big)\Big)$$

1.5.1 Precision

$$p(\alpha) = \Gamma(a_\alpha, b_\alpha)$$

$$q(\alpha) = \Gamma(c_\alpha, d_\alpha)$$

$$c_\alpha = a_\alpha + \frac{TM}{2}\sum_{s=1}^{S} R(s)$$

$$d_\alpha = b_\alpha + \frac{1}{2}\sum_{s=1}^{S}\sum_{r=1}^{R(s)}\sum_{m=1}^{M}\langle\boldsymbol{A}_m^{(sr)}\boldsymbol{K}_{\boldsymbol{A}}^{-1}(\boldsymbol{A}_m^{(sr)})^{\mathrm{T}}\rangle$$

1.6 Noise

1.6.1 Precision

$$p(\psi^{(sr)}) = \Gamma(a_{\psi^{(sr)}}, b_{\psi^{(sr)}})$$

$$q(\psi^{(sr)}) = \Gamma(c_{\psi^{(sr)}}, d_{\psi^{(sr)}})$$

$$c_{\psi^{(sr)}} = a_{\psi^{(sr)}} + \frac{VT}{2}$$

$$\begin{aligned} d_{\psi^{(sr)}} = b_{\psi^{(sr)}} &+ \frac{1}{2}\,\text{Tr}\big((\boldsymbol{D}^{(sr)})^{\mathrm{T}}\boldsymbol{D}^{(sr)}\big) - \text{Tr}\big((\boldsymbol{D}^{(sr)})^{\mathrm{T}}\langle\boldsymbol{P}^{(s)}\rangle\langle\boldsymbol{A}^{(sr)}\rangle\big) \\ &- \sum_{t=1}^{T}\langle\boldsymbol{\nu}^{(sr)}\rangle^{\mathrm{T}}\boldsymbol{D}_t^{(sr)} + \sum_{t=1}^{T}\langle\boldsymbol{\nu}^{(sr)}\rangle^{\mathrm{T}}\langle\boldsymbol{P}^{(s)}\rangle\langle\boldsymbol{A}_t^{(sr)}\rangle \\ &+ \frac{1}{2}\,\text{Tr}\big(\langle(\boldsymbol{P}^{(s)})^{\mathrm{T}}\boldsymbol{P}^{(s)}\rangle\langle\boldsymbol{A}^{(sr)}(\boldsymbol{A}^{(sr)})^{\mathrm{T}}\rangle\big) + \frac{T}{2}\langle\boldsymbol{\nu}^{(sr)}\rangle^{\mathrm{T}}\langle\boldsymbol{\nu}^{(sr)}\rangle \end{aligned}$$

1.6.2 Mean

$$p(\boldsymbol{v}^{(sr)}) = \mathcal{N}(\mathbf{0}, \eta_{\boldsymbol{v}^{(sr)}}^{-1} \boldsymbol{I}_V)$$

$$q(\boldsymbol{v}^{(sr)}) = \mathcal{N}(\boldsymbol{m}_{\boldsymbol{v}^{(sr)}}, \boldsymbol{\Sigma}_{\boldsymbol{v}^{(sr)}})$$

$$\boldsymbol{\Sigma}_{\boldsymbol{v}^{(sr)}} = \left(\eta_{\boldsymbol{v}^{(sr)}} + T\langle\psi^{(sr)}\rangle\right)^{-1} \boldsymbol{I}_V$$

$$\boldsymbol{m}_{\boldsymbol{v}^{(sr)}} = \langle\psi^{(sr)}\rangle \boldsymbol{\Sigma}_{\boldsymbol{v}^{(sr)}} \sum_{t=1}^{T} \boldsymbol{D}_t^{(sr)} - \langle\boldsymbol{P}^{(s)}\rangle\langle\boldsymbol{A}_t^{(sr)}\rangle$$

2 Algorithm

2.1 Initialisation

In practice, we found that our algorithm would not stably converge if the spatial maps were simply initialised with Gaussian noise. Therefore, we use two slightly more complex initialisation strategies.

For the simulated data we used a technique whereby we dynamically switched models as the algorithm was converging. The full model was the one outlined above, but we also utilised a reduced model where spatial variability across subjects was 'turned off'. This reduced model has a set of group-level mode maps, $\boldsymbol{P}^{(g)}$, which are used to model all subjects simultaneously i.e. for every subject, $\boldsymbol{P}^{(s)} = \boldsymbol{P}^{(g)}$. The prior on these group maps is equivalent to the spike-slab distribution on the voxelwise group means, μ.

The full procedure is therefore: generate an initial set of random Gaussian spatial maps; run the reduced model for 250 iterations; keeping all time course and noise posteriors constant, switch to the full model and run for 500 iterations; switch back to the reduced model and run for 500 iterations; switch to the full model and run for 1250 iterations, or until the rate of change of the free energy indicates the model has converged satisfactorily. This is of course a heuristic approach but one that seems to give stable, repeatable performance. Our interpretation is that the reduced model is much more robust to subject variability, subject specific artefacts etc so can generate a reasonable estimate for the PFMs from a random starting point. We also observed that switching models multiple times improved performance—again, we believe that this arises as the trade-off between the better characterisation of the PFMs by the full model, and the robustness of the reduced model, is a very complicated one.

The HCP results we have presented use a slightly different strategy, though the motivation to produce a stable set of maps and time courses, with which to initialise the full spatial model, is essentially the same. Firstly, a random set of spatial maps, again composed of Gaussian noise, are generated. This is followed by three iterations of dual regression—for each iteration, time courses are generated by regressing the spatial maps out of the data, and then a new set of group-level spatial maps are generated by regressing these time courses out of the data. Therefore, the updated set of spatial maps are both informed by the data and associated with a stable set of time courses; this is analogous to the initialisation of the full model using the time courses from the reduced model.

The algorithm is much more stable from this starting configuration, though as can be seen from the test-retest convergence plot in the main paper, this is still very much a random configuration with very low similarities across runs. Finally, as

we observed that one 'global' PFM—containing large, positive weights in all spatial locations—would always be inferred, one of the initial maps is given this structure to speed up convergence.

2.2 Parameters

The parameters used to infer the PFMs from the simulated data are shown in table 1, where S, V, T etc all denote the sizes of the various data, as per usual. $\boldsymbol{K_A}$ was generated from the default SPM double-gamma HRF.

The parameters used for the HCP data were all the same as for the simulated data, with the exception that λ is changed to 0.05.

Parameter	Value
λ	0.1
a_α, b_α	$\frac{1}{2}TV\sum_{s=1}^{S} R(s)$
a_β, b_β	$0.1S$
a_γ, b_γ	1
a_π	$0.005S$
b_π	$0.045S$
a_ψ, b_ψ	1
η	0.1

Table 1: Parameters used when inferring PFMs from simulated data.

3 Simulated Data

3.1 Data generation

We will use the following variables to describe the size of the various simulated data: V voxels; T time points; S subjects; $R(s)$ runs for subject s; N parcels; M modes.

We will frequently want to induce correlations between modes and we do this by taking linear combinations. For example, to form a set of correlated time courses, $\boldsymbol{A}'$, from an original set, $\boldsymbol{A}$, we simply use a mixing matrix, $\boldsymbol{L}$, to give $\boldsymbol{A}' = \boldsymbol{AL}$. The mixing matrices we use here will all be formed as $\boldsymbol{L} = \boldsymbol{I} + k\boldsymbol{R}$, where k is a simple scale factor and $\boldsymbol{R}$ is a random matrix, with elements drawn independently from a triangular distribution ($a = -1$, $b = 1$, $c = 0$). In subsequent sections, we will simply parameterise this mixing process by k.

3.1.1 Spatial atlas

The sizes of the binary blocks that make up the atlas are simply sampled from a Dirichlet distribution ($\boldsymbol{\alpha} = 25 \times \mathbf{1}_{N\times 1}$).

The subject variability is introduced by randomly warping this group atlas to simulate mismatches in the spatial locations of regions between subjects. We enforce that this is smooth, or in other words, the warping cannot re-order voxels. This is simply a constraint on the gradient of the warp, which we outline below.

Let v index the spatial location at the group level, and v_s be the subject-specific equivalent. The subject maps can be formed by interpolating the group maps according

to these spatial indices, as $P^{(s)}_{v_s} = P_v$. A perfect registration would therefore be when $v_s = v$. The warp, w_s, defines how the subject location varies with respect to the group, which gives $v_s = v + w_s$. Our definition of a smooth warp is simply that the mapping from group to subject location should be monotonic, or in terms of the terms we have defined,

$$\begin{aligned} \frac{\mathrm{d}v_s}{\mathrm{d}v} &= 1 + \frac{\mathrm{d}w_s}{\mathrm{d}v} \\ &\geq 0 \end{aligned}$$

We simulate the gradient of the warp field and integrate this to get w_s. A scaling is then applied to ensure the warp lies within the maximum allowable displacement. The subject maps are formed by interpolating the group maps at the subject-specific spatial locations. The gradient is generated by simulating Gaussian noise at each of the voxel locations, which is then convolved with a boxcar function (width $= \frac{V}{20}$) to introduce some spatial smoothness. In order to enforce monotonicity, this gradient is normalised to unity standard deviation and passed through a sigmoid function (thereby, somewhat arbitrarily, restricting the positive rate of change of the warp too).

3.1.2 Mode weights

Modes are required to be simultaneously sparse and spatially structured. We therefore simulate each mode as being formed from several 'blocks'—these are groups of weights that are similar in spatially adjacent parcels. The way the weights in each group-level mode are generated is described below.

Firstly, a sparsity parameter is drawn from a beta distribution (mean $= \frac{2}{N}$, variance $=$ 0.00075). Then, the number of blocks (B) is drawn from a Poisson distribution ($\lambda = 4$) and the block lengths are drawn from a Dirichlet distribution ($\boldsymbol{\alpha}_1 = 10 \max(1, B-1)$, $\boldsymbol{\alpha}_{2,\dots,B} = 10$). These are scaled by the number of voxels and the sparsity parameter to get the lengths in numbers of voxels. The block locations are randomly drawn such that there is a slight bias against blocks overlapping with the blocks in previously simulated modes. However, as we specify that the mean sparsity times the number of modes must be greater than one, strong spatial correlations still exist, as can be seen from the figure in the main body of the paper. The weights that are present within each block are simulated as a small positive constant (0.5) plus gamma distributed random variables ($a = 3$, $b = 2$), before 30% of blocks are randomly set as negative, whereby the signs of the weights within the block are flipped. Finally, the mode weights are convolved with a narrow boxcar function (width $= \frac{N}{100}$).

Subject specific mode weights are generated by adding sparse noise, generated from a spike-slab distribution ($\lambda = 0.25$, $\sigma = 1$), to the group weights. The noise is correlated ($k = 0.5$) and is scaled such that the standard deviation of the noise is half the standard deviation of the group maps.

3.1.3 Neural time courses

One of the aims is to investigate how non-white neural time courses affect inference. In this case, this is simply a process where there was an excess of power below 0.1Hz. This is achieved by randomly generating time courses in frequency space. The DFT weights are generated as an amplitude (each amplitude is drawn from a rectified normal distribution, with weights at frequencies below 0.1Hz doubled) and a uniformly distributed phase.

In order to induce correlations, linear combinations of these time courses are produced by simple matrix multiplication. Three, differently scaled, mixing matrices

are used: a group matrix ($k = 0.3$); a subject matrix ($k = 0.5$); and a scan matrix ($k = 0.1$). The scaling factors allow the strength of the correlations, and their variability over subjects and scans, to be controlled.

In order to generate non-Gaussianities, the time courses are symmetrically thresholded, with sub-threshold activations set to zero, such that 20% of the activations remain. These sparse time courses are scaled to unity standard deviation, and a small amount of Gaussian noise is added ($\mu = 0$, $\sigma = 0.1$).

Finally, 1% of time courses are set to zero in order to investigate how the different methods cope if some modes are not present in all subjects.

3.1.4 BOLD signal

The BOLD signal represents the activity of the time courses after spatial mixing via the parcel atlas and mode weights, and the action through a nonlinear HRF.

For computational efficiency, we first simulate the signal arising from a linear HRF before saturating this to introduce nonlinearities. As both the spatial mixing and the HRF are linear we can compute the HRF of the time courses and then mix these convolved time courses with the spatial weights. Each time course is convolved with a different HRF, drawn from the FLOBS basis set[1]. We use the three basis functions with randomly drawn weights ($w_1 \sim \mathcal{N}(1, 0.1)$, $w_2 \sim \mathcal{N}(0, 0.1)$, $w_3 \sim \mathcal{N}(0, 0.1)$)

These convolved time courses are passed through the spatial weights to generate the BOLD signal in voxel space, which is then scaled and passed through a tanh function (parameterised such that the 99th percentile of the BOLD signal is reduced in amplitude by 10%). This nonlinearly saturates the peaks in the BOLD signal, which arise at time points when voxels receive contributions from different, co-activating modes.

3.1.5 Noise

Once the BOLD signal has been generated the last step is to add the noise. This is independently drawn at each voxel and time point, which gives noise that is both spatially and temporally white. The noise itself is very weakly non-Gaussian, drawn from a t-distribution with 16 degrees of freedom, and appropriately scaled to achieve the specified SNR (0.1).

3.1.6 Examples

Randomly simulated spatial maps are shown in figures S2, S3 and S4. Time course and BOLD signal properties are illustrated in figures S5, S6 and S7.

3.2 Dual regression

For all the ICA based methods we test, we also present the results after a dual regression step. In its standard form, this takes a set of group level spatial maps, performs a regression to extract the associated subject specific time courses from the data, and then performs a second regression whereby subject specific maps are extracted using the data and the subject time courses. For this paper, we actually perform one further regression when presenting the dual regression results. We take the subject specific maps and extract a further set of time courses that are consistent with those—these will be different to the time courses that are generated using the group maps.

As presented here, the standard results are based on the group maps and the time courses from the first regression. The dual regression results are based on the subject maps and the set of time courses, from the third regression, associated with them. While this is not the norm for these analyses, as our scoring methods are based on both spatial and temporal decomposition accuracies, we believe it is appropriate to

present for two reasons. Firstly, the spatial results are unaffected by this subsequent regression, so these will be consistent with the standard analysis pipeline. Secondly, the temporal results one would normally see can be evaluated simply by looking at the standard results, which contain the original time courses—by including the final set of time courses we avoid duplicating the temporal results for these methods.

3.3 Test-retest reliability versus ground truth accuracy

Here we provide a brief proof of the theoretical relationship between test-retest scores (TRR) and the ground-truth accuracy (GTA) that we plot on the relevant figures in the main paper.

Let the inferred maps from two different runs, $\boldsymbol{p}_1$ and $\boldsymbol{p}_2$, be the ground truth map, $\boldsymbol{g}$, with independent, zero-mean, additive noise, $\boldsymbol{\varepsilon}$.

$$\boldsymbol{p}_1 = \boldsymbol{g} + \boldsymbol{\varepsilon}_1 \qquad \boldsymbol{p}_2 = \boldsymbol{g} + \boldsymbol{\varepsilon}_2$$

$$\mathrm{E}[\boldsymbol{\varepsilon}_1^\mathrm{T}\boldsymbol{\varepsilon}_1] = \mathrm{E}[\boldsymbol{\varepsilon}_2^\mathrm{T}\boldsymbol{\varepsilon}_2] \qquad \mathrm{E}[\boldsymbol{\varepsilon}_1^\mathrm{T}\boldsymbol{\varepsilon}_2] = \mathrm{E}[\boldsymbol{\varepsilon}_1^\mathrm{T}\boldsymbol{g}] = 0$$

The scores we calculate are based on the correlation coefficients between these maps. The GTA is:

$$\begin{aligned}\mathrm{GTA}_1 &= \frac{\boldsymbol{g}^\mathrm{T}\boldsymbol{p}_1}{\sqrt{\boldsymbol{g}^\mathrm{T}\boldsymbol{g}}\sqrt{\boldsymbol{p}_1^\mathrm{T}\boldsymbol{p}_1}} \\ &= \frac{\boldsymbol{g}^\mathrm{T}\boldsymbol{g}}{\sqrt{\boldsymbol{g}^\mathrm{T}\boldsymbol{g}}\sqrt{\boldsymbol{g}^\mathrm{T}\boldsymbol{g} + \boldsymbol{\varepsilon}_1^\mathrm{T}\boldsymbol{\varepsilon}_1}} \\ &= \frac{\sqrt{\boldsymbol{g}^\mathrm{T}\boldsymbol{g}}}{\sqrt{\boldsymbol{g}^\mathrm{T}\boldsymbol{g} + \boldsymbol{\varepsilon}_1^\mathrm{T}\boldsymbol{\varepsilon}_1}}\end{aligned}$$

The TRR is:

$$\begin{aligned}\mathrm{TRR} &= \frac{\boldsymbol{p}_1^\mathrm{T}\boldsymbol{p}_2}{\sqrt{\boldsymbol{p}_1^\mathrm{T}\boldsymbol{p}_1}\sqrt{\boldsymbol{p}_2^\mathrm{T}\boldsymbol{p}_2}} \\ &= \frac{\boldsymbol{g}^\mathrm{T}\boldsymbol{g}}{\sqrt{\boldsymbol{g}^\mathrm{T}\boldsymbol{g} + \boldsymbol{\varepsilon}_1^\mathrm{T}\boldsymbol{\varepsilon}_1}\sqrt{\boldsymbol{g}^\mathrm{T}\boldsymbol{g} + \boldsymbol{\varepsilon}_2^\mathrm{T}\boldsymbol{\varepsilon}_2}}\end{aligned}$$

Therefore, in this scenario, the TRR is simply the GTA squared.

3.4 Simulation results with mismatched mode numbers

In figures S8, S9, S10, S11 and S12 we show the accuracy of recovery of the ground truth for the various methods under test. For each scoring method we show the accuracy when the methods are tasked with recovering either 15, 25 or 40 modes. The ground truth consisted of a set of 25 modes.

The comparisons between ground truth accuracy and test-retest reliability are shown in figures S13, S14, S15 and S16.

3.5 Performance of PCA

Given the relatively poor performance of PCA at recovering maps or time courses, it is perhaps somewhat surprising that it is almost the best method for recovering spatial correlations.

We run the PCA decomposition on the temporally concatenated data and, to calculate the scores, we set all the subject maps to these concatenated PCA maps and

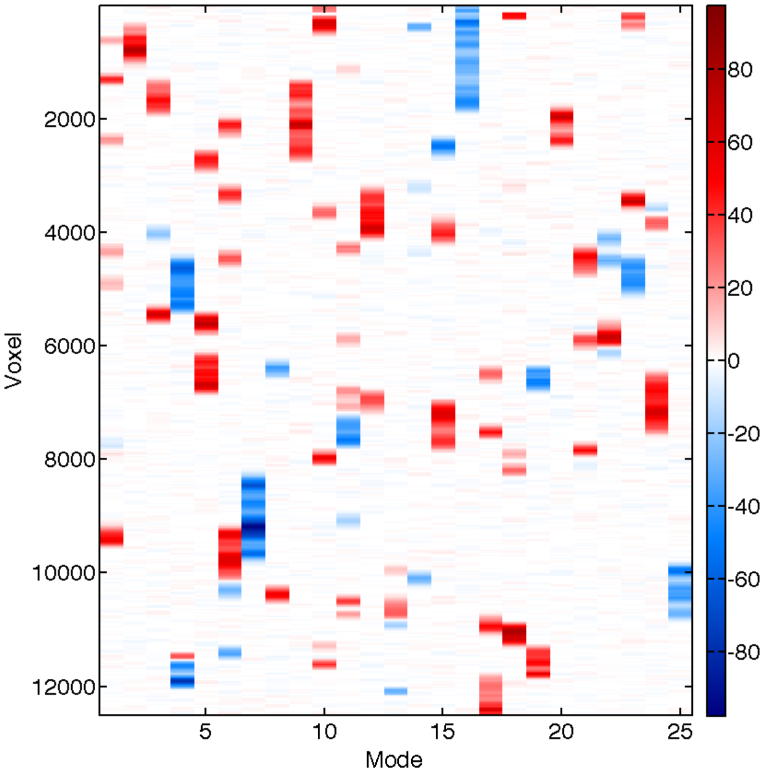

Supplementary figure S2: Mean, over subjects, of a randomly generated set of spatial maps. These have been multiplied out into voxel space using the subject atlases.

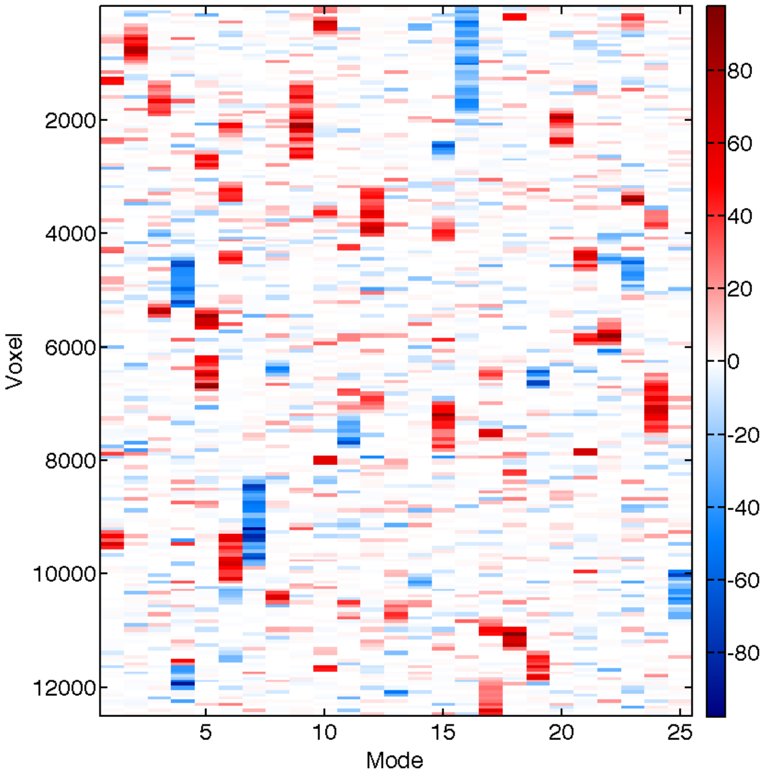

Supplementary figure S3: A randomly generated set of subject specific mode spatial maps. This has been multiplied out into voxel space using the subject atlas.

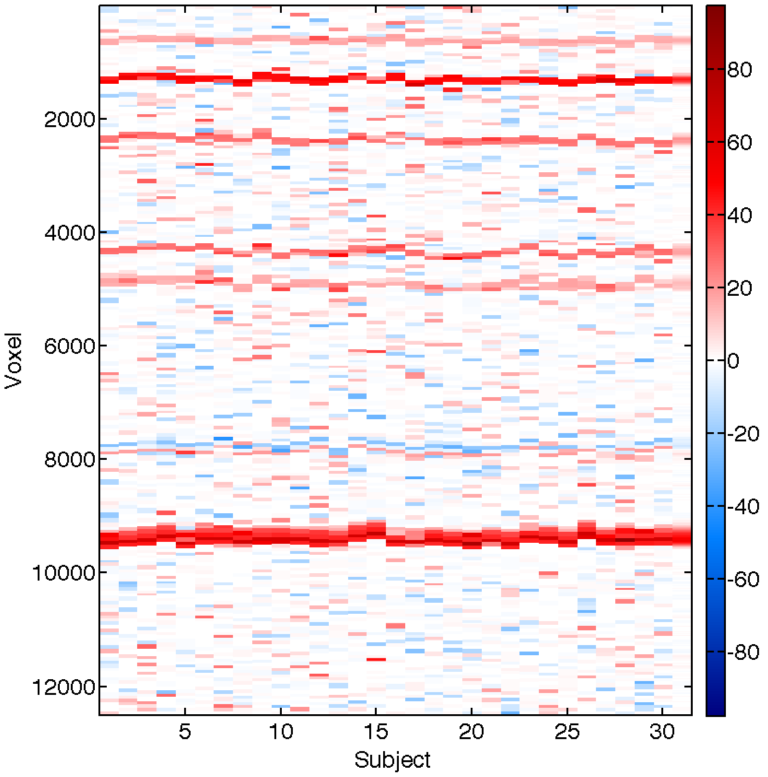

Supplementary figure S4: All subject specific spatial maps for mode 1 from figure S2. These have been multiplied out into voxel space using the subject atlas. The mean map is shown at the far right.

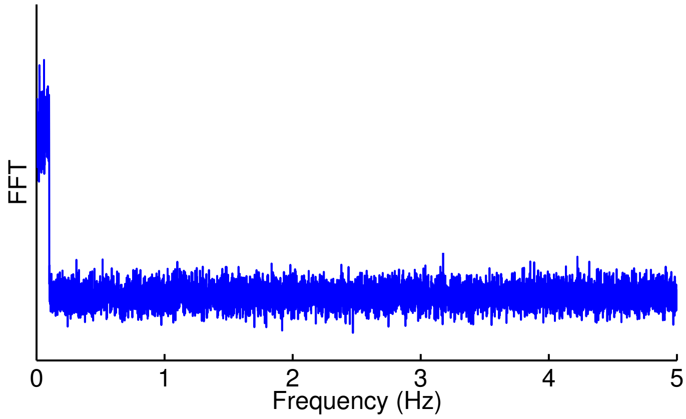

Supplementary figure S5: Example of the frequency content of a simulated neuronal signal.

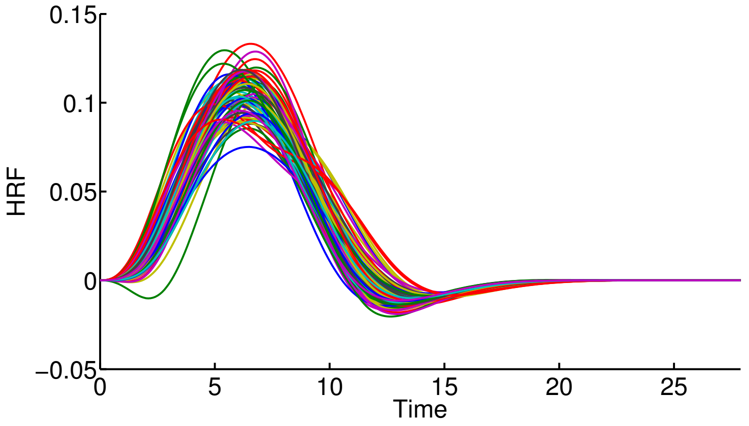

Supplementary figure S6: Typical variability in the HRFs drawn from the linear basis set. The nonlinearities are applied after this convolution.

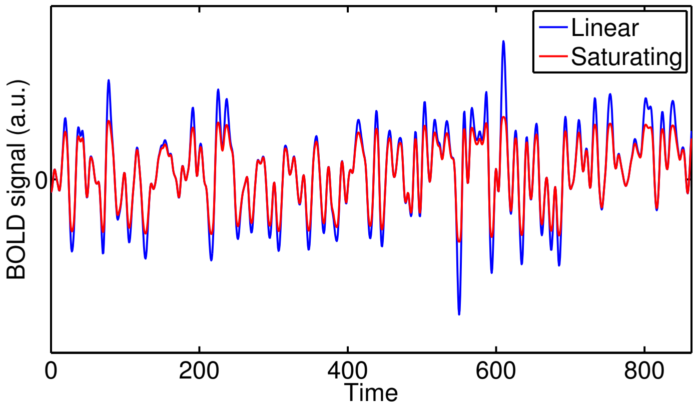

Supplementary figure S7: Example BOLD time courses, after both the linear HRF and the subsequent nonlinear saturation.

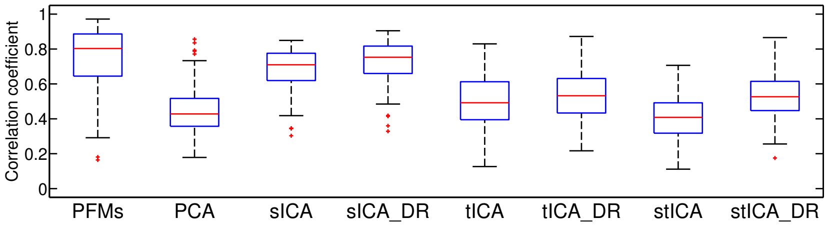

(a) 15 modes inferred.

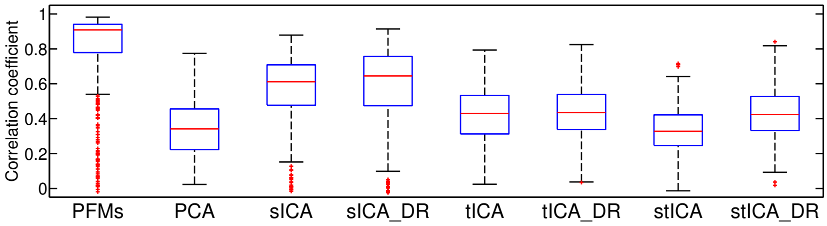

(b) 25 modes inferred.

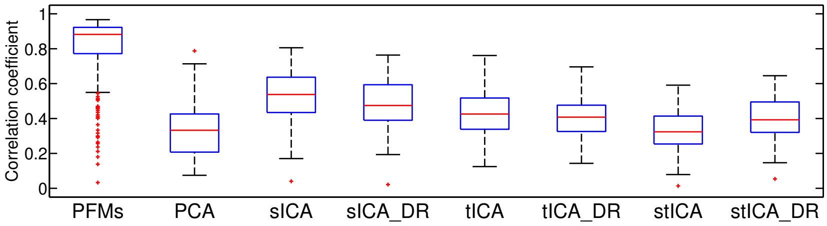

(c) 40 modes inferred.

Supplementary figure S8: Accuracy in recovery of ground truth subject-specific spatial maps on simulated data. Multiple data sets were simulated, and the mean correlation coefficient, over subject maps, between the true and inferred spatial maps is shown for each method. Dual regression is indicated by the suffix DR.

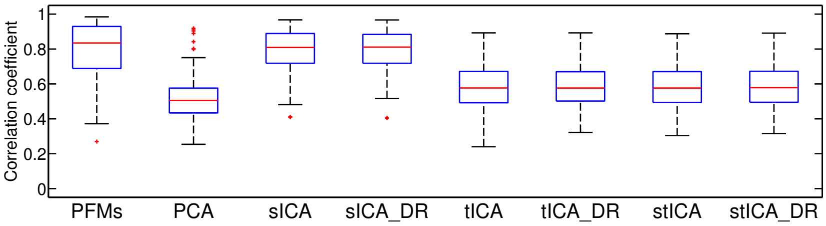

(a) 15 modes inferred.

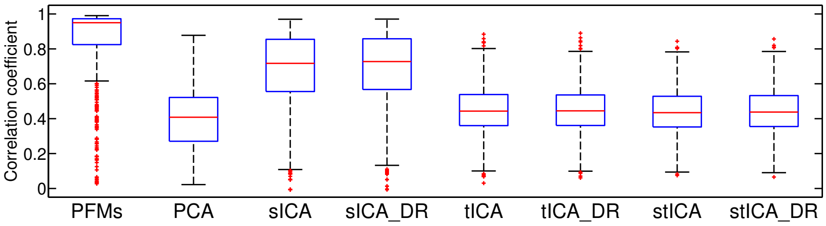

(b) 25 modes inferred.

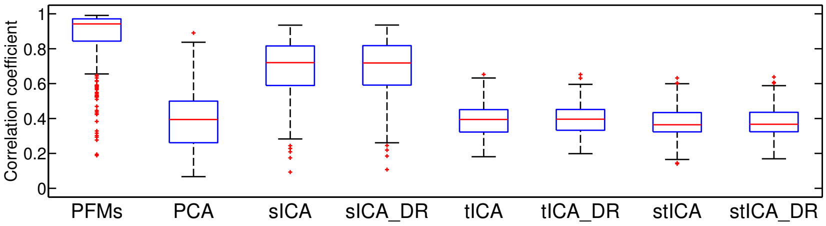

(c) 40 modes inferred.

Supplementary figure S9: Accuracy in recovery of ground truth time courses on simulated data. Multiple data sets were simulated, and the mean correlation coefficient, over subjects and runs, between the true and inferred time courses is shown for each method. Dual regression is indicated by the suffix DR.

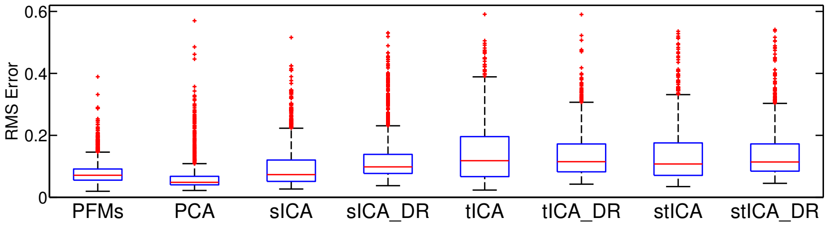

(a) 15 modes inferred.

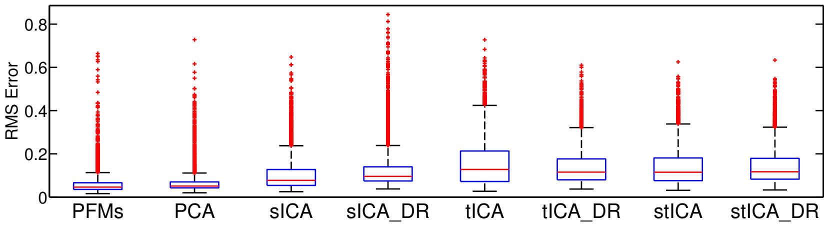

(b) 25 modes inferred.

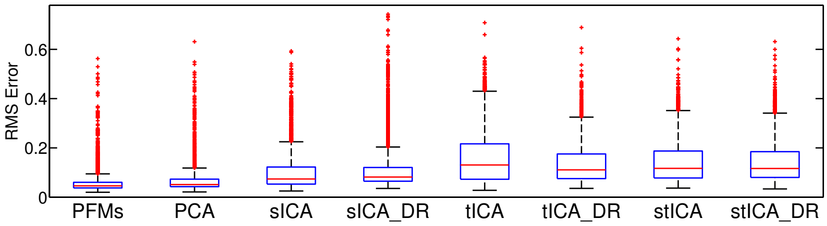

(c) 40 modes inferred.

Supplementary figure S10: Accuracy in recovery of ground truth subject-specific between-mode spatial correlations on simulated data. Multiple data sets were simulated, and the RMS error, over subjects, between the true and inferred correlation coefficients is shown for each method. Dual regression is indicated by the suffix DR.

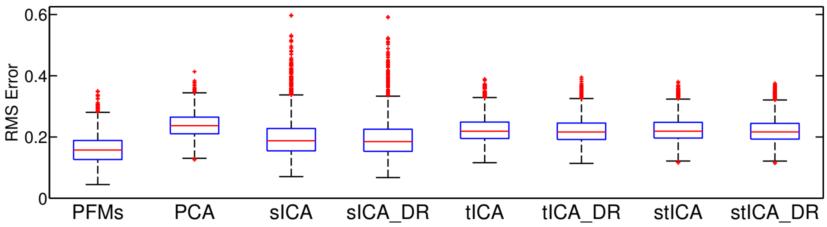

(a) 15 modes inferred.

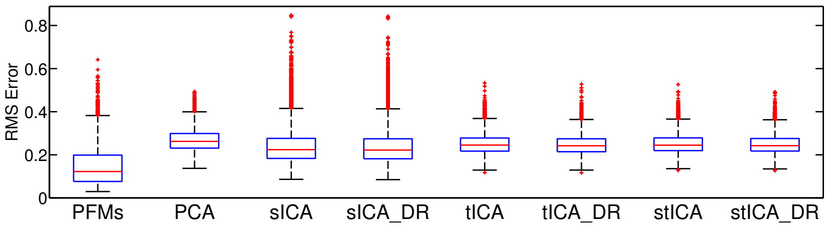

(b) 25 modes inferred.

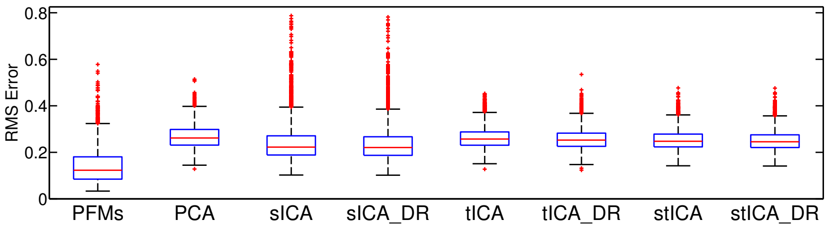

(c) 40 modes inferred.

Supplementary figure S11: Accuracy in recovery of ground truth subject-specific between-mode temporal correlations on simulated data. Multiple data sets were simulated, and the RMS error, over subjects, between the true and inferred correlation coefficients is shown for each method. Dual regression is indicated by the suffix DR.

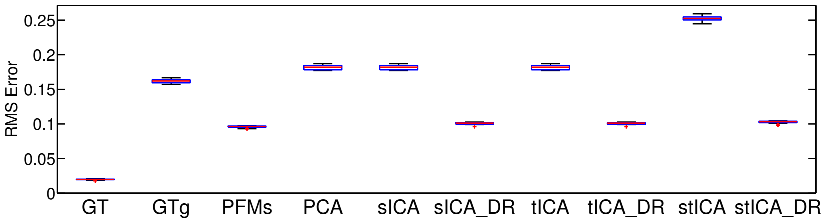

(a) 15 modes inferred.

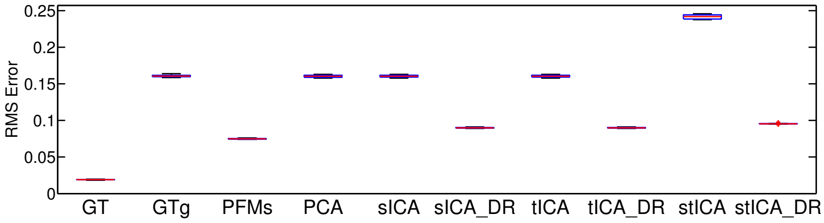

(b) 25 modes inferred.

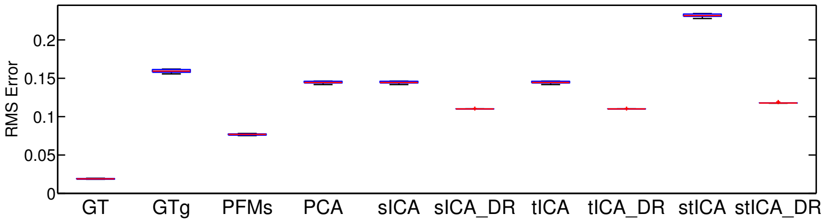

(c) 40 modes inferred.

Supplementary figure S12: Accuracy in recovery of the BOLD signal subspace on simulated data. Multiple data sets were simulated, and the RMS error, over subjects and scans, between the true and inferred BOLD signal is shown for each method. Dual regression is indicated by the suffix DR.

GT illustrates the performance of the optimal linear decomposition based on the ground truth spatial maps—this demonstrates the impact of the HRF nonlinearities. GTg illustrates the scores that are achieved if the subject maps are just set to the mean of the ground truth subject maps; as such, it both illustrates the amount of subject variability in the data and is also a useful benchmark for those methods which do not model individual subjects.

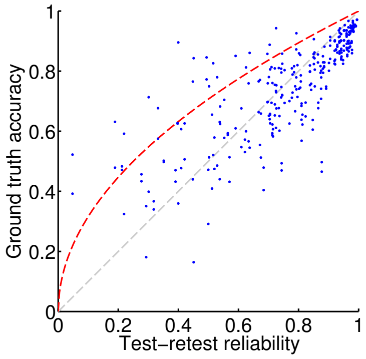

(a) 15 modes inferred.

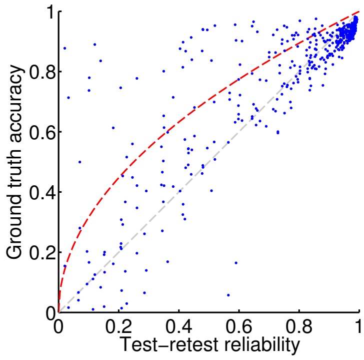

(b) 25 modes inferred.

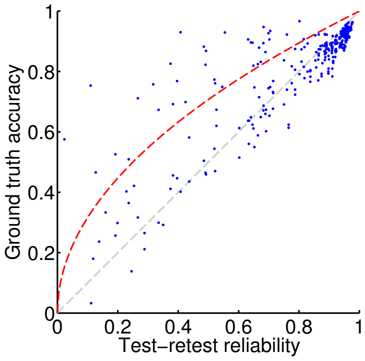

(c) 40 modes inferred.

Supplementary figure S13: Accuracy in recovery of ground truth subject-specific spatial maps, plotted against test-retest reliability, for the PFMs inferred from simulated data. The method was run twice on the same data set; both the accuracy scores, as plotted in figure S8, and the test-retest reliability, scored using the same correlation metric, were calculated for each mode.

The grey line indicates equality between the two scores, whereas the red line indicates the range of scores possible if the inferred maps are just the ground truth maps with independent additive noise.

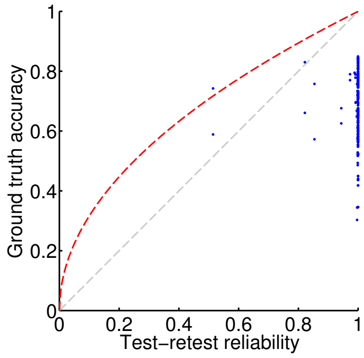

(a) 15 modes inferred.

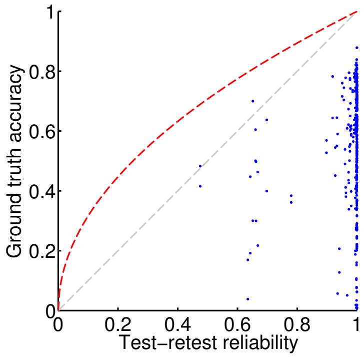

(b) 25 modes inferred.

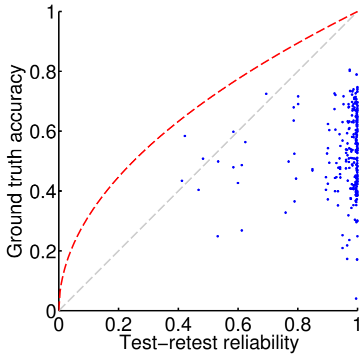

(c) 40 modes inferred.

Supplementary figure S14: Accuracy in recovery of ground truth subject-specific spatial maps, plotted against test-retest reliability, for sICA run with dual regression on simulated data. The method was run twice on the same data set; both the accuracy scores, as plotted in figure S8, and the test-retest reliability, scored using the same correlation metric, were calculated for each mode.

The grey line indicates equality between the two scores, whereas the red line indicates the range of scores possible if the inferred maps are just the ground truth maps with independent additive noise.

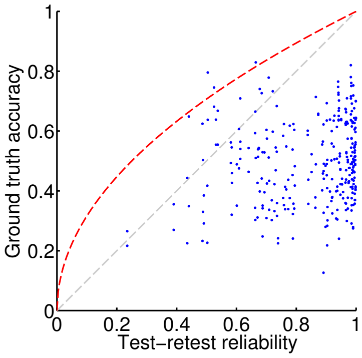

(a) 15 modes inferred.

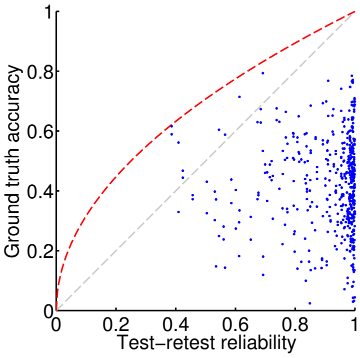

(b) 25 modes inferred.

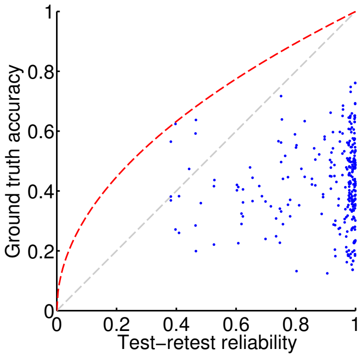

(c) 40 modes inferred.

Supplementary figure S15: Accuracy in recovery of ground truth subject-specific spatial maps, plotted against test-retest reliability, for tICA run with dual regression on simulated data. The method was run twice on the same data set; both the accuracy scores, as plotted in figure S8, and the test-retest reliability, scored using the same correlation metric, were calculated for each mode.

The grey line indicates equality between the two scores, whereas the red line indicates the range of scores possible if the inferred maps are just the ground truth maps with independent additive noise.

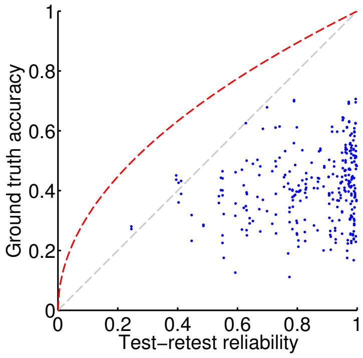

(a) 15 modes inferred.

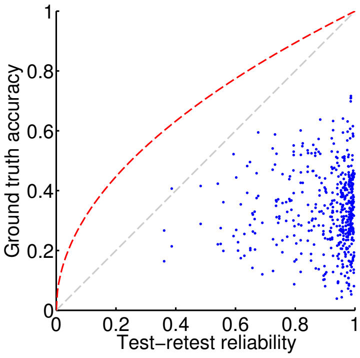

(b) 25 modes inferred.

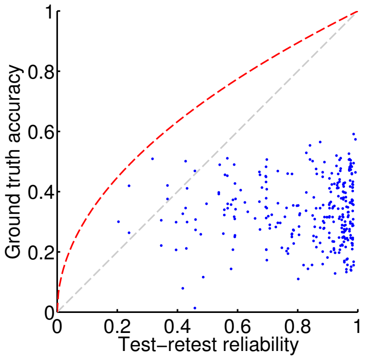

(c) 40 modes inferred.

Supplementary figure S16: Accuracy in recovery of ground truth subject-specific spatial maps, plotted against test-retest reliability, for stICA run with dual regression on simulated data. The method was run twice on the same data set; both the accuracy scores, as plotted in figure S8, and the test-retest reliability, scored using the same correlation metric, were calculated for each mode.

The grey line indicates equality between the two scores, whereas the red line indicates the range of scores possible if the inferred maps are just the ground truth maps with independent additive noise.

then split the PCA time courses into subject-specific segments. Therefore, by definition, the spatial maps will be orthogonal whereas this is not the case for the subject time courses—this is only guaranteed for the entire concatenated time courses.

As we do not demean spatial maps when calculating the correlations, orthogonal maps implies that the spatial PCA correlation coefficients will be zero. Therefore, the interpretation is that, by this measure, the ICA methods are actually performing worse than setting the correlations to zero. In the time domain, where orthogonality is not guaranteed, the performance of PCA is back in line with the ICA methods.

4 HCP Results

The full set of estimated PFM, sICA and tICA group maps can be found at the end of this document. We present the posterior mean for the PFMs, as in the main body of the paper, and the inferred spatial weights for ICA. All maps from a given algorithm are shown with the same colour scale. For the PFMs, the colour scale is from −6 to 6, whereas for both ICA approaches the colour scale goes from −17.5 to 17.5.

The PFM test-retest reliabilities are given in table 2. We also illustrate the subcortical involvement for each of the PFMs in figure S17.

PFM	TRR	PFM	TRR	PFM	TRR
1	0.9978	11	0.9172	21	0.1243
2	0.9256	12	0.2105	22	0.7418
3	0.9852	13	0.8607	23	0.8854
4	0.7980	14	0.8444	24	0.8627
5	0.9032	15	0.9034	25	0.6617
6	0.9672	16	0.9794	26	0.4059
7	0.9601	17	0.7681	27	0.3127
8	0.6964	18	0.9468	28	0.4135
9	0.9590	19	0.9096	29	0.1348
10	0.4031	20	0.8158	30	0.0844

Table 2: PFM test-retest reliabilities.

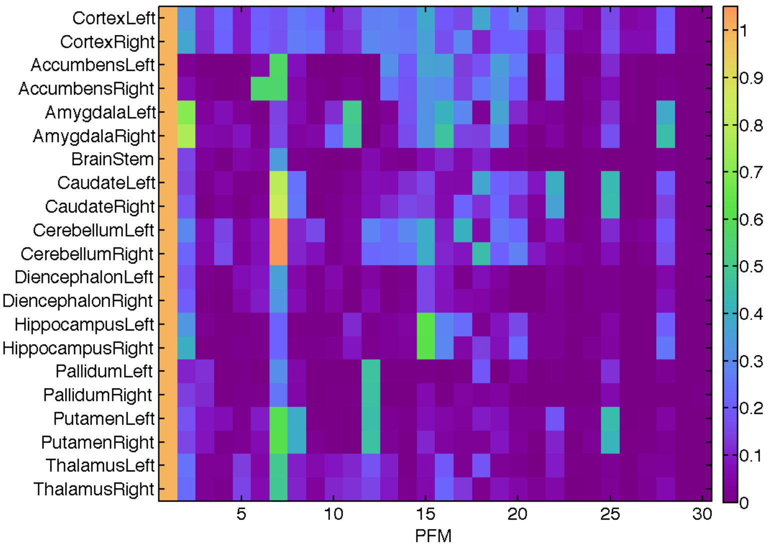

Supplementary figure S17: Subcortical weights for each PFM. For each PFM we plot the mean of the group weight magnitudes within each of the structures listed on the left of the figure. As the structures have very different baseline activities, we normalise the results for each structure by the mean weight for that structure in the global PFM (PFM 1).

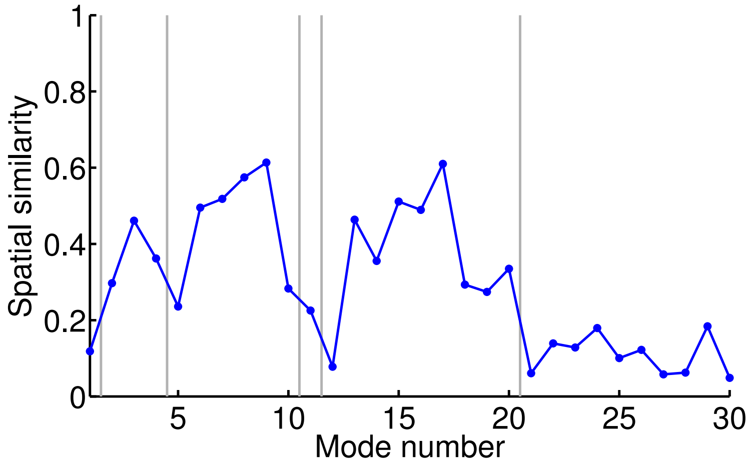

Supplementary figure S18: Spatial correlation coefficients between the sICA and tICA components. The modes are ordered as per the main body of the paper.

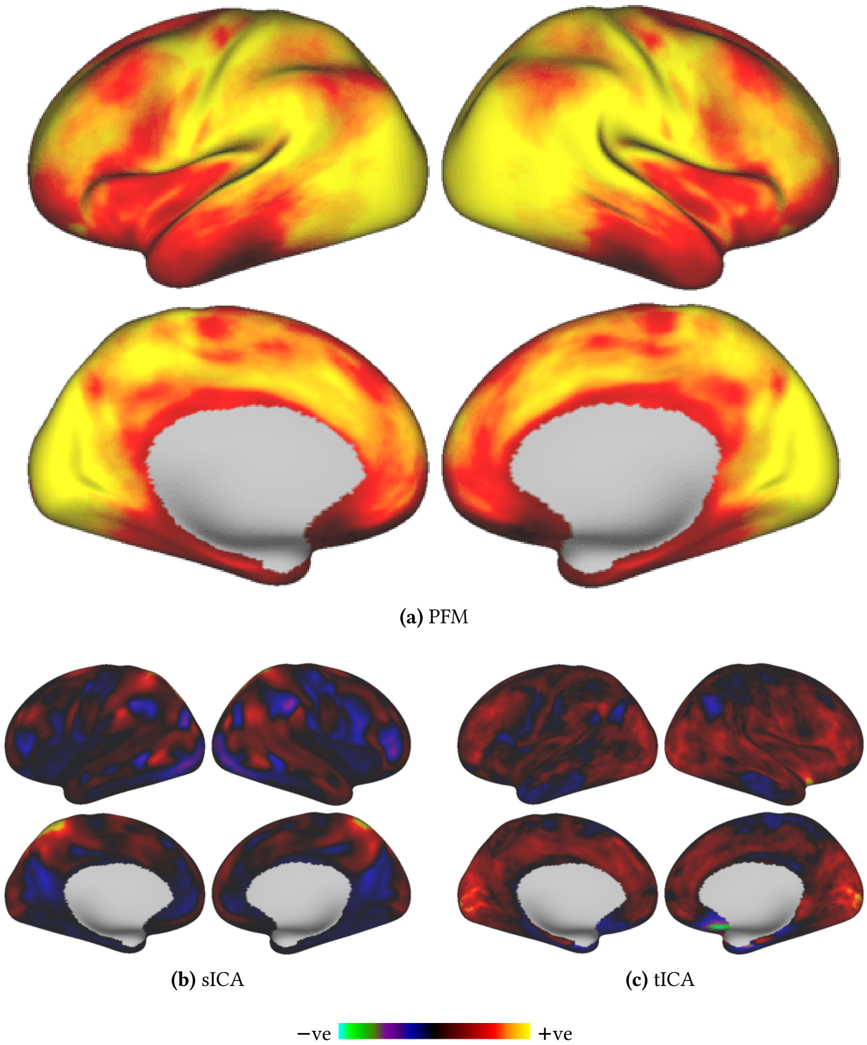

Supplementary figure S19: Component 1

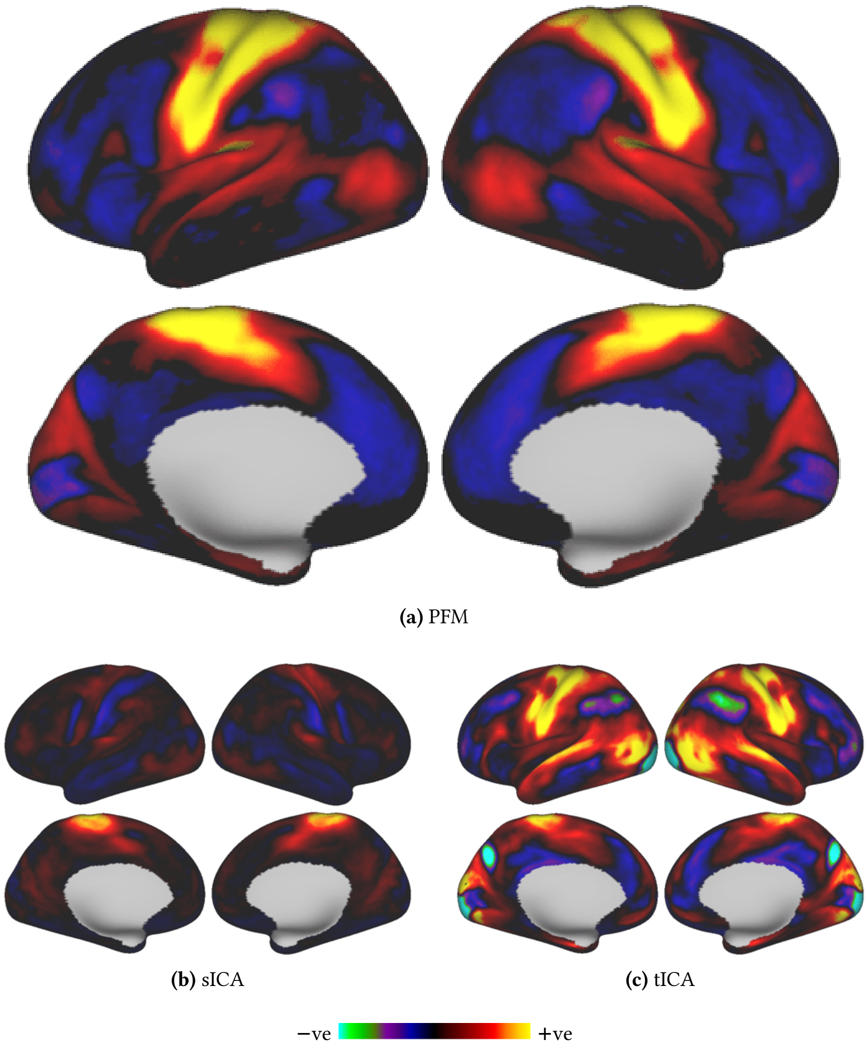

Supplementary figure S20: Component 2

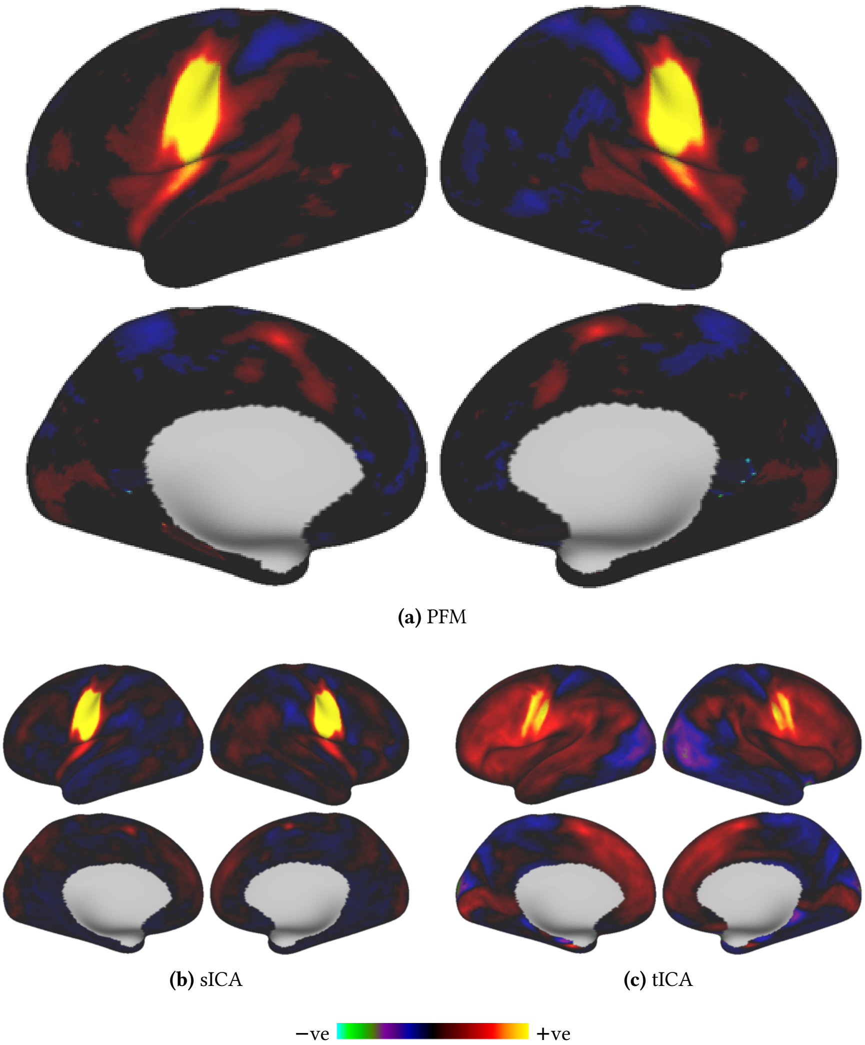

(a) PFM

(b) sICA

(c) tICA

Supplementary figure S21: Component 3

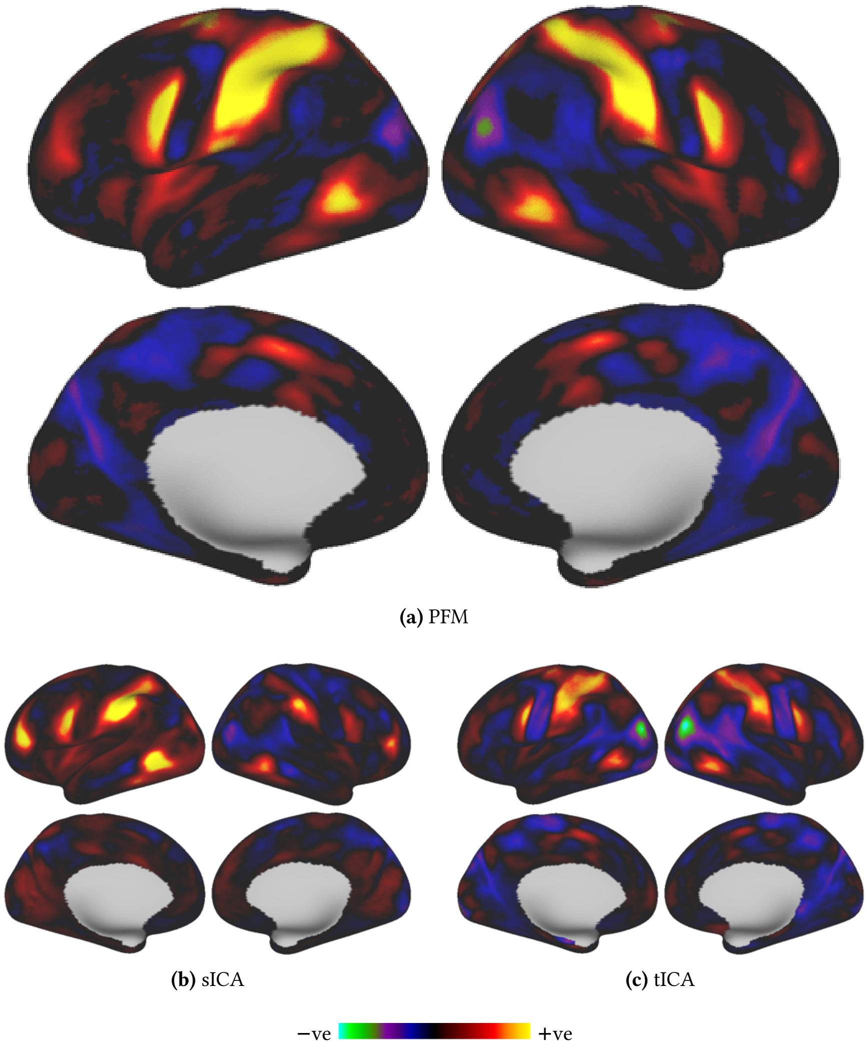

Supplementary figure S22: Component 4

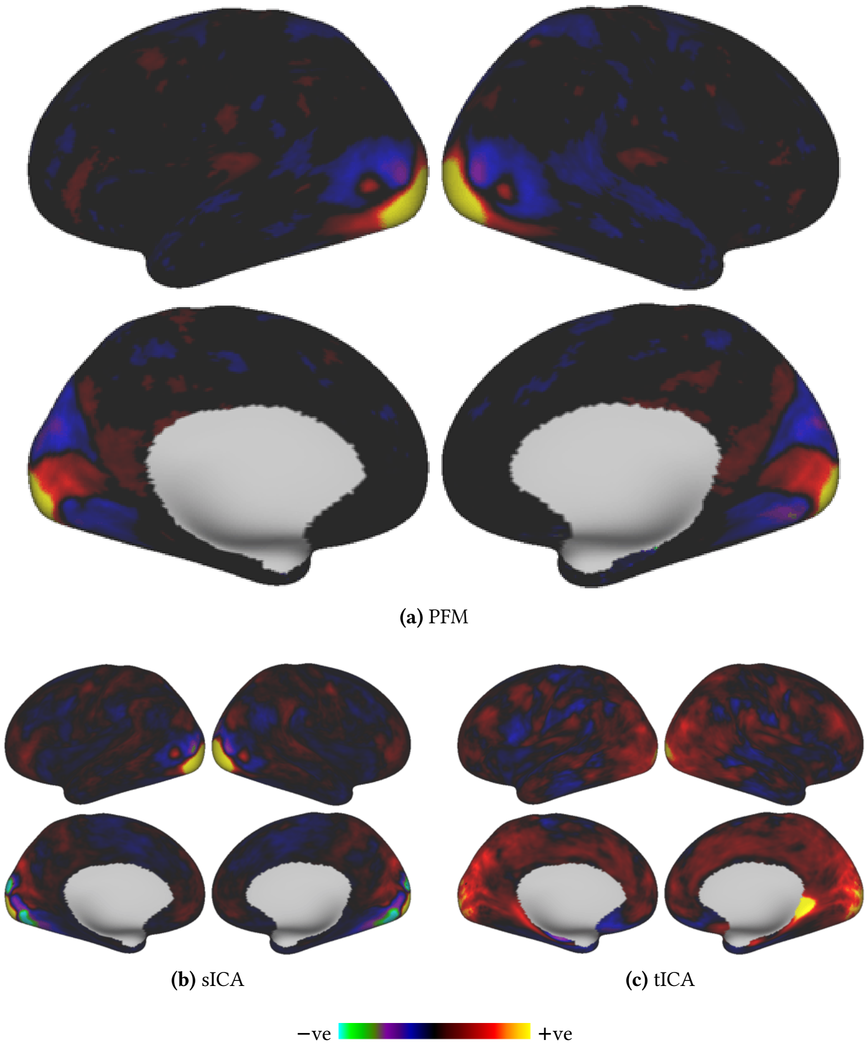

(a) PFM

(b) sICA

(c) tICA

Supplementary figure S23: Component 5

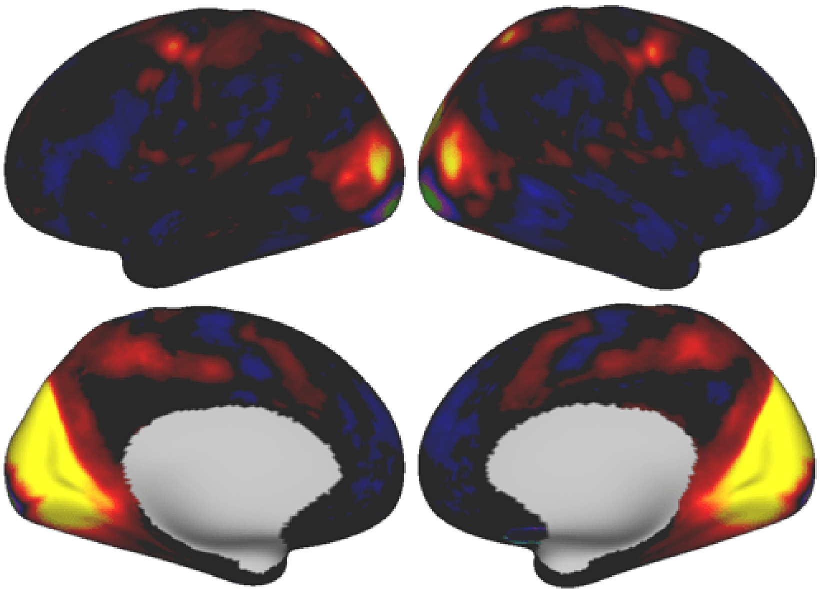

(a) PFM

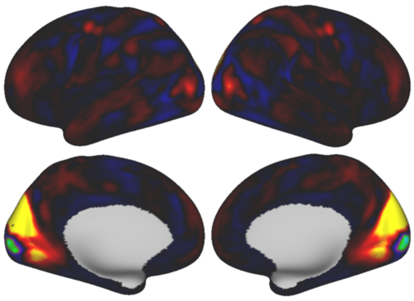

(b) sICA

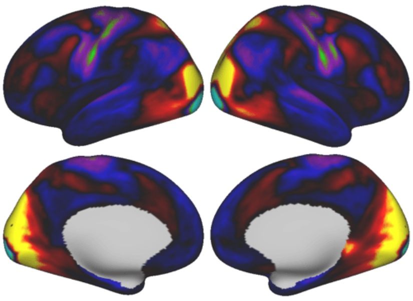

(c) tICA

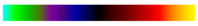

Supplementary figure S24: Component 6

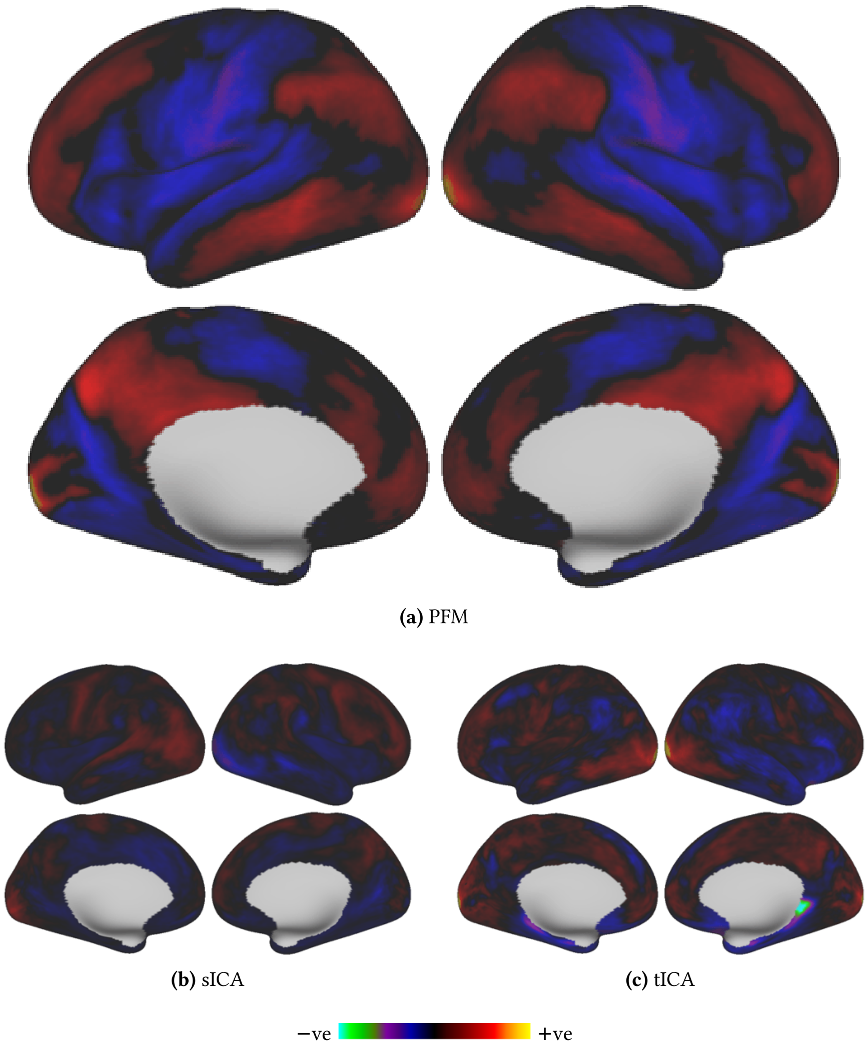

Supplementary figure S25: Component 7

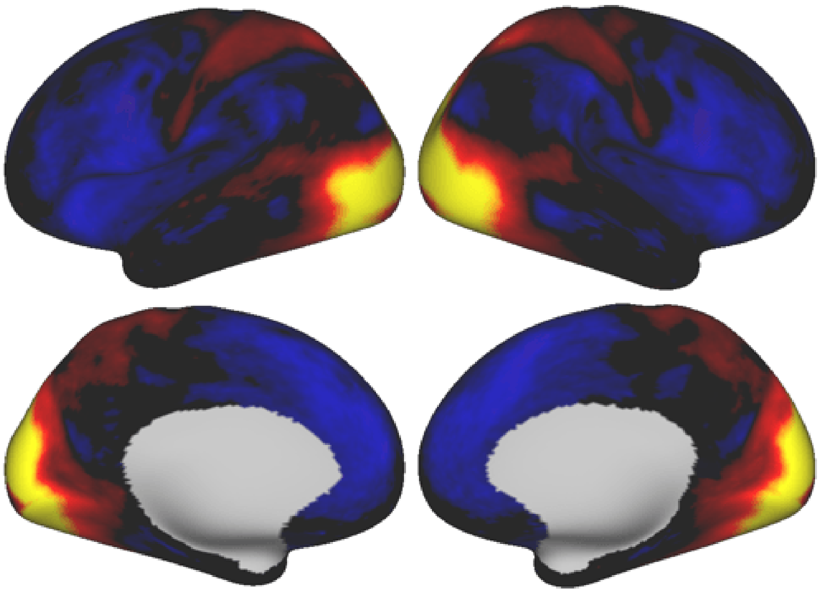

(a) PFM

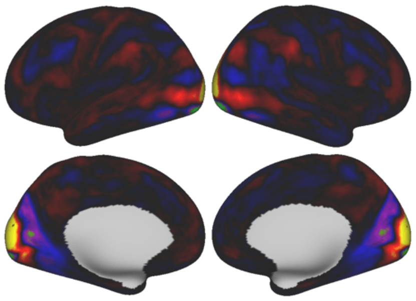

(b) sICA

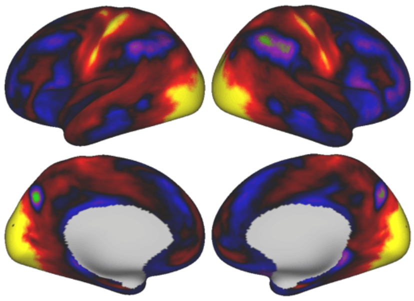

(c) tICA

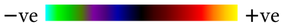

Supplementary figure S26: Component 8

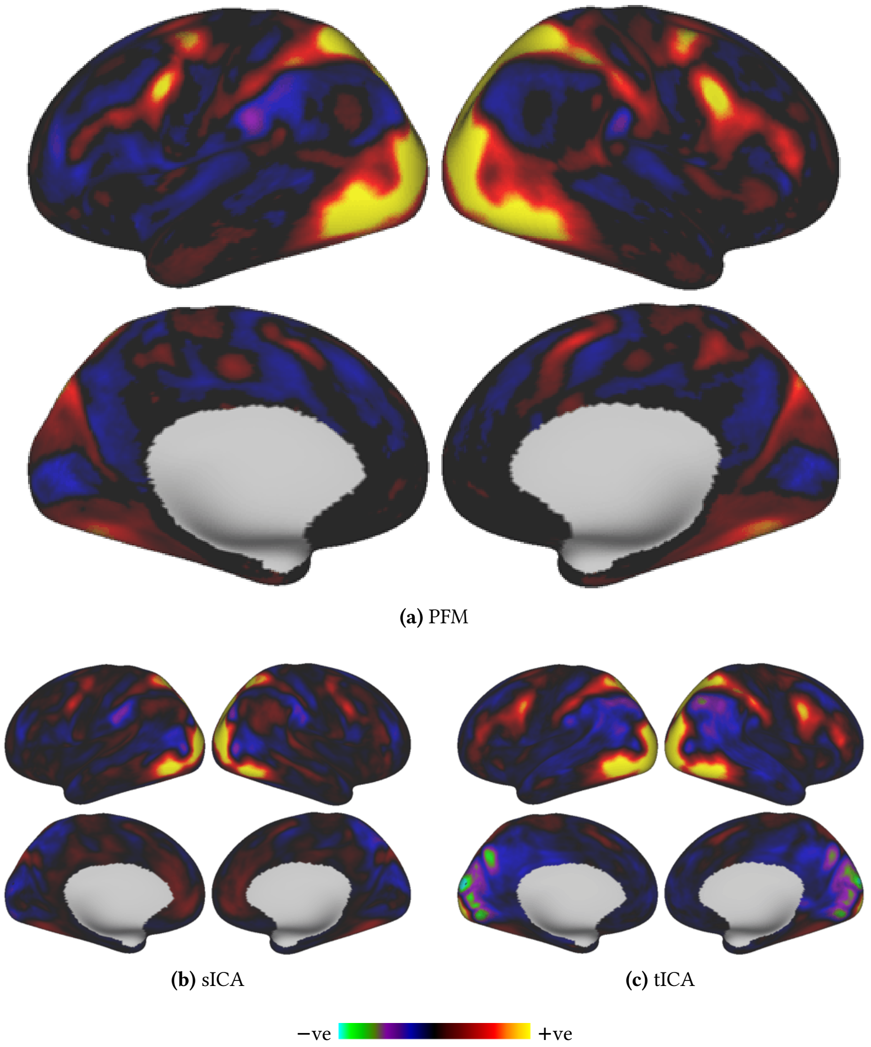

Supplementary figure S27: Component 9

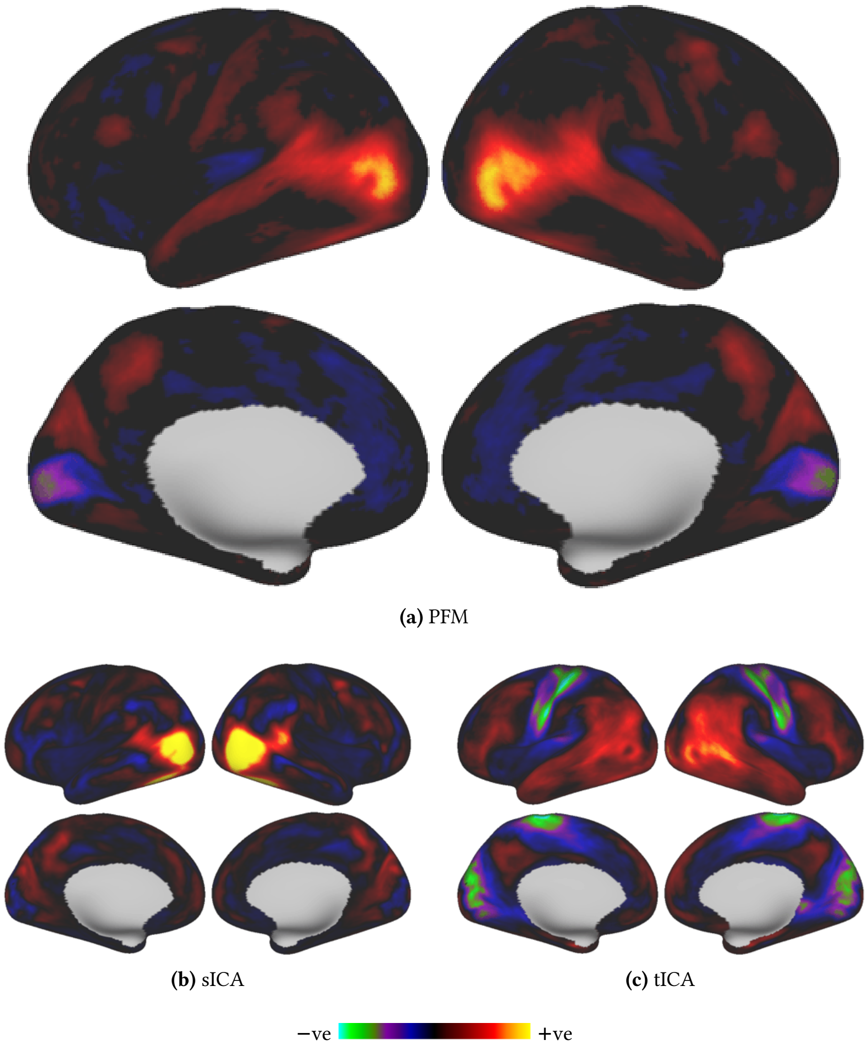

Supplementary figure S28: Component 10

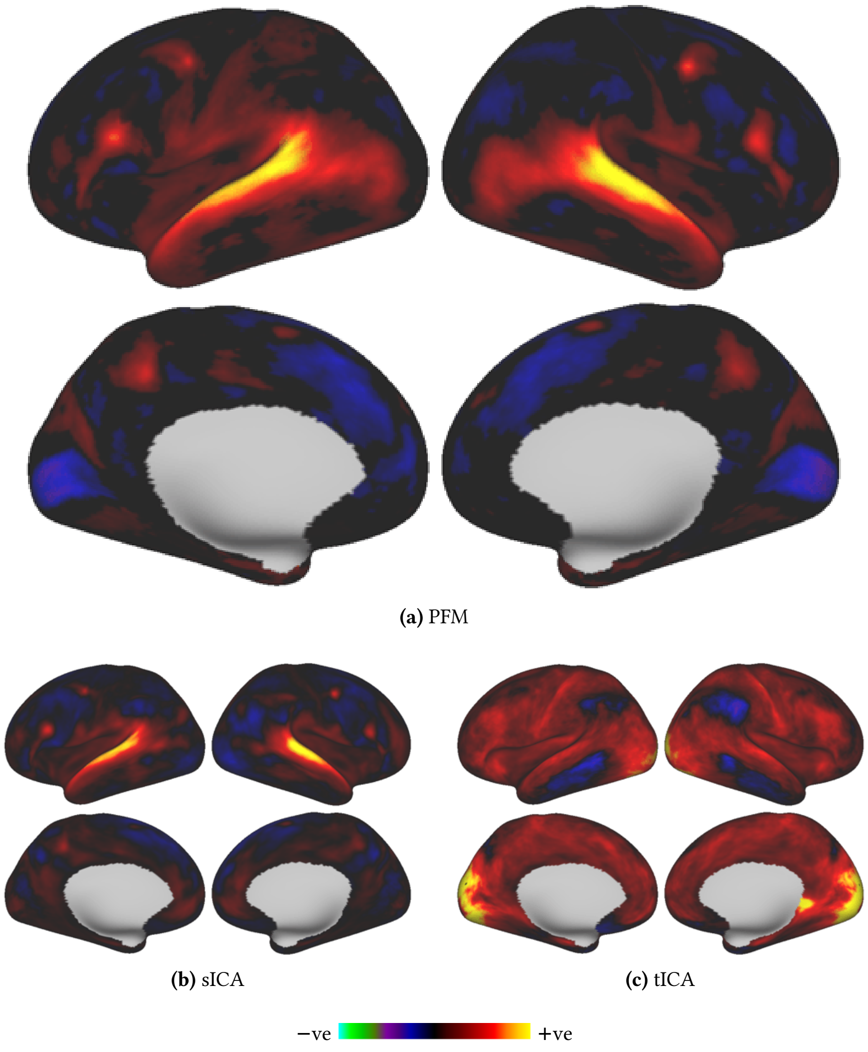

Supplementary figure S29: Component 11

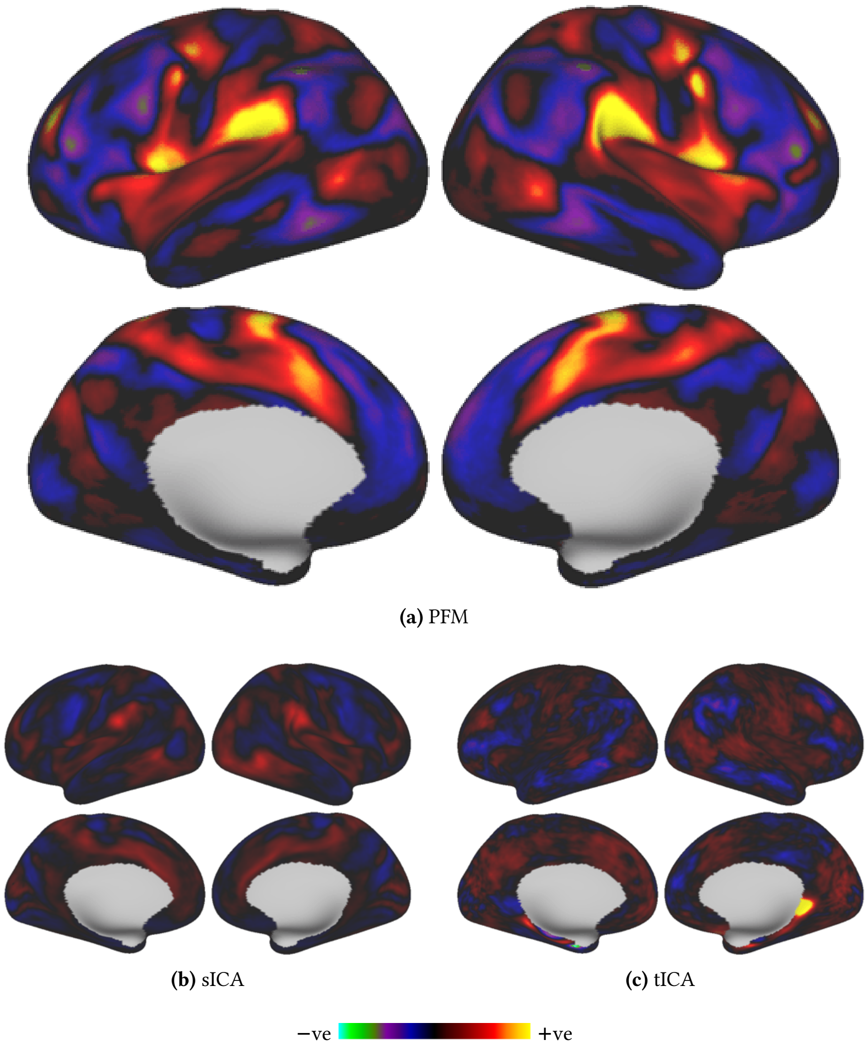

Supplementary figure S30: Component 12

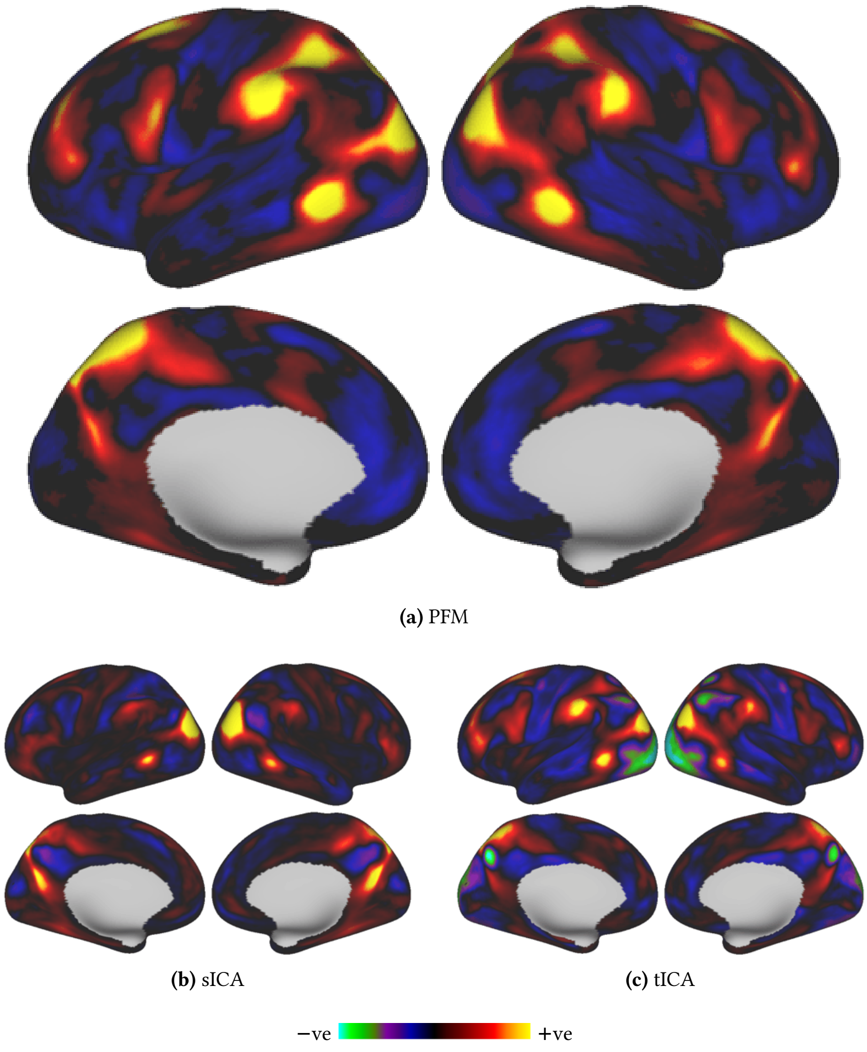

Supplementary figure S31: Component 13

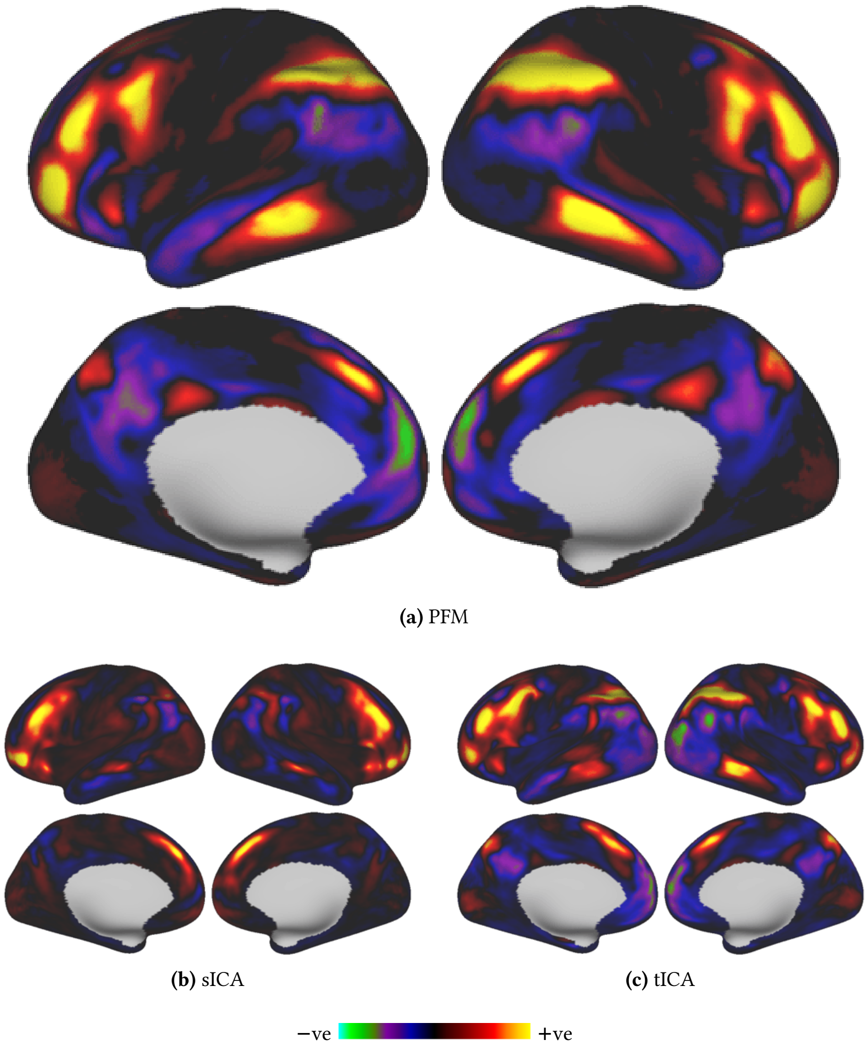

Supplementary figure S32: Component 14

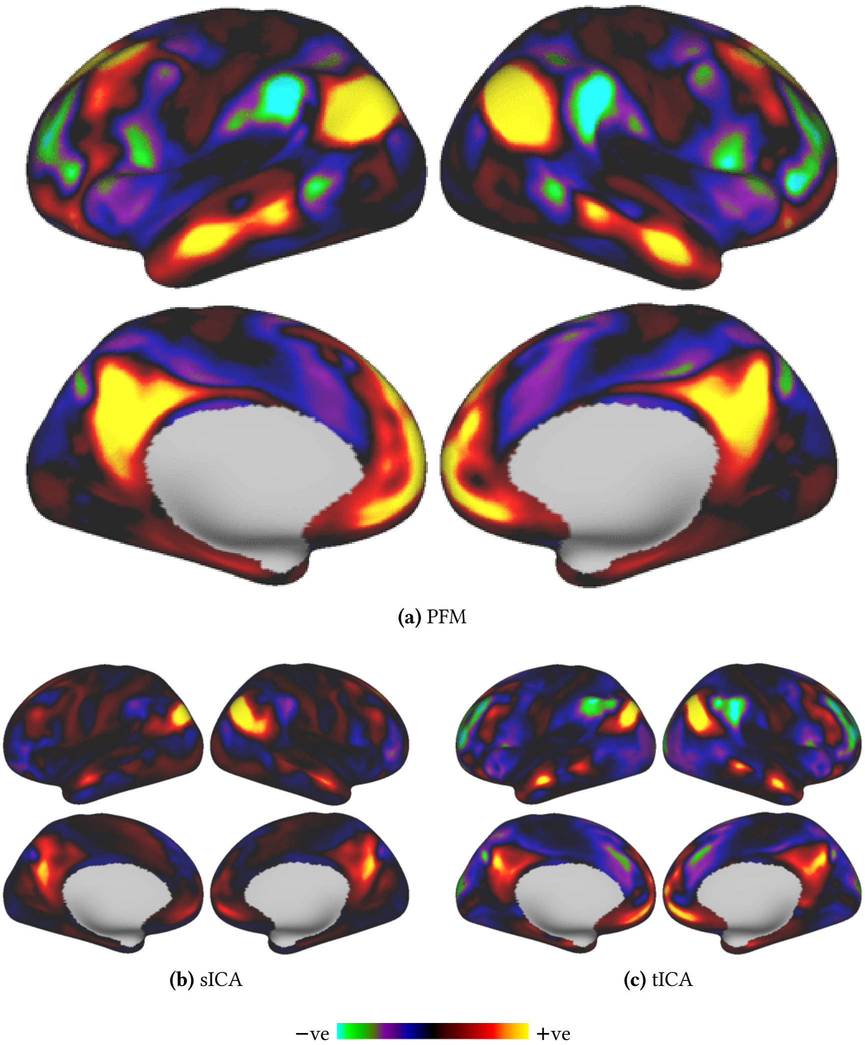

(a) PFM

(b) sICA

(c) tICA

Supplementary figure S33: Component 15

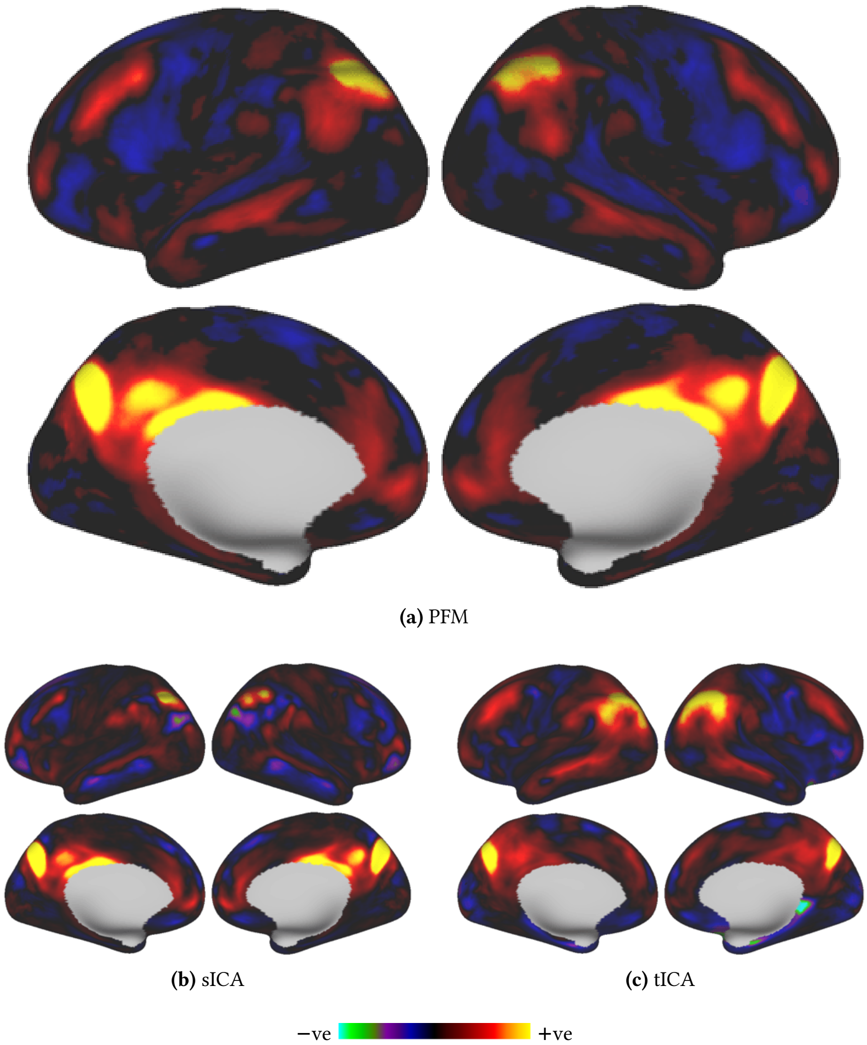

(a) PFM

(b) sICA

(c) tICA

Supplementary figure S34: Component 16

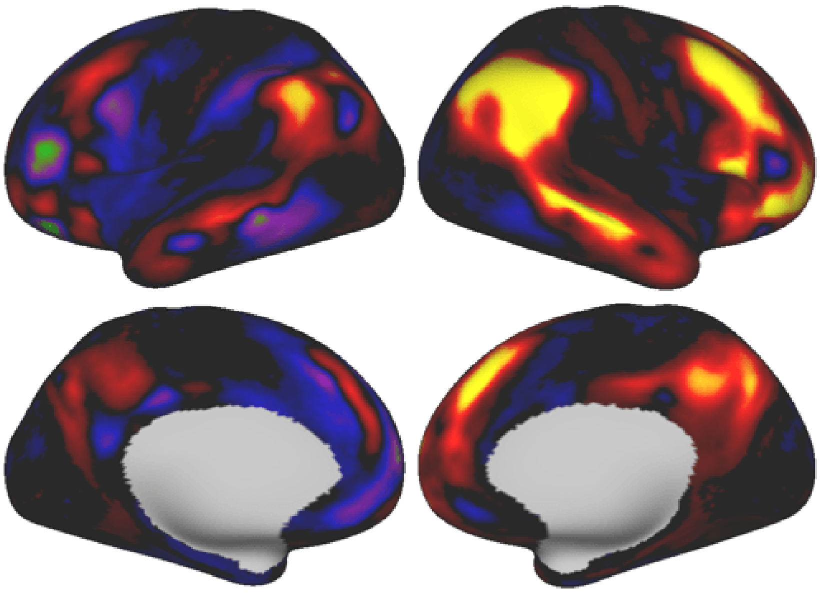

(a) PFM

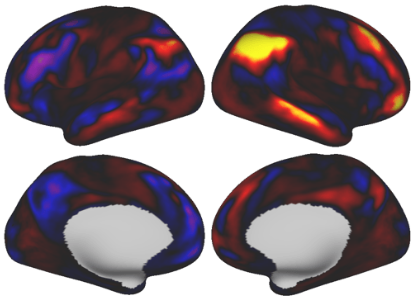

(b) sICA

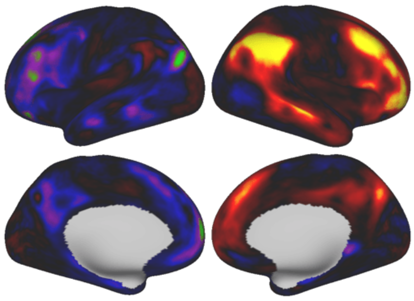

(c) tICA

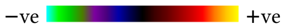

Supplementary figure S35: Component 17

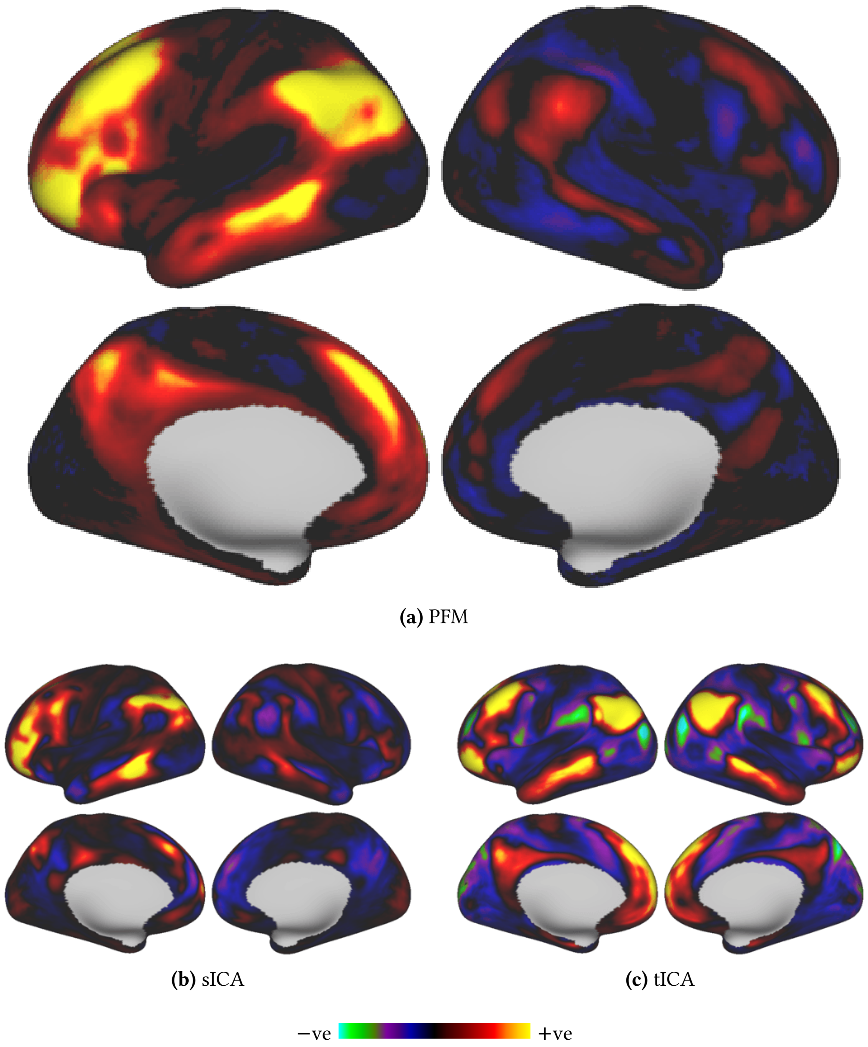

Supplementary figure S36: Component 18

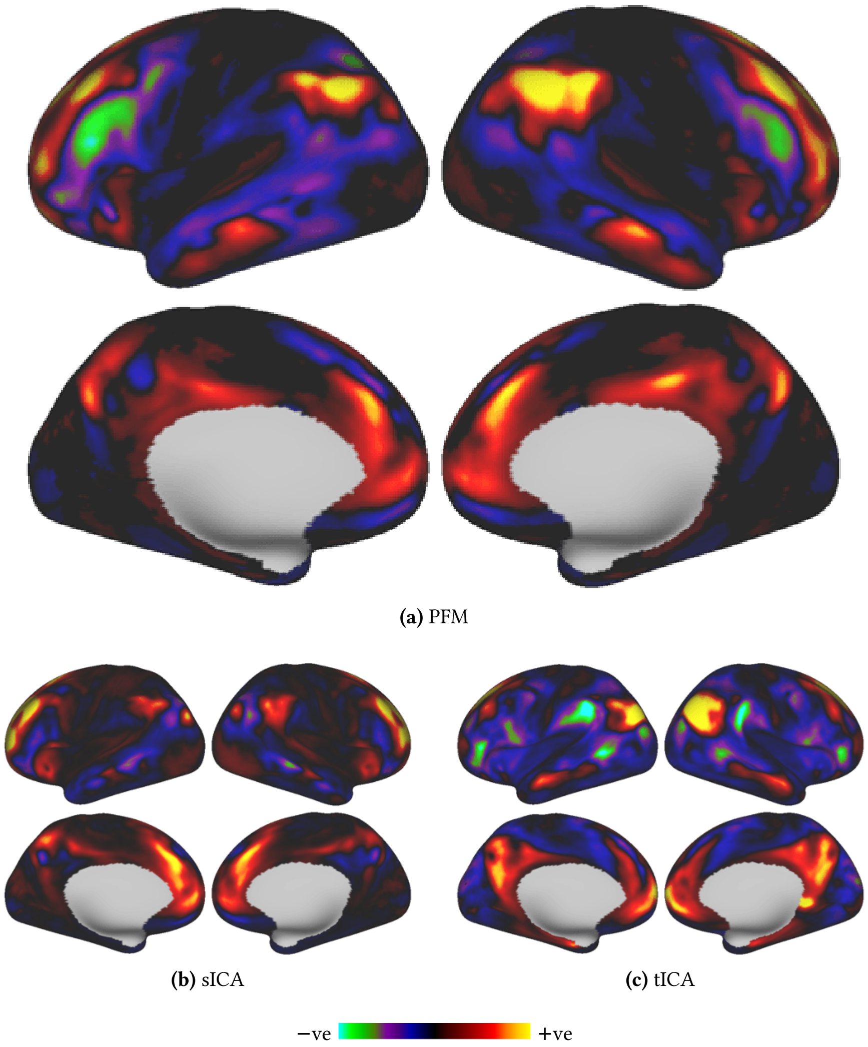

Supplementary figure S37: Component 19

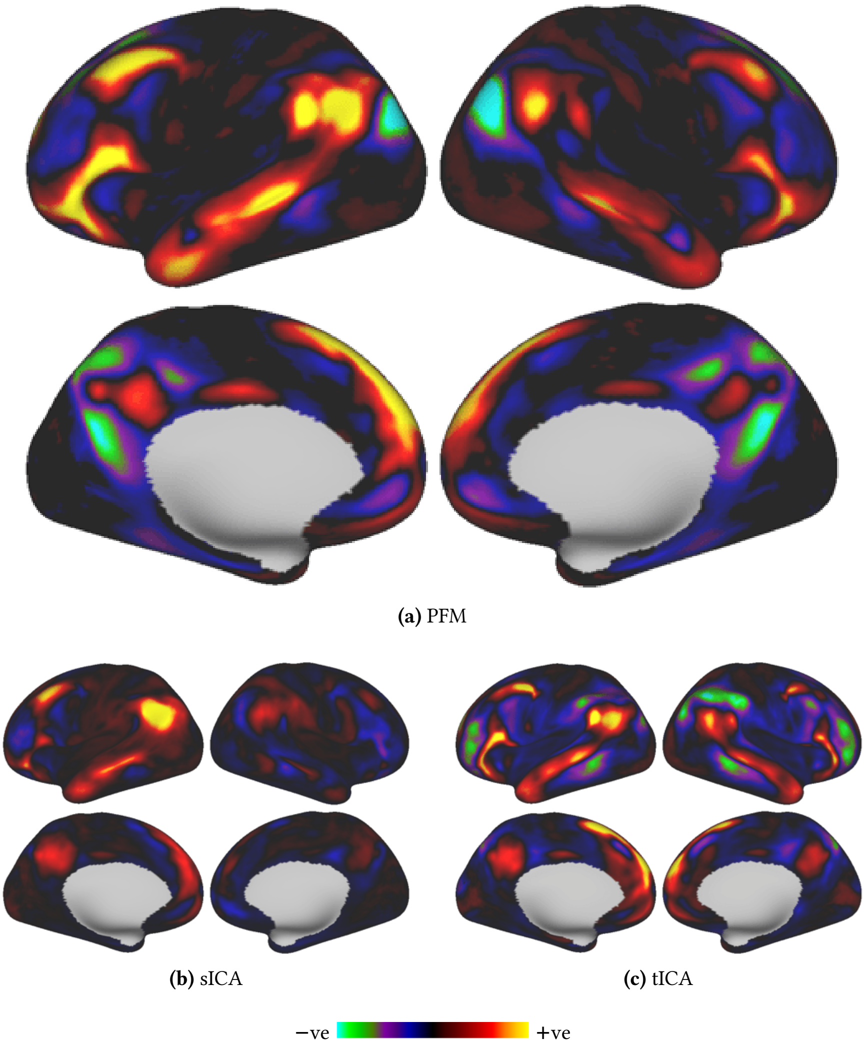

(a) PFM

(b) sICA

(c) tICA

Supplementary figure S38: Component 20

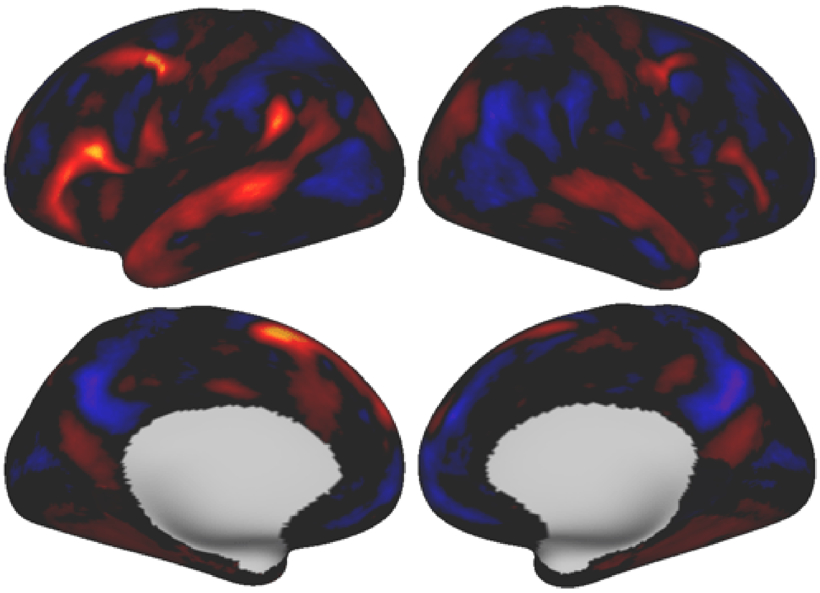

(a) PFM

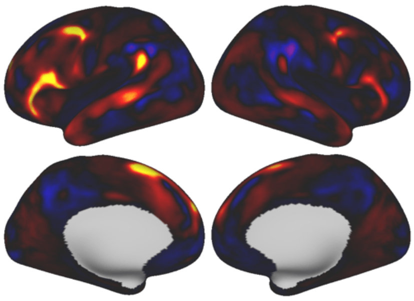

(b) sICA

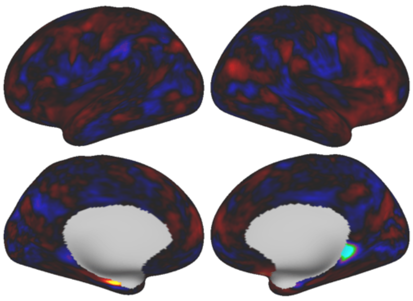

(c) tICA

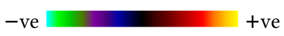

Supplementary figure S39: Component 21

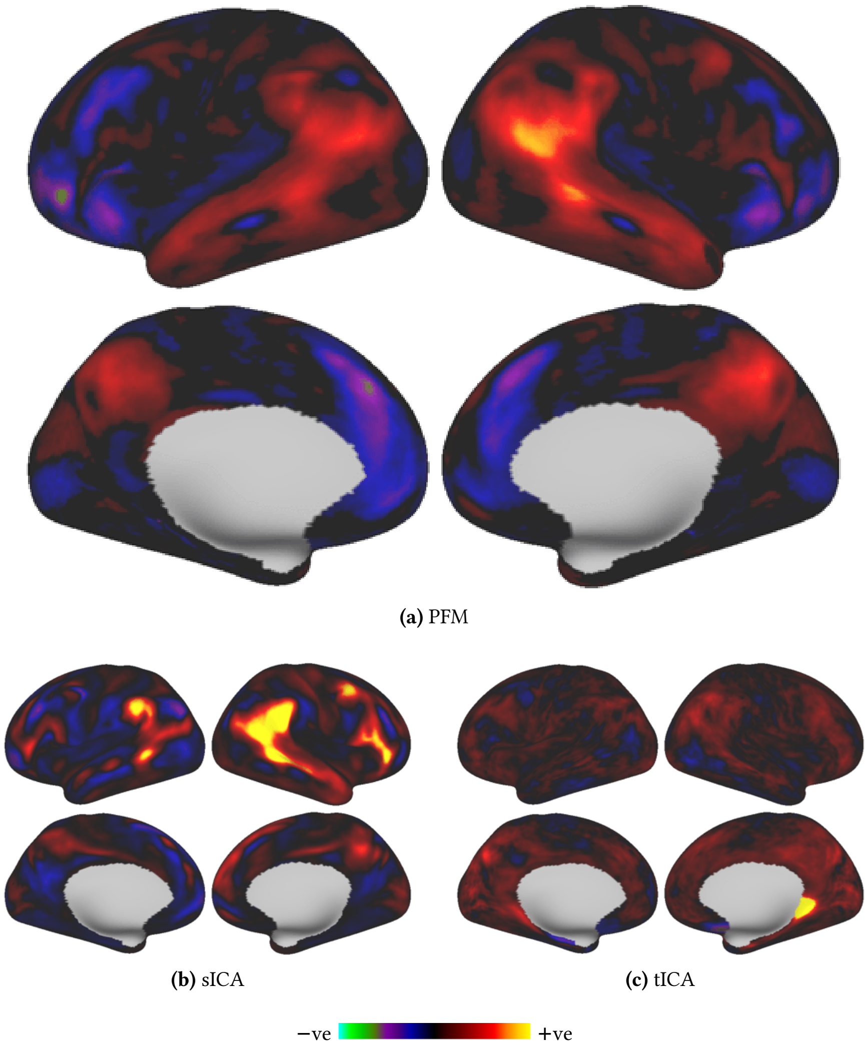

Supplementary figure S40: Component 22

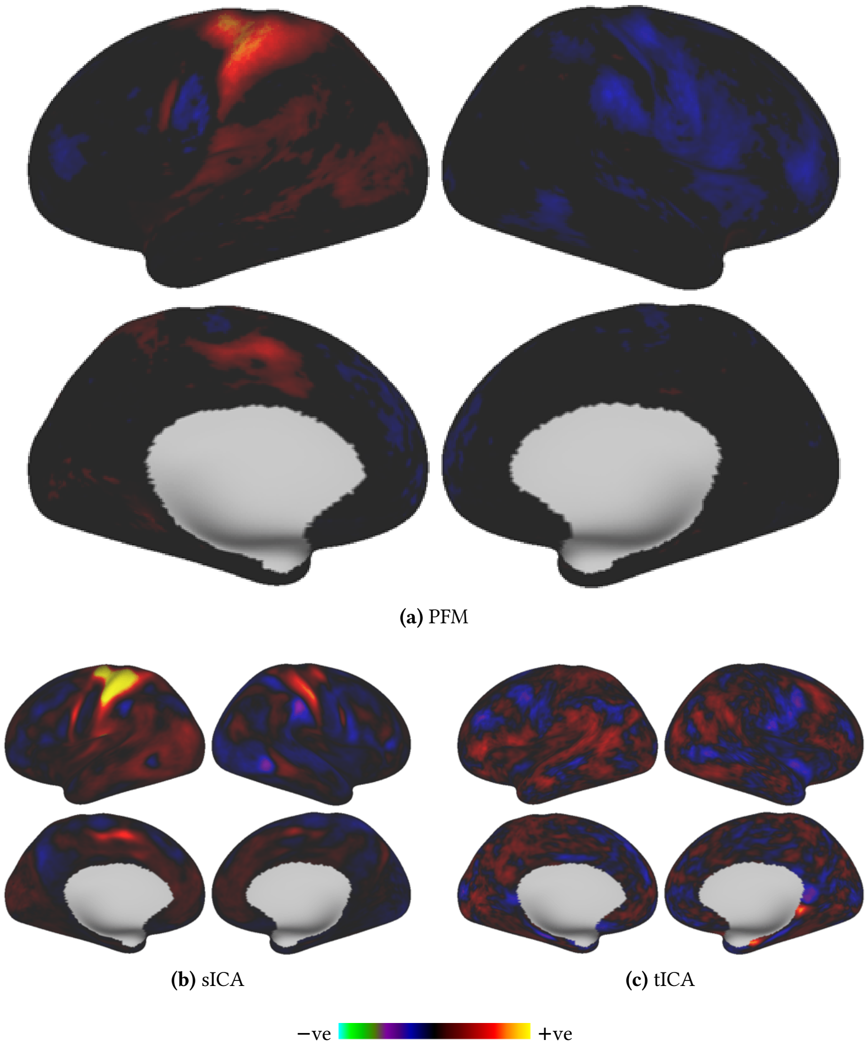

(a) PFM

(b) sICA

(c) tICA

Supplementary figure S41: Component 23

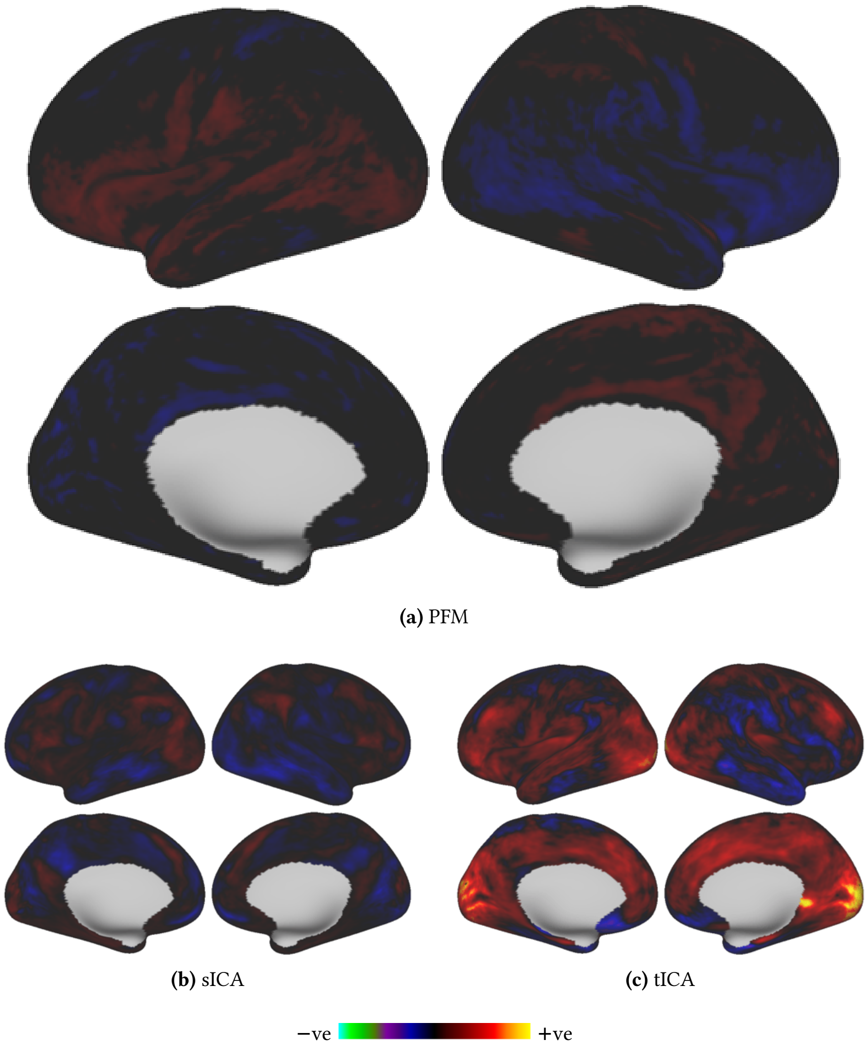

Supplementary figure S42: Component 24

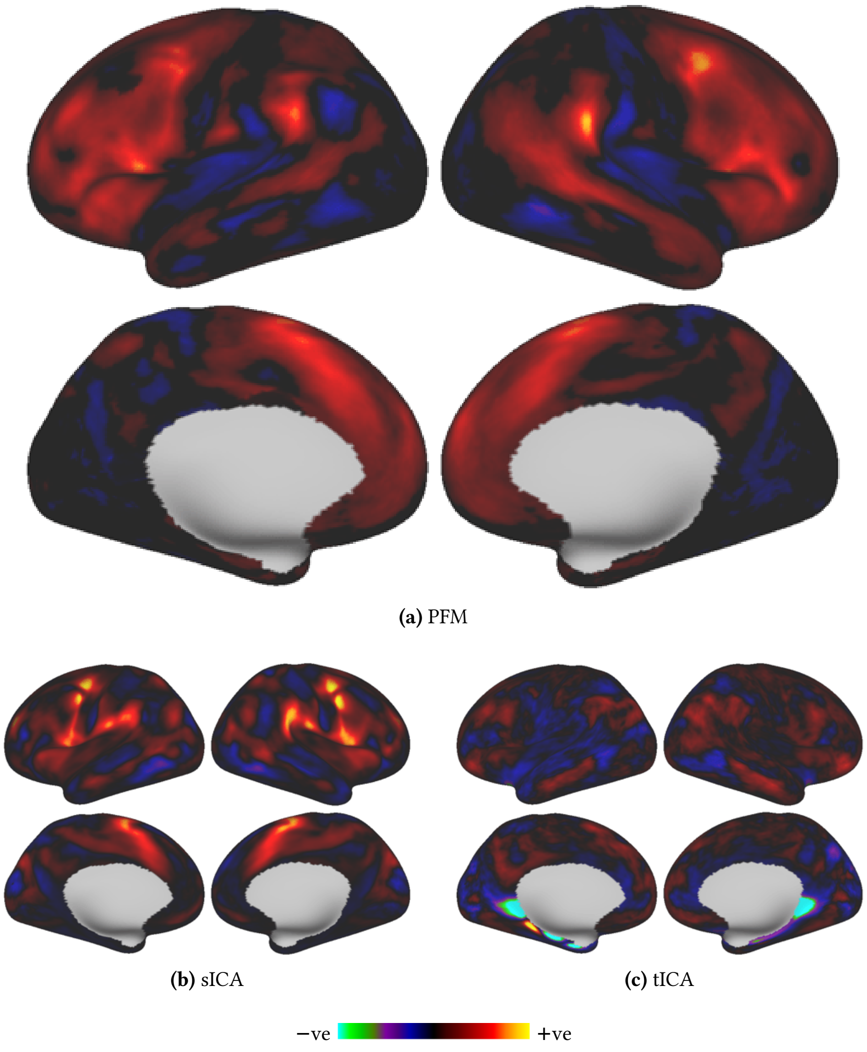

Supplementary figure S43: Component 25

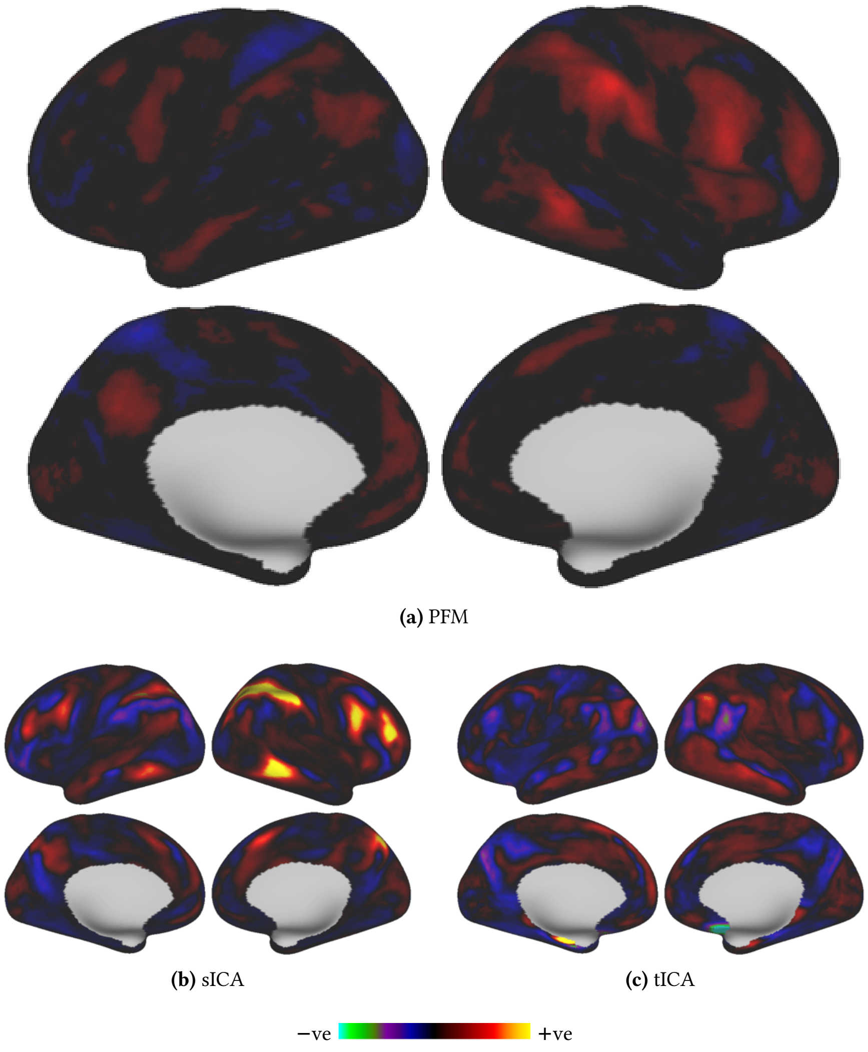

Supplementary figure S44: Component 26

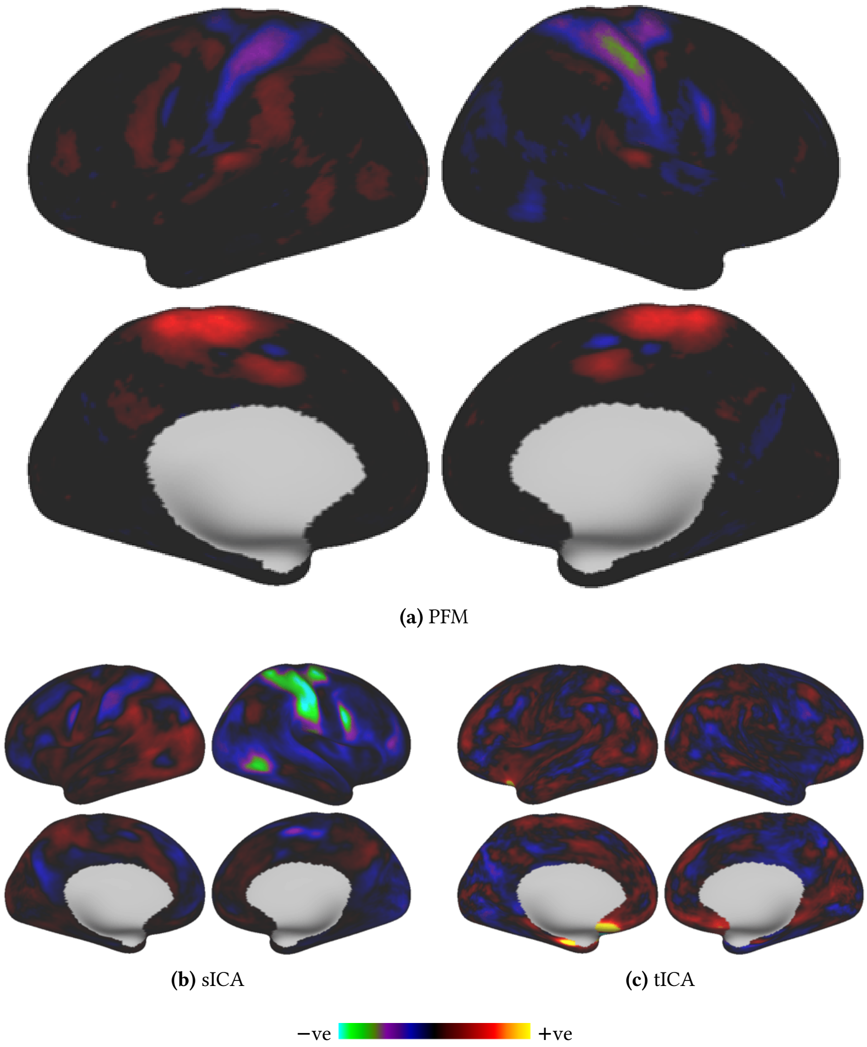

Supplementary figure S45: Component 27

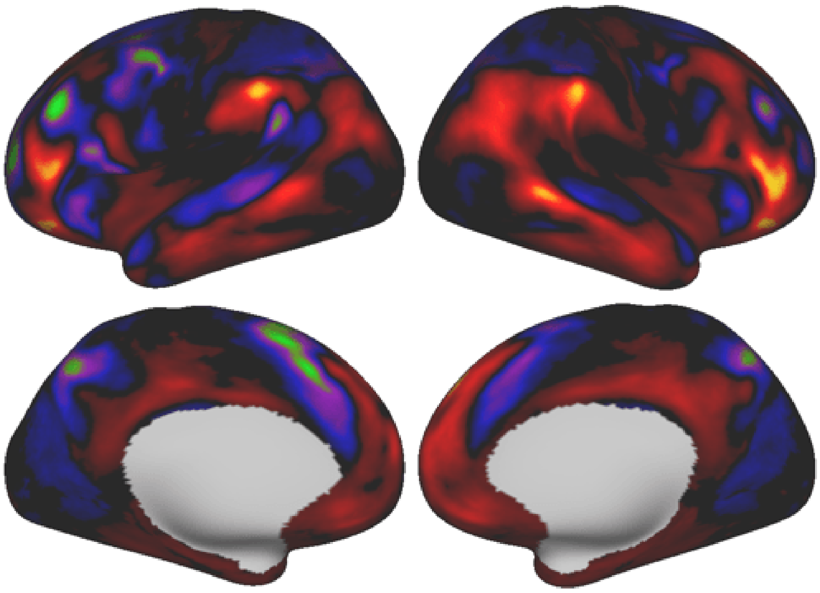

(a) PFM

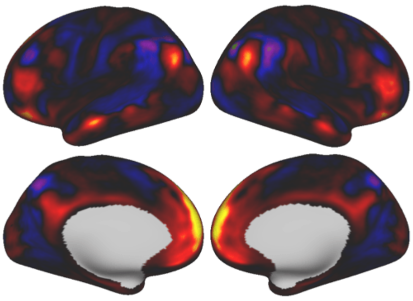

(b) sICA

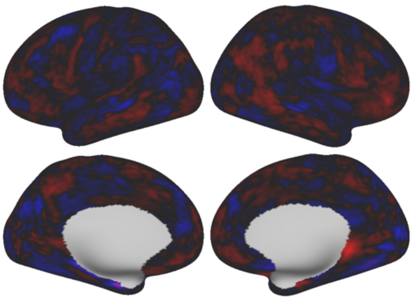

(c) tICA

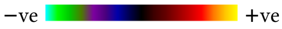

Supplementary figure S46: Component 28

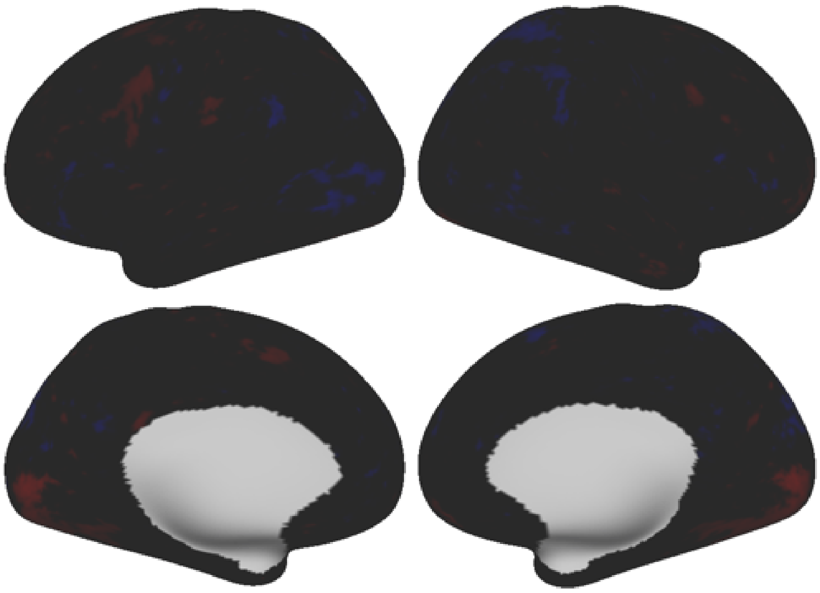

(a) PFM

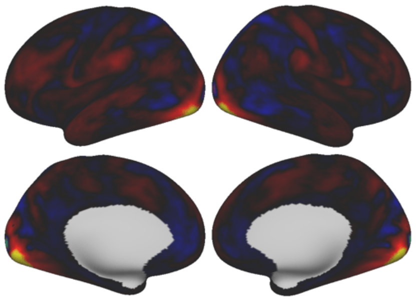

(b) sICA

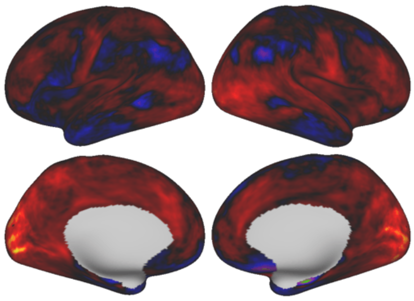

(c) tICA

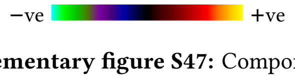

Supplementary figure S47: Component 29

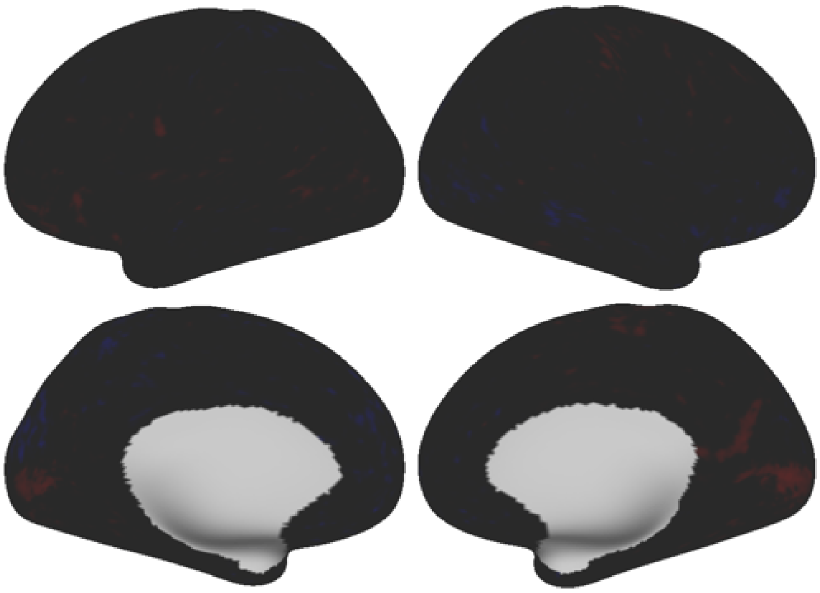

(a) PFM

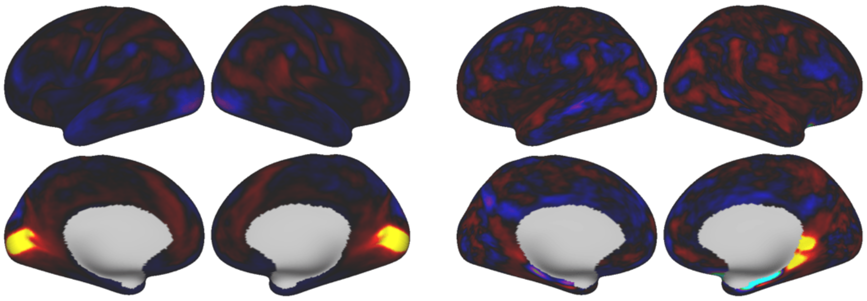

(b) sICA **(c)** tICA

−ve +ve

Supplementary figure S48: Component 30

5 Theoretical comparison of PFMs and ICA

The definitions in this section are all standard and can be found from many sources. However, the notational conventions are, for the most part, borrowed from *Independent Component Analysis* by Hyvärinen et al.[2] and *Information Theory, Inference and Learning Algorithms* by MacKay[3].

5.1 Definitions

5.1.1 Independence

Random variables X and Y are independent if and only if the joint probability density factorises, so

$$p_{X,Y}(x,y) = p_X(x)\,p_Y(y) \tag{1}$$

Therefore, for any absolutely integrable functions f and g, independent variables satisfy the following:

$$E\big[f(X)g(Y)\big] = E\big[f(X)\big]E\big[g(Y)\big] \tag{2}$$

This can be extended to more than two variables, and random vectors, to give

$$p_{\boldsymbol{X},\boldsymbol{Y},\boldsymbol{Z},\dots}(\boldsymbol{x},\boldsymbol{y},\boldsymbol{z},\dots) = p_{\boldsymbol{X}}(\boldsymbol{x})\,p_{\boldsymbol{Y}}(\boldsymbol{y})\,p_{\boldsymbol{Z}}(\boldsymbol{z})\dots \tag{3}$$

Clearly, “the components of $\boldsymbol{x}$ can be mutually dependent, while they are independent with respect to the components of the other random vectors $\boldsymbol{y}$ and $\boldsymbol{z}$”[2].

5.1.2 Correlation and covariance

In the scalar case, we will adopt the following definitions. Let the ‘similarity’ be the expectation of the product of the variables,

$$\mathrm{sim}(X,Y) = r_{X,Y} = E[XY]\ , \tag{4}$$

and the covariance be the version of this after the variables have been demeaned,

$$\begin{aligned}\mathrm{cov}(X,Y) = c_{X,Y} &= E\big[\big(X - E[X]\big)\big(Y - E[Y]\big)\big] \\ &= E[XY] - E[X]E[Y] \\ &= r_{x,y} - E[X]E[Y]\ .\end{aligned} \tag{5}$$

We will use the terms correlation and covariance interchangeably.

In the vector case, the definitions are analogous. For random vectors $\boldsymbol{X} \in \mathbb{R}^{K_X}$ and $\boldsymbol{Y} \in \mathbb{R}^{K_Y}$, with mean vectors $\boldsymbol{m}_{\boldsymbol{X}} = E[\boldsymbol{X}]$ and $\boldsymbol{m}_{\boldsymbol{Y}} = E[\boldsymbol{Y}]$ respectively, then we can define the similarity and covariance as:

$$\boldsymbol{R}_{\boldsymbol{X},\boldsymbol{Y}} = E\big[\boldsymbol{X}\boldsymbol{Y}^{\mathrm{T}}\big] \tag{6}$$

$$\begin{aligned}\boldsymbol{C}_{\boldsymbol{X},\boldsymbol{Y}} &= E\big[\big(\boldsymbol{X} - E[\boldsymbol{X}]\big)\big(\boldsymbol{Y} - E[\boldsymbol{Y}]\big)^{\mathrm{T}}\big] \\ &= \boldsymbol{R}_{\boldsymbol{X},\boldsymbol{Y}} - \boldsymbol{m}_{\boldsymbol{X}}\boldsymbol{m}_{\boldsymbol{Y}}^{\mathrm{T}}\end{aligned} \tag{7}$$

Note that in general these will not be square matrices as $\boldsymbol{R}_{\boldsymbol{X},\boldsymbol{Y}}, \boldsymbol{C}_{\boldsymbol{X},\boldsymbol{Y}} \in \mathbb{R}^{K_X \times K_Y}$.

5.1.3 Results from finite samples

The above results require enough to be known about the distributions of the random variables for the various expectations to be calculated. An alternative scenario is where we observe multiple realisations of the random variables and wish to estimate the expectations directly from the observations. As this frequently uses the same notation for slightly different quantities, we will explicitly provide some results here.

Let $\tilde{\boldsymbol{X}} \in \mathbb{R}^N$ be a *row* vector consisting of n random realisations of X,

$$\tilde{\boldsymbol{X}} = (x_1, x_2, \dots, x_N) \ . \tag{8}$$

We can average over these N samples to estimate the expectations, as

$$E\big[f(X)\big] \approx \frac{1}{N}\sum_{n=1}^{N} f(x_n) \ . \tag{9}$$

For example, if we define $\tilde{\boldsymbol{Y}}$ similarly to $\tilde{\boldsymbol{X}}$ then we can compute

$$\begin{aligned} r_{X,Y} &= E[XY] \\ \hat{r}_{X,Y} &= \frac{1}{N}\sum_{n=1}^{N} x_n y_n \\ &= \frac{1}{N}\tilde{\boldsymbol{X}}\tilde{\boldsymbol{Y}}^{\mathrm{T}} \end{aligned} \tag{10}$$

The extension to the case where we have random vectors requires that we now collect matrices of random samples. Let $\tilde{\boldsymbol{X}} \in \mathbb{R}^{K_{\boldsymbol{X}} \times N}$ be a matrix consisting of N realisations of $\boldsymbol{X} \in \mathbb{R}^{K_{\boldsymbol{X}}}$,

$$\tilde{\boldsymbol{X}} = (\boldsymbol{x}_1, \boldsymbol{x}_2, \dots, \boldsymbol{x}_N) \ , \tag{11}$$

and let $\tilde{\boldsymbol{Y}} \in \mathbb{R}^{K_{\boldsymbol{Y}} \times N}$ be similarly defined for $\boldsymbol{Y}$. Then, for example,

$$\begin{aligned} \boldsymbol{R}_{\boldsymbol{X},\boldsymbol{Y}} &= E\big[\boldsymbol{X}\boldsymbol{Y}^{\mathrm{T}}\big] \\ \hat{\boldsymbol{R}}_{\boldsymbol{X},\boldsymbol{Y}} &= \frac{1}{N}\sum_{n=1}^{N} \boldsymbol{x}_n \boldsymbol{y}_n^{\mathrm{T}} \\ &= \frac{1}{N}\tilde{\boldsymbol{X}}\tilde{\boldsymbol{Y}}^{\mathrm{T}} \ . \end{aligned} \tag{12}$$

Note that if two rows of $\tilde{\boldsymbol{X}}$ and $\tilde{\boldsymbol{Y}}$ are orthogonal then the estimated similarity between them is 0.

5.2 ICA model

ICA assumes that an observation, $\boldsymbol{d}$, is a linear mixture of underlying sources, $\boldsymbol{x}$. The observation $\boldsymbol{d}$ is a realisation of a random variable $\boldsymbol{D} \in \mathbb{R}^{K_{\boldsymbol{D}}}$, and similarly for $\boldsymbol{x}$ and $\boldsymbol{X} \in \mathbb{R}^{K_{\boldsymbol{X}}}$. For simplicity, the dimensionality of $\boldsymbol{D}$ and $\boldsymbol{X}$ are often assumed to be the same i.e. $K_{\boldsymbol{D}} = K_{\boldsymbol{X}}$. The linear mixing is defined by the mixing matrix, $\boldsymbol{G}$, so

$$\boldsymbol{d} = \boldsymbol{G}\,\boldsymbol{x} \ . \tag{13}$$

The aim is to recover $\boldsymbol{x}$, which will require the inversion of $\boldsymbol{G}$. This is possible with essentially only two assumptions: firstly, each of the elements of $\boldsymbol{X}$ are generated

independently; secondly, these are drawn from non-Gaussian distributions.

$$p(\boldsymbol{X}) = \prod_{k=1}^{K_X} p_k(\boldsymbol{X}_k) \tag{14}$$

In reality, we only observe a finite number of observations, $\{\boldsymbol{d}_n\}_{n=1}^N$, and wish to recover the set of corresponding sources, $\{\boldsymbol{x}_n\}_{n=1}^N$. We can use the sample notation, introduced in section 5.1.3, to formulate the inference problem in this finite case as a matrix factorisation.

$$\tilde{\boldsymbol{D}} = \boldsymbol{G}\,\tilde{\boldsymbol{X}}\ . \tag{15}$$

5.2.1 Correlations

By construction, the generative model states that the covariance between different sources is zero. However, this is only a very weak statement; in reality, we observe a finite set of observations and it is the correlations between the sources that we observe, given the data, that are arguably of more interest in the case of fMRI, as we shall see later.

$$\begin{aligned}\hat{\boldsymbol{R}}_{X,X} &= \frac{1}{N}\tilde{\boldsymbol{X}}\tilde{\boldsymbol{X}}^{\mathrm{T}} \\ &= \frac{1}{N}\boldsymbol{G}^{-1}\hat{\boldsymbol{R}}_{D,D}\boldsymbol{G}^{-\mathrm{T}}\end{aligned} \tag{16}$$

We can actually say surprisingly little about $\hat{\boldsymbol{R}}_{X,X}$ if we only make the two previously stated assumptions that underpin ICA.

5.2.2 Whitening

Whitening is a standard pre-processing technique for ICA algorithms, as it reduces the number of parameters to infer. A random variable is white if its covariance matrix is equal to the identity, and it is always possible to transform variables to this form. The following explains why this is useful and the implications for the sample correlations between components.

First, assume we have a whitened form of the data, $\boldsymbol{Z}$, such that $\boldsymbol{C}_{Z,Z} = \boldsymbol{I}$. This is achieved with a whitening matrix, $\boldsymbol{W}$, such that $\boldsymbol{z}_n = \boldsymbol{W}\boldsymbol{d}_n$. Equation 13 then becomes

$$\begin{aligned}\boldsymbol{z}_n &= \boldsymbol{W}\,\boldsymbol{G}\,\boldsymbol{x}_n \\ &= \boldsymbol{G}'\,\boldsymbol{x}_n\ .\end{aligned} \tag{17}$$

To see why this is a useful transformation, we need to calculate the covariance of equation 17. This yields

$$\boldsymbol{C}_{Z,Z} = \boldsymbol{G}'\,\boldsymbol{C}_{X,X}\,\boldsymbol{G}'^{\mathrm{T}}\ . \tag{18}$$

Under the generative model $\boldsymbol{C}_{X,X} = \boldsymbol{I}$, and $\boldsymbol{C}_{Z,Z} = \boldsymbol{I}$ by definition, so this simplifies to

$$\boldsymbol{G}'\boldsymbol{G}'^{\mathrm{T}} = \boldsymbol{I}\ , \tag{19}$$

or in other words, $\boldsymbol{G}'$ is an orthogonal matrix.

A good rule of thumb is that an orthogonal matrix has only half the degrees of freedom of an arbitrary matrix, so restricting the search for $\boldsymbol{G}'$ to the space of orthogonal matrices will be much more efficient than inferring $\boldsymbol{G}$.

In practice, whitening is nearly always achieved by taking the SVD, $\tilde{\boldsymbol{D}} = \boldsymbol{U}\boldsymbol{S}\boldsymbol{V}^{\mathrm{T}}$, and setting $\tilde{\boldsymbol{Z}} = \boldsymbol{V}^{\mathrm{T}}$. Dimensionality reduction can be incorporated by only selecting a subset of the SVD components. Technically, the SVD actually ensures that $\hat{\boldsymbol{C}}_{\boldsymbol{Z},\boldsymbol{Z}} = \boldsymbol{I}$, as we only observe N data points rather than the 'true' distribution for $\boldsymbol{Z}$. Given that we still restrict $\boldsymbol{G}'$ to be orthogonal, it is trivial to show that this now ensures $\hat{\boldsymbol{C}}_{\boldsymbol{X},\boldsymbol{X}} = \boldsymbol{I}$.

In summary, it makes algorithmic sense to whiten the data and infer an orthogonal mixing matrix, and this ensures that the set of sources are uncorrelated under the sample definition.

5.2.3 ICA and fMRI

When used for the analysis of fMRI data, ICA is normally interpreted in terms of the matrix factorisation formulation of equation 15. For example, spatial ICA would infer a matrix $\tilde{\boldsymbol{X}}$, of size components by voxels. These are interpreted as a set of independent spatial maps, and this is why the finite sample approximation to the source covariance, $\hat{\boldsymbol{C}}_{\boldsymbol{X},\boldsymbol{X}}$, is of particular interest—this now represents the correlations between the observed spatial maps. However, it is important to note that the model itself (equation 13) is blind to this interpretation and any spatial structure in the resulting maps essentially emerges by 'accident'.

Finally, it is well known that residual dependencies between sources often remain after ICA has converged—in fact, there are ICA models that explicitly utilise these dependencies[4,5]. In the case of fMRI, Beckmann et al. demonstrated that the restriction to uncorrelated sources may not hinder the inference of spatial maps that are highly correlated after thresholding if the data are noisy 'enough'[6].

5.3 PFM model

The PFM model is explicitly based round a matrix factorisation of fMRI data, and specifies a prior over subject specific maps:

$$\begin{aligned} p\big(\boldsymbol{P}_{vm}^{(s)} | q_{vm}^{(s)} = 1\big) &= \mathcal{N}\big(\boldsymbol{P}_{vm}^{(s)} | \mu_{vm}, \sigma_{vm}^2\big) \\ p\big(\boldsymbol{P}_{vm}^{(s)} | q_{vm}^{(s)} = 0\big) &= \delta\big(\boldsymbol{P}_{vm}^{(s)}\big) \\ p\big(q_{vm}^{(s)}\big) &= (\pi_{vm})^{q_{vm}^{(s)}} (1 - \pi_{vm})^{1 - q_{vm}^{(s)}} \end{aligned} \tag{20}$$

The prior over the mean parameters, that can be thought of as the 'group maps', takes a similar form:

$$\begin{aligned} p\big(\mu_{vm} | \rho_{vm} = 1\big) &= \mathcal{N}\big(\mu_{vm} | 0, \gamma_m^{-1}\big) \\ p\big(\mu_{vm} | \rho_{vm} = 0\big) &= \delta\big(\mu_{vm}\big) \\ p\big(\rho_{vm}\big) &= (\lambda)^{\rho_{vm}} (1 - \lambda)^{1 - \rho_{vm}} \end{aligned} \tag{21}$$

Clearly, both these and the factorised posterior distributions are independent, and in fact this independence is over both modes and voxels. However, PFMs are fundamentally different to ICA in that they define distributions over matrices, rather than assuming the matrices are collections of random realisations.

As explained in section 5.2.3, $\hat{\boldsymbol{C}}_{\boldsymbol{X},\boldsymbol{X}}$ is of particular interest for sICA, as it captures the dependencies between the maps we actually infer. However, this sampling approximation of the between-source covariance no longer makes sense in the PFM framework. If we let $\boldsymbol{P}(v, m) = \mu_{vm}\rho_{vm}$ and consider this as analogous to the ICA group maps, then we can explicitly calculate a conceptually similar quantity to $\hat{\boldsymbol{R}}_{\boldsymbol{X},\boldsymbol{X}}$,

$$\boldsymbol{R}^{*}_{\boldsymbol{P},\boldsymbol{P}} = \frac{1}{V} \sum_{v=1}^{V} E\big[\boldsymbol{P}_v^{\mathrm{T}} \boldsymbol{P}_v\big] \,. \tag{22}$$

The approximate VB posterior for these parameters is

$$\begin{aligned} q(\mu_{vm}|\rho_{vm} = 1) &= \mathcal{N}(\mu_{vm}|m_{\mu_{vm}}, \sigma^2_{\mu_{vm}}) \\ q(\mu_{vm}|\rho_{vm} = 0) &= \delta(\mu_{vm}) \\ q(\rho_{vm}) &= (\phi_{\rho_{vm}})^{\rho_{vm}}(1 - \phi_{\rho_{vm}})^{1-\rho_{vm}} \end{aligned} \tag{23}$$

Taking expectations with respect to this yields

$$\boldsymbol{R}^*_{\boldsymbol{P},\boldsymbol{P}}(i,j) = \begin{cases} \frac{1}{V}\sum_{v=1}^{V} \phi_{\rho_{vi}}(m^2_{\mu_{vi}} + \sigma^2_{\mu_{vi}}) & \text{if } i = j \\ \frac{1}{V}\sum_{v=1}^{V} (\phi_{\rho_{vi}} m_{\mu_{vi}})(\phi_{\rho_{vj}} m_{\mu_{vj}}) & \text{otherwise} \end{cases} \tag{24}$$

Therefore, while the PFM model can clearly support stable 'correlations' between the maps as a whole. The crux of the issue is that for PFMs, in contrast with ICA, each voxel in a spatial map is independently drawn from a *different* distribution. The finite sample approximation we use for ICA pools over voxels, under the assumption that they are identically distributed. If this is not the case, as with PFMs, then this metric will suggest the maps are correlated, even though they are independently distributed.

The equivalent derivations for $\boldsymbol{C}^*_{\boldsymbol{P},\boldsymbol{P}}$ can be found below, but the intuition is the same—we show $\boldsymbol{R}^*_{\boldsymbol{P},\boldsymbol{P}}$ above as it has a slightly simpler form.

If we define the voxelwise means of the PFM maps as

$$\bar{\boldsymbol{P}} = \frac{1}{V}\sum_{v=1}^{V} E[\boldsymbol{P}_v] ,$$

then we can define $\boldsymbol{C}^*_{\boldsymbol{P},\boldsymbol{P}}$ as

$$\boldsymbol{C}^*_{\boldsymbol{P},\boldsymbol{P}} = \frac{1}{V}\sum_{v=1}^{V} E\left[(\boldsymbol{P}_v - \bar{\boldsymbol{P}})^{\mathrm{T}}(\boldsymbol{P}_v - \bar{\boldsymbol{P}})\right] .$$

Taking expectations with respect to the VB posteriors yields

$$\bar{\boldsymbol{P}}(i) = \frac{1}{V}\sum_{v=1}^{V} \phi_{\rho_{vi}} m_{\mu_{vi}}$$

$$\boldsymbol{C}^*_{\boldsymbol{P},\boldsymbol{P}}(i,j) = \begin{cases} \frac{1}{V}\sum_{v=1}^{V} \phi_{\rho_{vi}}(m^2_{\mu_{vi}} + \sigma^2_{\mu_{vi}}) \\ \quad - \frac{1}{V^2}\sum_{k=1}^{V} \phi^2_{\rho_{ki}} m^2_{\mu_{ki}} & \text{if } i = j \\ \frac{1}{V}\sum_{v=1}^{V} (\phi_{\rho_{vi}} m_{\mu_{vi}})(\phi_{\rho_{vj}} m_{\mu_{vj}}) \\ \quad - \frac{1}{V^2}\left(\sum_{k=1}^{V} \phi_{\rho_{ki}} m_{\mu_{ki}}\right)\left(\sum_{l=1}^{V} \phi_{\rho_{lj}} m_{\mu_{lj}}\right) & \text{otherwise} \end{cases}$$

References

[1] M. W. Woolrich, T. E. Behrens, and S. M. Smith. "Constrained linear basis sets for HRF modelling using Variational Bayes". In: *NeuroImage* 21.4 (2004), pp. 1748–1761.

[2] A. Hyvärinen, J. Karhunen, and E. Oja. *Independent Component Analysis.* John Wiley & Sons, Inc., 2001.

[3] D. J. C. MacKay. *Information Theory, Inference and Learning Algorithms.* Cambridge University Press, 2003.

[4] A. Hyvärinen and P. O. Hoyer. “Emergence of Phase- and Shift-Invariant Features by Decomposition of Natural Images into Independent Feature Subspaces”. In: *Neural Computation* 12.7 (July 2000), pp. 1705–1720.

[5] A. Hyvärinen, P. O. Hoyer, and M. Inki. “Topographic Independent Component Analysis”. In: *Neural Computation* 13.7 (July 2001), pp. 1527–1558.

[6] C. F. Beckmann et al. “Investigations into resting-state connectivity using independent component analysis”. In: *Philosophical Transactions of the Royal Society B: Biological Sciences* 360.1457 (2005), pp. 1001–1013.
